# The epidemiology and burden of injury in countries of the Association of Southeast Asian Nations (ASEAN), 1990–2021: findings from the Global Burden of Disease Study 2021

**DOI:** 10.1016/S2468-2667(25)00069-6

**Published:** 2025-05-27

**Authors:** GBD 2021 ASEAN Injuries Collaborators, Stephanie C C van der Lubbe, Lin Siew Chong, Simon I Hay, Catherine Bisignano, Spencer L James, Xiaochen Dai, Lay Hoon Goh, Swetha Acharya, Qorinah Estiningtyas Sakilah Adnani, Naveed Ahmed, Budi Aji, Joseph Uy Almazan, Carl Abelardo T Antonio, Sumadi Lukman Anwar, Muhammad Shahzad Aslam, Atif Amin Baig, Amiel Nazer C Bermudez, Ahmad Naoras Naoras Bitar, Muthia Cenderadewi, Hana Chen, Nicholas WS Chew, Bryan Chong, Mayank Dalakoti, Hisham Atan Edinur, Crystal Amiel M Estrada, Emerito Jose Aquino Faraon, Nelsensius Klau Fauk, Ida Fitriana, Ni Kadek Yuni Fridayani, Fernando Barroga Garcia, Arief Hargono, Eka Mishbahatul Marah Has, Paul Michael Rafa Hernandez, Nahlah Elkudssiah Ismail, Vikash Jaiswal, Roland Dominic G Jamora, Jost B Jonas, Kehinde Kazeem Kanmodi, Inn Kynn Khaing, Yun Jin Kim, Chong-Han Kua, Maria Dyah Kurniasari, Asep Kusnali, Dian Kusuma, Hilton Lam, Shaun Wen Huey Lee, Zheng Feei Ma, Roy Rillera Marzo, Kamarul Imran Musa, Firzan Nainu, Dina Nur Anggraini Ningrum, Sok King Ong, Veincent Christian Filipino Pepito, Thantrira Porntaveetus, Dimas Ria Angga Pribadi, Sheena Ramazanu, Bedanta Roy, Yoseph Leonardo Samodra, Siddharthan Selvaraj, Shazlin Shaharudin, Robert Sinto, Solikhah Solikhah, Chandrashekhar T Sreeramareddy, Vetriselvan Subramaniyan, Thitiporn Sukaew, Ingan Ukur Tarigan, Jansje Henny Vera Ticoalu, Narayanaswamy Venketasubramanian, Taweewat Wiangkham, Anggi Lukman Wicaksana, Siyan Yi, Mustafa Z Younis, Christopher J L Murray, Kanyin Liane Ong, Yee Wei Lim, Marie Ng

## Abstract

**Background:**

Injuries are among the top causes of mortality and disability in southeast Asia. Although injury prevention is a key health priority in the Post-2015 Health Development Agenda of the Association of Southeast Asian Nations (ASEAN), the focus was placed solely on road injuries. The absence of a broader recognition of injury burden and trends hinders future intervention efforts. This study aims to provide a comprehensive analysis of the burden and epidemiological trends of all injuries across ASEAN countries.

**Methods:**

In this analysis of the Global Burden of Disease, Injuries, and Risk Factors Study (GBD) 2021, we estimated incidence, cause-specific mortality, years of life lost (YLLs), years lived with disability (YLDs), and disability-adjusted life-years (DALYs) by age, sex, location, and year for ten ASEAN member states (Brunei, Cambodia, Indonesia, Myanmar, Laos, Malaysia, the Philippines, Singapore, Thailand, and Viet Nam) from 1990 to 2021. Incidence and non-fatal disease burden were estimated using disease model Bayesian meta-regression (DisMod-MR) 2.1. Mortality was derived from the GBD Cause of Death Ensemble model. Estimates include 95% uncertainty intervals where appropriate.

**Findings:**

In 2021, an estimated 35·5 million (95% UI 33·5–37·7) injury incident cases were reported in ASEAN, resulting in approximately 317 000 deaths (286 000–350 000). Substantial variation was observed across the region, with the age-standardised mortality ranging from 13·4 per 100 000 people (12·7–14·1) in Singapore to 68·5 per 100 000 (54·4–81·9) in Viet Nam. Road injury was the leading cause of mortality and morbidity in most ASEAN countries, with the highest age-standardised DALY rates in Thailand and Malaysia. Self-harm was the leading cause of mortality in Singapore, whereas interpersonal violence was the leading cause of injury deaths in the Philippines. From 1990 to 2021, the region's age-standardised injury incidence rate declined by 23·0% (21·8–24·1). Age-standardised DALY rates decreased substantially for drowning (60·6% [53·2–66·7]) and road injuries (39·6% [32·1–46·4]), whereas falls saw a smaller and more heterogeneous decline of 12·3% (2·6–21·0) over the past 31 years.

**Interpretation:**

The injury epidemiological landscape in ASEAN is complex, with substantial geographical variations and emerging challenges triggered by the rapid sociodemographic transition in the region. Progress has been uneven. Effective burden reduction across different causes of injury requires strategies addressing the wide range of socioenvironmental determinants and system shortfalls. Prevention programmes need to be customised to each country's unique context and development.

**Funding:**

Bill & Melinda Gates Foundation.

## Introduction

Injuries are a major global public health concern, resulting in 4·34 million deaths and 247·8 million disability-adjusted life-years (DALYs) in 2021.^1^, ^2^ More than 85% of injury-related deaths occurred in low-income and middle-income countries,^3^ where preventive measures are limited and emergency medical services and surgical care are restricted.^4^, ^5^ Compared with other conditions such as infectious diseases and maternal and child health, injuries have yet to receive sufficient attention in these countries.^6^

Injury prevention is one of the health priorities in the Association of Southeast Asian Nations (ASEAN).^7^ ASEAN is a geopolitical and economic union consisting of ten countries—Brunei, Cambodia, Indonesia, Myanmar, Laos, Malaysia, the Philippines, Singapore, Thailand, and Viet Nam. Although Brunei and Singapore are classified as high-income countries, all other member states are considered either lower-middle-income or upper-middle-income countries according to the World Bank's definition.^8^ With an annual gross domestic product growth of 4·5%,^9^ ASEAN is one of the fastest-developing regions in the world, both socially and economically.^10^ With rapid socioeconomic development bringing about accelerated urbanisation, motorisation, and industrialisation,^11^ injuries remain a stubborn public health challenge for the region.^12^, ^13^ In the past 30 years, although considerable progress has been made in addressing communicable, maternal, neonatal, and nutritional diseases—with an estimated reduction of 66·7% in DALYs per 100 000—progress in reducing injury burden has been less pronounced, with merely a 40·2% decline in DALYs.^14^ In eight of the ten ASEAN countries, injuries ranked among the top-ten causes of disease burden in 2021.^14^

**Research in context**

**Evidence before this study**

We searched PubMed for English-language articles published between Jan 1, 1990, and Oct 23, 2024, using compound search terms (appendix p 3) combined with country-specific terms (“ASEAN” OR “Brunei” OR “Cambodia” OR “Indonesia” OR “Laos” OR “Malaysia” OR “Myanmar” OR “Philippines” OR “Singapore” OR “Thailand” OR “Viet Nam”). Studies were included if they reported injury-related mortality, morbidity, or disability-adjusted life-years in the Association of Southeast Asian Nations (ASEAN) countries, regardless of study design. We excluded studies focusing solely on clinical management or rehabilitation outcomes. Previous research on injury burden in ASEAN has been scarce, often focusing on a single country, specific injury cause, or particular age group. Thailand, Malaysia, Singapore, and Viet Nam have been studied most extensively, particularly regarding road injuries. By contrast, few studies have been done in Brunei, Cambodia, Laos, and Myanmar. Studies related to self-harm and interpersonal violence have been identified in Thailand, the Philippines, and Viet Nam. From the grey literature, publications from the ASEAN Secretariat have provided general overviews of selected causes of injuries within the region. To date, no study has comprehensively examined the epidemiology and burden of injuries across the entire ASEAN region.

**Added value of this study**

There is a clear evidence gap in quantifying the incidence and disease burden of injuries in ASEAN. This study offers the first comprehensive analysis of the incidence, mortality, and morbidity associated with injury at both national and regional levels across ASEAN. Our study systematically assessed the geographical and temporal trends, and the variations in age and sex. These trends and variations reveal disparities across countries, highlighting where targeted interventions are necessary at the national level. The study provides evidence for prioritisation of preventive actions, guided by injuries that have the highest disease burden. Strategic cooperation must be strengthened at the regional level.

**Implications of all the available evidence**

The burden of injury in ASEAN is substantial. National and regional strategies for injury prevention and management must be expanded to encompass the diverse range of injury causes affecting different populations and to align these strategies with the broader public health initiatives.

In the ASEAN Post-2015 Health Development Agenda (APHDA) 2021–25 5-year plan,^15^ all ten ASEAN countries have declared injury prevention as one of the key health priorities for the region. Despite this recognition, there is a scarcity of robust data on the trends and status of injury epidemiology and burden. To document progress and support policy refinement for the post-2025 health agenda, this study provides a comprehensive examination of the prevalence, mortality, and morbidity of three broad causes of injuries by age, sex, and location across all ASEAN countries from 1990 to 2021. The evidence presented can serve as an empirical foundation for prioritising and contextualising individual countries’ efforts in injury-prevention policies. This Article was produced as part of the Global Burden of Disease, Injuries, and Risk Factors Study (GBD) Collaborator Network and in accordance with the GBD Protocol.^16^

## Methods

### Overview

As part of the Global Burden of Disease, Injuries, and Risk Factors Study (GBD) 2021, this research examines the incidence, mortality, and morbidity of injuries at both the regional and national levels across ASEAN. GBD is a global epidemiological study that systematically evaluates 371 diseases and injuries, along with 88 risk factors, across 204 countries and territories.^1^, ^17^ The study produces consistent estimates of population health metrics, including incidence, prevalence, cause-specific deaths, years of life lost (YLLs), years lived with disability (YLDs), and DALYs by age, sex, location, and year from 1990 to 2021.

### Case definitions

GBD defines injuries as death or disability due to the direct or indirect result of a physical force, immersion, or exposure, including accidental, interpersonal, or self-inflicted forces as well as war, conflict, violence, and natural disasters.^18^ There are 17 distinct external level 3 cause categories of injury included in GBD 2021 (disaggregated from three level 2 injury cause groups: unintentional injuries; self-harm and interpersonal violence; and transport injuries), which are further divided into 47 unique natures of injury. In this study, we focus on major cause categories. The definitions of injury incidence and death were based on the International Classification of Diseases (ICD): codes E000–E999 in ICD-9 and codes V01–Y98 in ICD-10. In addition, ICD-9 codes 800–999 and ICD-10 codes S00–T98 were used to ascertain injury morbidity. Details of the injury cause hierarchy can be found in the appendix (p 4), and codes used for classifying injury can also be found in the appendix (pp 5–7) and in the GBD 2021 online database.^19^

### Mortality and YLLs

Cause-specific mortality is estimated for every cause of injury except for foreign body in the eyes and sexual violence. Within the GBD framework, data used for the estimation of injury mortality included vital registration, vital registration samples, verbal autopsy, police records, and mortuary and hospital data. Data sources used for the analysis for each ASEAN country are detailed in the appendix (pp 8–15). The GBD Cause of Death Ensemble model method was applied to generate cause-specific mortality estimates by age, sex, location, year, and cause of injury. Further details can be found in the appendix (p 17) and in previous publications.^1^ Subsequently, YLLs, which quantify health loss due to premature death, were calculated by multiplying the number of deaths due to each specific cause by the residual life expectancy at the corresponding age. In addition to the total YLLs estimates, YLL rates and age-standardised mortality rates over time were derived. Details of age-standardisation calculations can be found in the appendix (p 18) and additional information on injury mortality modelling can be found in previous publications.^2^, ^20^

### Incidence, prevalence, YLDs, and DALYs

Non-fatal outcomes of injuries were defined as those requiring medical care, given that minor injuries were assumed to have no disability burden. The incidence of injuries was estimated using a disease model—Bayesian meta-regression (DisMod-MR) 2.1, an analytical tool that synthesises data based on a compartmental model structure to retain coherent relationships across different metrics. Briefly, DisMod-MR 2.1 employs Bayesian meta-regression techniques to integrate epidemiological data from various sources. The list of non-fatal injury data sources from ASEAN countries can be found in the appendix (pp 16–17). Further details on DisMod-MR 2.1 can be found in the appendix (p 17) and previous GBD publications.^2^

The estimation of prevalence and YLDs began with determining the disability associated with a specific cause of injury, based on the distribution of the nature of injuries. The nature of injuries represented bodily harm that could result from a given injury cause.^20^ Clinical data sources containing both cause and nature of injuries were used to determine the probability of the nature of injuries for each new injury case and were applied to the total incidence to establish the cause–nature distribution. Additional analyses were done to convert incidence to prevalence (appendix pp 17–18). YLDs for each cause of injury were calculated as the prevalence of each cause–nature multiplied by the corresponding disability weight,^21^, ^22^ then summed across the nature of injuries for each cause by age, sex, location, and year. Cause-specific DALYs were calculated by summing YLLs and YLDs for each cause, reflecting both the fatal and non-fatal effects of injuries on health outcomes. Measurement errors and predictive uncertainties were propagated through each analytical stage to derive the final estimates. DisMod-MR uses Markov Chain Monte Carlo sampling to obtain draws from the posterior distribution, and these draws were used to compute a 95% uncertainty interval for the estimates. Further details can be found in previous publications.^2^, ^20^

### Role of the funding source

The funder of the study had no role in study design, data collection, data analysis, data interpretation, or writing of the report.

## Results

In 2021, there were an estimated 35·5 million injury incident cases (95% UI 33·5–37·7) in ASEAN (table 1), resulting in 317 000 deaths (286 000–350 000), which accounted for 5·9% (5·3–6·5) of total deaths (table 2). The number of DALYs attributed to injuries was estimated at 18·4 million (16·7–20·2), representing 8·1% (7·4–8·7) of all-cause DALYs in the region (table 3). The age-standardised rates per 100 000 were estimated as 5262·0 (4963·2–5581·9) for incidence, 49·0 (44·1–53·5) for mortality, and 2702·1 (2451·5–2964·4) for DALYs (Table 1, Table 2, Table 3).

**Table 1:** Number of incidence cases (in thousands), proportion of cases relative to all-cause incidence, and age-standardised incidence rates per 100 000 people due to injuries in 2021, along with changes from 1990 to 2021, for ASEAN and its member states

	**Incidence in 2021**			**Change in incidence from 1990 to 2021**		
	Number of cases, in thousands	Percent of all-cause incidence	Age-standardised incidence rates, per 100 000 people	Percentage change in number of cases, in thousands	Percentage change in percent of all-cause incidence	Percentage change in age-standardised incidence rates
ASEAN	35 500 (33 500 to 37 700)	1·1% (1·0 to 1·2)	5262·0 (4963·2 to 5581·9)	11·4% (8·9 to 13·9)	−17·8% (−20·1 to −15·2)	−23·0% (−24·1 to −21·8)
Brunei	64·0 (59·5 to 68·4)	3·4% (3·1 to 3·8)	13 921·8 (12 911·8 to 14 897·5)	36·1% (32·2 to 40·3)	−16·9% (−20·8 to −12·9)	−18·1% (−19·9 to −16·2)
Cambodia	948 (895 to 1000)	1·3% (1·2 to 1·4)	5589·2 (5287·5 to 5892·7)	29·0% (23·7 to 34·1)	−6·2% (−11·3 to −1·0)	−17·9% (−20·7 to −15·2)
Indonesia	12 300 (11 500 to 13 200)	0·8% (0·8 to 0·9)	4523·9 (4214·3 to 4846·7)	1·0% (−1·6 to 3·8)	−24·2% (−26·9 to −21·1)	−28·5% (−29·7 to −27·1)
Laos	350 (331 to 372)	1·0% (0·9 to 1·1)	4632·5 (4389·1 to 4899·3)	5·3% (−12·6 to 18·7)	−32·2% (−43·4 to −22·8)	−39·1% (−48·4 to −32·1)
Malaysia	1510 (1420 to 1610)	1·0% (0·9 to 1·1)	4675·0 (4398·8 to 4968·4)	60·8% (55·8 to 65·7)	−19·4% (−23·0 to −15·1)	−10·8% (−12·7 to −8·6)
Myanmar	4310 (3960 to 4790)	1·9% (1·7 to 2·1)	7532·8 (6927·2 to 8338·3)	31·8% (21·6 to 44·8)	5·0% (−3·4 to 15·6)	−1·7% (−8·9 to 8·1)
Philippines	5500 (5150 to 5880)	1·0% (0·9 to 1·0)	4752·7 (4466·0 to 5058·0)	13·6% (10·7 to 16·5)	−25·6% (−28·3 to −22·5)	−35·0% (−36·3 to −33·7)
Singapore	634 (580 to 688)	2·7% (2·5 to 3·0)	12 656·1 (11 406·8 to 13 880·7)	28·8% (23·1 to 34·3)	−29·2% (−32·9 to −25·1)	−16·2% (−18·9 to −13·6)
Thailand	3670 (3510 to 3850)	1·1% (1·0 to 1·2)	5674·0 (5358·8 to 5981·7)	−17·5% (−20·5 to −14·3)	−23·8% (−27·9 to −19·2)	−23·3% (−25·4 to −21·1)
Viet Nam	6160 (5830 to 6500)	1·5% (1·4 to 1·6)	6234·8 (5887·2 to 6564·2)	35·5% (29·9 to 40·7)	−1·1% (−6·6 to 4·3)	−6·7% (−9·6 to −3·7)

Data n (95% UI), % (95% UI), and rate (95% UI) ASEAN=Association of Southeast Asian Nations. UI=uncertainty interval.

**Table 2:** Number of deaths, proportion of deaths relative to all-cause mortality, and age-standardised death rates per 100 000 people due to injuries in 2021, along with changes from 1990 to 2021, for ASEAN and its member states

	**Deaths in 2021**			**Change in deaths from 1990 to 2021**		
	Number of deaths (95% UI)	Percent of all-cause mortality	Age-standardised mortality rates, per 100 000 people	Percentage change in number of deaths	Percentage change in percent of all-cause mortality	Percentage change in age-standardised mortality rates
ASEAN	317 000 (286 000 to 350 000)	5·9% (5·3 to 6·5)	49·0 (44·1 to 53·5)	6·9% (−3·7 to 19·5)	−35·0% (−42·9 to −28·3)	−35·9% (−41·7 to −28·8)
Brunei	122 (109 to 135)	6·6% (6·2 to 7·1)	29·9 (26·8 to 33·1)	−5·8% (−18·7 to 8·7)	−46·1% (−50·2 to −42·2)	−52·2% (−58·8 to −44·9)
Cambodia	9410 (7280 to 12 000)	7·3% (6·3 to 8·2)	67·1 (53·1 to 84·0)	15·4% (−11·4 to 51·6)	2·3% (−13·6 to 19·2)	−30·0% (−44·9 to −10·8)
Indonesia	92 500 (79 000 to 111 000)	4·2% (3·5 to 4·9)	38·4 (32·6 to 45·6)	−1·0% (−16·8 to 20·6)	−38·1% (−49·8 to −26·9)	−35·0% (−45·0 to −22·7)
Laos	3520 (2710 to 4480)	6·9% (6·0 to 7·7)	54·4 (42·4 to 68·1)	−12·5% (−35·0 to 18·2)	5·5% (−11·7 to 24·3)	−49·1% (−61·9 to −32·3)
Malaysia	14 800 (14 000 to 15 700)	6·6% (6·1 to 7·1)	47·7 (45·0 to 50·8)	68·4% (56·5 to 85·2)	−37·4% (−42·9 to −30·4)	−23·2% (−29·1 to −14·7)
Myanmar	30 900 (25 800 to 37 300)	6·1% (5·1 to 7·0)	58·7 (49·7 to 70·4)	−20·7% (−37·3 to 2·2)	−22·4% (−35·1 to −8·8)	−44·2% (−55·3 to −28·7)
Philippines	48 600 (41 700 to 56 300)	5·5% (5·1 to 5·9)	46·4 (39·9 to 53·8)	21·8% (2·9 to 43·4)	−50·2% (−54·3 to −46·0)	−33·9% (−44·2 to −22·4)
Singapore	938 (883 to 984)	3·9% (3·7 to 4·1)	13·4 (12·7 to 14·1)	−16·4% (−21·3 to −12·1)	−50·9% (−53·3 to −48·6)	−64·9% (−66·8 to −63·1)
Thailand	50 800 (40 200 to 62 700)	8·1% (7·2 to 8·9)	65·9 (52·8 to 80·9)	4·2% (−18·7 to 30·6)	−48·8% (−55·0 to −42·8)	−28·1% (−43·4 to −10·6)
Viet Nam	65 800 (53 100 to 79 300)	9·4% (8·1 to 10·4)	68·5 (54·4 to 81·9)	22·9% (−3·3 to 55·2)	−24·1% (−32·2 to −15·4)	−28·1% (−43·4 to −11·1)

Data n (95% UI), % (95% UI), and rate (95% UI). ASEAN=Association of Southeast Asian Nations. UI=uncertainty interval.

**Table 3:** Number of DALYs (in thousands), proportion of DALYs relative to all-cause DALYs, and age-standardised DALY rates per 100 000 people due to injuries in 2021, along with changes from 1990 to 2021, for ASEAN and its member states

	**DALYs in 2021**			**Change in DALYs from 1990 to 2021**		
	Number of DALYs, in thousands	Percent of all-cause DALYs	Age-standardised DALY rates, per 100 000 people	Percentage change in number of DALYs	Percentage change in percent of all-cause DALYs	Percentage change in age-standardised DALY rates
ASEAN	18 400 (16 700 to 20 200)	8·1% (7·4 to 8·7)	2702·1 (2451·5 to 2964·4)	−9·6% (−17·2 to −0·3)	−21·5% (−28·4 to −14·6)	−40·2% (−45·0 to −34·6)
Brunei	9·75 (8.43 to 11.4)	9·7% (9·1 to 10·4)	2108·2 (1826·2 to 2459·0)	−3·0% (−12·5 to 7·8)	−43·1% (−46·5 to −39·3)	−49·2% (−53·9 to −44·2)
Cambodia	571 (466 to 720)	9·5% (8·4 to 10·6)	3506·2 (2887·8 to 4388·8)	−8·7% (−26·7 to 17·5)	19·5% (3·1 to 37·0)	−40·9% (−52·0 to −26·3)
Indonesia	5770 (5000 to 6720)	6·1% (5·3 to 6·8)	2124·9 (1860·0 to 2456·6)	−15·4% (−27·3 to 0·7)	−24·4% (−35·2 to −14·0)	−42·4% (−50·0 to −32·7)
Laos	222 (175 to 278)	8·5% (7·7 to 9·3)	3028·1 (2414·8 to 3766·6)	−21·0% (−39·5 to 3·8)	25·3% (9·0 to 46·9)	−53·0% (−63·4 to −39·2)
Malaysia	790 (745 to 849)	8·6% (7·9 to 9·3)	2395·8 (2255·4 to 2577·0)	46·7% (38·6 to 57·3)	−28·1% (−32·8 to −22·4)	−27·0% (−31·2 to −21·6)
Myanmar	2000 (1710 to 2360)	9·0% (8·1 to 9·9)	3552·1 (3051·9 to 4192·5)	−27·1% (−40·0 to −9·8)	−1·7% (−14·5 to 14·1)	−45·2% (−54·3 to −32·4)
Philippines	3050 (2680 to 3450)	7·8% (7·2 to 8·4)	2709·3 (2370·5 to 3066·2)	7·0% (−6·1 to 22·0)	−33·6% (−38·8 to −27·9)	−39·0% (−46·3 to −30·7)
Singapore	86·9 (70.3 to 108)	7·9% (7·1 to 9·0)	1280·6 (1059·0 to 1573·2)	5·7% (−5·2 to 17·0)	−35·0% (−39·7 to −30·1)	−50·4% (−54·4 to −46·3)
Thailand	2670 (2210 to 3210)	11·3% (10·2 to 12·3)	3818·3 (3204·8 to 4577·1)	−14·6% (−30·2 to 2·6)	−37·1% (−43·1 to −31·5)	−28·7% (−41·3 to −14·2)
Viet Nam	3260 (2690 to 3860)	11·6% (10·5 to 12·7)	3239·9 (2697·7 to 3798·3)	−1·5% (−19·3 to 20·9)	−17·9% (−26·4 to −8·4)	−34·7% (−46·3 to −20·9)

Data n (95% UI), % (95% UI), and rate (95% UI). ASEAN=Association of Southeast Asian Nations. UI=uncertainty interval. DALYs=disability-adjusted life-years.

Considerable variations exist within the region (Table 1, Table 2, Table 3). The highest number of injury cases and deaths in 2021 was observed in Indonesia, with an estimated 12·3 million incident cases (95% UI 11·5–13·2) and 92 500 deaths (79 000–111 000). Viet Nam followed, with an estimated 6·16 million incident cases (5·83–6·50) and 65 800 deaths (53 100–79 300). The lowest number of injury cases was observed in Brunei, with an estimated 64 000 cases (59 500–68 400) and 122 deaths (109–135). Injuries accounted for a considerable proportion of all-cause disease burden in Viet Nam, where more than 9·4% (8·1–10·4) of all-cause mortality and 11·6% (10·5–12·7) of total DALYs were attributable to injuries. In Thailand, injuries were associated with 8·1% (7·2–8·9) of all-cause mortality and 11·3% (10·2–12·3) of total DALYs. In Cambodia, injuries constituted 7·3% (6·3–8·2) of all-cause mortality, with more than 9% of total DALYs. Although injuries accounted for a relatively small proportion of all-cause mortality in Singapore, at 3·9% (3·7–4·1), the proportion of total DALYs was 7·9% (7·1–9·0), slightly lower than Laos but higher than in the Philippines and Indonesia (see table 3).

**Figure 1:**
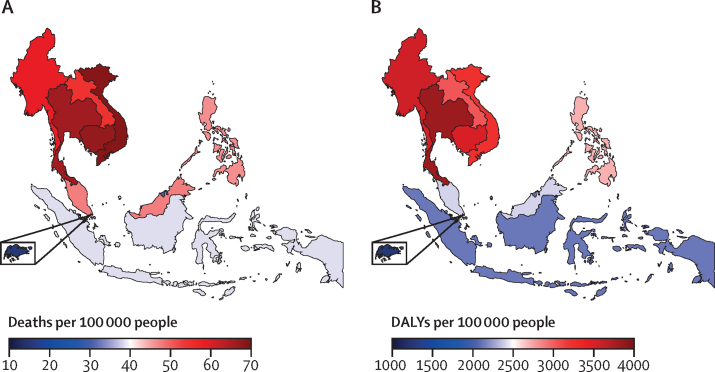
Age-standardised deaths (A) and age-standardised DALYs (B) per 100 000 people attributable to injuries DALY=disability-adjusted life-years.

The age-standardised mortality rate ranged from a low of 13·4 per 100 000 (95% UI 12·7–14·1) in Singapore to a high of 68·5 per 100 000 (54·4–81·9) in Viet Nam (figure 1A; table 2). Cambodia and Thailand both had high age-standardised mortality rates exceeding 65 per 100 000. In terms of DALY rates, the highest age-standardised DALY rate was observed in Thailand, estimated at 3818·3 per 100 000 (3204·8–4577·1), followed by Myanmar with an estimate of 3552·1 per 100 000 (3051·9–4192·5; figure 1B; table 3). Cambodia, Viet Nam, and Laos all had DALY rates higher than 3000 per 100 000. The Philippines, Malaysia, Indonesia, and Brunei had DALY rates between 2000 and 3000 per 100 000. The lowest DALY rate was observed in Singapore, with an estimate of 1280·6 per 100 000 (1059·0–1573·2).

Focusing on level 3 causes, falls were associated with the largest number of incident cases (10 900 000 [95% UI 988 000–12 100 000]) in ASEAN, followed by exposure to mechanical forces (8 110 000 [6 820 000–9 520 000]; appendix p 21). However, in terms of mortality, the largest number of deaths was attributed to road injuries, with an estimated 120 000 deaths (107 000–134 000) recorded in the region, followed by falls with an estimate of 56 500 (45 000–64 000), self-harm with an estimate of 30 200 (25 800–34 300), drowning with an estimate of 29 500 (26 200–33 100), and interpersonal violence with an estimate of 25 000 (22 000–27 900; appendix p 22).

Substantial heterogeneity existed across the region. With regard to incident cases, falls constituted the most incident cases of injuries in eight of the ten ASEAN countries (appendix p 23). In Cambodia, falls accounted for 43·2% of the total injury cases in 2021. Conversely, in Brunei and Singapore, exposure to mechanical forces^23^ was the most common cause of injury, accounting for more than 35% of total injury incident cases in 2021 (appendix pp 24, 40). With respect to mortality and DALYs, road injuries were the leading cause of injury-related mortality (appendix pp 25, 41) and DALYs (appendix pp 26, 42) in all countries except the Philippines, where it ranked second for both metrics, and Singapore, where it ranked third for mortality and fourth for DALYs (appendix p 23). The burden of road injuries was particularly severe in Thailand and Malaysia (appendix pp 25–26, 43–44). In Thailand, the age-standardised mortality rate was 29·7 per 100 000 (95% UI 23·8–36·9), and the age-standardised DALY rate was 1697·3 per 100 000 (1383·3–2077·5). In Malaysia, the age-standardised mortality rate was 23·7 per 100 000 (21·8–25·7), and the age-standardised DALY rates was 1140·1 per 100 000 (1060·5–1224·6). In Thailand, motorcycle accidents (level 4) were the primary cause of road injuries, whereas in Malaysia, motor vehicle accidents were the main contributors to road injuries.

Falls was the second leading cause of injury mortality in Brunei, Cambodia, Indonesia, Myanmar, Singapore, and Viet Nam, and the leading cause of DALYs in Singapore, where they accounted for more than 33% of total injury DALYs in 2021 (appendix pp 23, 25–26, 41–42). Interpersonal violence was the leading cause of injury mortality and DALYs in the Philippines, accounting for 30·4% of total injury deaths and 28·6% of injury-related DALYs. It was also the second leading cause of injury mortality in Laos. Self-harm was the leading cause of injury-related mortality in Singapore, constituting 47·3% of total injury mortality, and it was among the top three leading causes of injury-related mortality in Malaysia, Thailand, Brunei, and Viet Nam. Conflict and terrorism resulted in substantial injury-related mortality and DALYs in Myanmar, where more than 2560 deaths (95% UI 2330–2820) and 201 000 DALYs (169 000–257 000) were attributable to this cause, ranking it as the fourth leading cause of injury burden.

Injury patterns differ by sexes and ages (appendix p 27). Across the region, incidence of injuries was higher among males than females, with an estimated age-standardised rate of 6388·3 per 100 000 (95% UI 6057·0–6727·2) among males and 4076·7 per 100 000 (3791·3–4366·4) among females (appendix pp 45–48). The highest incidence rate in males was observed in the age group of 20–24 years, where the incidence was estimated to be 10 237·5 per 100 000 (9212·6–11 445·0). As for females, a high incidence was observed in the age group of 85 years and older, higher than 12 000 per 100 000. In terms of relative disease burden, injuries accounted for the highest share of total DALYs among males aged 15–19 years, with 35·8% (31·7–40·5) of all-cause DALYs (appendix p 28). In females, the share of injuries-attributable DALYs was substantially smaller and is the highest among children aged 5–9 years compared to other age groups, with an estimated 12·1% (10·3–14·0) of total DALYs.

**Figure 2:**
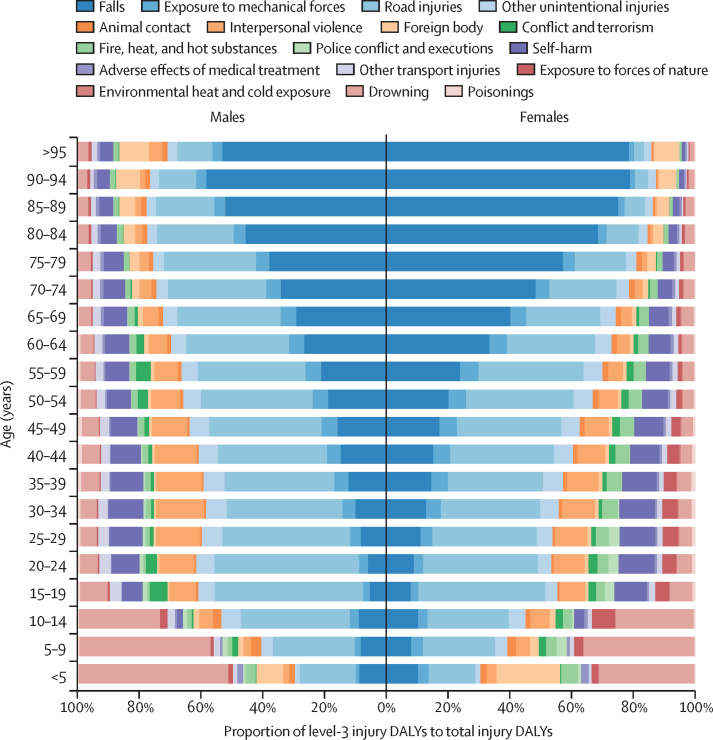
Relative contribution of level 3 injury DALYs to the total injury DALYs by sex and age group in ASEAN, 2021 ASEAN=Association of Southeast Asian Nations. DALY=disability-adjusted life-years.

Road injuries were the primary contributors to DALYs in the younger age groups, whereas falls were the major contributor to higher burden among the older populations in ASEAN (figure 2). In Thailand, Malaysia, Indonesia, and Viet Nam, the highest road-injuries-attributable burden was observed among males aged 15–19 years (appendix p 29). The share of total DALYs caused by road injuries within that demographic group was estimated as follows: 31·2% (95% UI 25·4–36·5) in Thailand, 23·7% (19·0–28·5) in Malaysia, 19·0% (15·5–22·7) in Indonesia, and 19·0% (13·5–25·0) in Viet Nam (appendix p 30). In the other ASEAN countries, the highest road-injuries-attributable burden was observed among males aged 20–24 years, ranging from 16·0% (11·2–21·0) in Cambodia to 5·2% (4·2–6·4) in Singapore. Road injuries burden among females was substantially smaller (appendix p 30). Across the region, the proportion of total DALYs attributable to road injuries among females aged 20–24 years was estimated at 3·6% (2·8–4·5), compared to 16·2% (14·1–18·6) among males in the same age group (appendix p 30).

In Singapore and the Philippines, road injuries were not the leading cause of injury-related disease burden in the younger age group. In Singapore, self-harm accounted for the largest disease burden among males aged 20–24 years (appendix p 29), representing 11·4% (95% UI 9·2–14·2) of total DALYs (appendix p 31). In the Philippines, the highest injury-attributable burden was due to interpersonal violence (appendix p 29), particularly affecting adult males aged 25–29 years, with 10·3% (9·0–11·6) of total attributable DALYs (appendix p 32). Additionally, in Myanmar, males aged 15–19 years were disproportionately affected by conflict and terrorism (appendix p 29), with 13·3% (11·2–15·5) of DALYs attributed to this cause (appendix p 33).

Among children aged 5–9 years, drowning was a common cause of injury-related DALYs in many ASEAN countries (appendix p 29). In Viet Nam, it accounted for 17·1% (95% UI 12·2–22·5) of total DALYs among males and a relatively high proportion among females in this age group, estimated at 10·2% (6·4–14·2; appendix p 34). In Thailand, drowning constituted 11·8% (8·6–15·3) of total DALYs among males in this age group. In Cambodia, the estimate was 10·3% (6·6–15·1), in Laos, 9·7% (5·9–13·9), and in Myanmar, 9·9% (6·4–13·7).

Between 1990 and 2021, the age-standardised incidence rate of injuries across the region decreased by 23·0% (95% UI 21·8–24·1; table 1; appendix p 35). The largest reduction was observed in Laos, with a 39·1% (32·1–48·4) decline between 1990 and 2021. However, Myanmar did not see a significant decline in the incidence rate of injuries over the past 30 years. In terms of disease burden, age-standardised DALY rates associated with injuries has decreased by 40·2% (34·6–45·0) across the region, with reductions exceeding 50% in Laos and Singapore (table 3; appendix p 35).

Drowning saw the steepest decline in age-standardised DALY rates, with a reduction of 60·6% (95% UI 53·2–66·7; appendix pp 49–51). Substantial declines in drowning-related DALY rates were observed among both males and females across all ASEAN member states (appendix p 36). Road injuries saw a decline in age-standardised DALY rates of 39·6% (95% UI 32·1–46·4; appendix pp 49–51). Singapore leads the region in reducing the disease burden associated with road injuries, with a reduction in DALY rates of 78·1% (76·3–79·9). By contrast, in Viet Nam, Thailand, and the Philippines, DALY rates associated with road injuries improved substantially among females, but no significant changes were observed among males (appendix pp 37, 49–51). Progress in falls has been less substantial and more heterogeneous (appendix p 38). Across ASEAN, DALY rates associated with falls have decreased by 12·3% (2·6–21·0) in the past 30 years. In Singapore, although the disease burden associated with falls decreased by 29·6% (26·2–33·7) among males, no changes were seen among females. A lack of improvement in falls-related disease burden was also observed in Laos, Cambodia, Viet Nam, and Myanmar, and among males in the Philippines, Indonesia, and Thailand.

## Discussion

To our knowledge, this is the first comprehensive analysis of injury epidemiology and burden in ASEAN. Injuries remain a major public health challenge, despite some improvements over the past three decades. With an estimated 35·5 million incident cases (95% UI 33·5–37·7), 317 000 deaths (286 000–350 000), and 18·4 million DALYs (16·7–20·2), injuries—especially road injuries and falls—were ranked among the top causes of mortality and morbidity in the region in 2021.^14^ Disparities exist across the region: Indonesia observed the highest number of injury cases and deaths; Viet Nam showed the highest age-standardised mortality rate; and Thailand had the highest rate of injury-related DALYs. Falls were the most common cause of injuries, whereas road injuries were associated with the highest mortality and disease burden in most ASEAN countries. Distinct from the regional trend, in the Philippines, interpersonal violence was the leading cause of injury-related mortality and burden. In Singapore, the leading cause of injury-related mortality was self-harm, which accounted for nearly half of all injury deaths in the country. Consistent with global patterns, males experienced higher injury incidence and burden, especially between the ages of 15 years and 29 years.^24^ Much of the disparity was driven by disproportionate exposure among males to road injuries^25^ and, in some countries, interpersonal violence and conflict and terrorism.^26^, ^27^

On the basis of our findings, five causes of injury warrant particular attention: road injuries; falls; drowning; interpersonal violence; and self-harm. Road injuries remain a central priority for ASEAN. Our results align with WHO 2021 data,^28^ showing persistently high road injury mortality across ASEAN.^28^ Contributing factors to high mortality and DALY rates include inadequate infrastructure, high motorcycle usage, low helmet compliance, drunk driving, and insufficient enforcement of road safety regulations.^29^ In 2016, the ASEAN Secretariat introduced a regional road safety strategy addressing five pillars of road safety—institutional framework, safer roads and mobility, safer vehicles, safer road users, and post-crash response.^29^ Progress has been made in some of the pillars. For example, the ASEAN Highway Network,^30^ launched in 1999, reduced substandard roads by 46·2%,^31^ and road safety audits have been implemented in most ASEAN countries.^32^ The New Car Assessment Program, which has shown substantial injury and fatality reductions in the USA, Australia, and Europe,^33^, ^34^, ^35^ was launched in ASEAN in 2011^36^ to advance vehicle safety standards.^37^ Among the five pillars, road-user safety remains the most challenging, with key gaps including enforcing speed limits, addressing drunk driving, mandating seatbelt use, promoting the use of child restraint systems, and prohibiting mobile device use while driving.^29^ Effective road safety management is hindered by the scarcity of systematic data surveillance on injuries and outcomes in some ASEAN countries,^29^ complicating goal setting, actions, and resource allocation.^38^ As each country continues to upgrade its road traffic infrastructure and safety practice standards, strengthening data collection is essential for monitoring traffic incidents and outcomes.

Beyond the long-standing public health issue of road injuries, falls have emerged as a mounting threat, particularly among older adults. With ASEAN's population older than the age of 60 years projected to double and exceed 22% by 2050,^39^ addressing falls is of imminent importance.^40^ Historically, fall prevention has been overlooked in regional public health initiatives,^40^ and most countries do not have dedicated resources and programmes.^41^ Besides ageing, obesity, sedentary lifestyles, and socioeconomic hardships also increase fall risk.^42^ In the past three decades, ASEAN has experienced one of the fastest rises in obesity prevalence,^43^ while urban physical inactivity remains high.^44^ Additionally, poverty, health inequity, and illiteracy remain pervasive social issues in a number of countries,^45^ and together with suboptimal environmental safety and restricted health-care access, they increase the risk and severity of fall injuries.^46^, ^47^ Fall prevention strategies should, therefore, extend beyond the care of older people, integrating population health and social reform to address both individual and systemic challenges.^48^ WHO's Step Safely plan offers some general evidence-based guidelines for fall prevention across different stages of life.^49^ In addition to promoting active lifestyles and enhancing environmental safety, strengthening fall injury data collection is essential to support monitoring, evaluation, and evidence-based policymaking in fall prevention.

Although falls disproportionately affect older populations, drowning imposes the greatest burden on younger ones. Consistent with the WHO Global Status Report on Drowning Prevention 2024,^50^ our study identified drowning as a crucial public health concern among children and adolescents, particularly in Viet Nam, Thailand, Cambodia, Laos, and Myanmar. Drowning is preventable through a range of strategies, from community-based measures such as restricting water access and water safety skills training to policies enhancing water transportation safety and disaster preparedness.^51^ Examples of water safety interventions in the region include Viet Nam's survival swimming curricula and water safety education programme,^52^ and Thailand's public health campaigns and safety regulations introduced by the Child Drowning Prevention Committee.^53^ However, many countries still do not have comprehensive drowning prevention strategies.^50^ Another challenge is the absence of comprehensive national data, with less than a quarter of ASEAN countries using drowning data for research, thereby limiting policy development.^54^ The socioeconomic burden of drowning is far from trivial. By 2050, drowning could cause 26·3 million deaths or injuries—predominantly among children—resulting in an estimated cost of US$4 trillion globally.^50^ Addressing this crisis requires stronger political commitment and decisive action.

In 2021, self-harm was the leading cause of injury-related deaths in Singapore, with the highest burden among males aged 20–24 years, mirroring trends in other high-income countries.^55^ Factors elevating individuals’ risk of self-harm include mental and neurological disorders, socioeconomic inequalities, poverty, food and water insecurity, alcohol use, and media influence.^56^ In Singapore, mental disorders are a major contributor.^57^ Approaches to self-harm prevention can vary across ASEAN because of sociocultural differences, but two key elements remain. First, destigmatisation and decriminalisation. Progress is underway, with Brunei and Myanmar being the only countries penalising suicide attempts.^58^ Second, enhancing surveillance and improving reporting transparency.^59^ Self-harm data remain largely absent across ASEAN countries, yet such data are essential for advancing research and informing more effective intervention strategies.

Finally, over the past three decades, interpersonal violence has remained a substantial contributor to morbidity and mortality burden in ASEAN, with Myanmar and the Philippines recording the highest death and DALY rates. Much of this violence stems from intrastate conflicts. Myanmar has been plagued by decades of political instability, characterised by ethnic conflicts and military suppression, with the Rohingya crisis in 2017 and the 2021 military coup triggering widespread civil unrest.^60^ In the Philippines, the 2016 war on drugs led to a surge in interpersonal violence.^61^ Despite international criticism, ASEAN has upheld its non-interference principle.^62^ Founded during the Cold War to promote regional stability and cooperation,^63^ it maintains a non-intrusive stance, believing member states can resolve domestic issues independently.^64^ However, this stance has been challenged.^65^ As domestic conflicts persist, ASEAN must navigate the fine line between the responsibility to protect^66^ and its non-interference principle.^67^ The *Lancet* Commission on Peaceful Societies through Health Equity and Gender Equality^68^ advocates for health equity and gender equality as long-term peacekeeping strategies. Achieving these formidable endeavours requires strengthening local capacity for community-led initiatives and ensuring political accountability. For a region as socially, culturally, and ethnically diverse as ASEAN, such recommendations seem eminently relevant.

This study is subject to several limitations, including those described in previous GBD publications.^1^, ^17^ First, the availability of data affects accurate estimation. Several ASEAN countries lack comprehensive health and injuries surveillance, as well as vital registration data.^69^ The literature data included in the analysis are also subject to publication bias, with some countries contributing fewer datapoints. In circumstances in which data were scarce, estimates of various metrics were driven by statistical models that rely on covariates and spatiotemporal associations to interpolate missing values. Despite our best efforts to validate and ensure the empirical rigour of our process, large uncertainty remained in some estimates. To enhance the monitoring of injury incidence and burden in ASEAN and support future research, the establishment of a regional injury database, similar to the European Injury Database,^70^ which provides real-time injury data, could be considered. Second, the impact of COVID-19 on injury incidence and burden might not be fully realised because of limitations in data availability and quality during the pandemic. Hospital data from other countries have shown a reduction in occupational and traffic injuries.^71^, ^72^ Our findings indicated a noticeable decline in mortality caused by road injuries in Malaysia and the Philippines in 2020 compared with 2019, followed by a resurgence in 2021 (appendix p 39). However, changes are less apparent among other countries. It has been suggested that COVID-19 was linked to increased self-harm.^73^ Trends in self-harm are increasing steadily in several countries in the region; our data did not indicate any abrupt changes during 2020 and 2021, although a delayed effect of risk factor triggers could be possible. Third, for certain causes of injury, our case definition could have led to an underestimation of the actual incidence. For example, we only considered falls that require medical attention. However, not all falls result in injuries that necessitate medical care, and health-care-seeking behaviour varies among individuals. As a result, the incidence presented probably omits some of the less severe cases of falls. Fourth, for some causes of injuries, such as sexual violence and self-harm, under-reporting is common.^74^ Conversely, for conflict and terrorism, disrupted infrastructure and governmental operation restrict accurate accounting for incidence and mortality. The abrupt surges in incidence and mortality during turmoil deviate from normal epidemiological trajectories, posing challenges to modelling efforts. Fifth, the results presented in this study reflect national level data; however, the burden and risk of injuries vary considerably by socioeconomic status, physical environment, and health-care accessibility. To enhance the localisation and contextualisation of interventions, future research should further investigate subnational and subpopulation disease burdens. Finally, in this study, we focused on the descriptive epidemiology and burden of injuries without in-depth examination of the effects of policy measures. Future research could leverage GBD data to evaluate population-level injury intervention programmes and generate evidence to inform ongoing policy reforms.

The ASEAN Post-2015 Health Development Agenda designated injury prevention as a health priority, albeit with a somewhat narrow focus on road injuries. As highlighted in this study, the leading causes of injuries affecting ASEAN countries vary broadly by geography and demographics, ranging from self-harm in Singapore to interpersonal violence in the Philippines, and from drowning in children to falls among older adults. Using the best-available data to date, our study is the first that examined the injury-related burden in ASEAN as a region. As member states prepare for the ASEAN post-2025 health agenda, there is an opportunity to revisit the scope of injury prevention and devise a holistic, whole-government approach that integrates national and regional public health strategies ensuring a comprehensive and sustainable response to injury. Such a framework could serve as a blueprint for tailored local implementation. The translation of research into policy is rarely a linear process.^75^ The findings presented here, along with the identified challenges and limitations, aim to encourage close examination of crucial injury-related issues, stimulate deep dialogue, and inspire further multisectoral collaboration. Effectively addressing injuries will foster a safer and healthier ASEAN, thereby securing sustainable and equitable long-term development for the region.

### Collaborators

### Affiliations

### Contributors

### Data sharing

To download the input data used in these analyses, please visit the Global Health Data Exchange GBD 2021 website (https://ghdx.healthdata.org/gbd-2021/sources). All results from this study are publicly accessible. To download estimates produced in these analyses, please visit the GBD results tool (https://vizhub.healthdata.org/gbd-results/).

## Declarations of interests

QESA reports grants or contracts from Online Library Data Research funds from Universitas Padjadjaran, Bandung, Indonesia, under contract 2152/UN6.3.1/PT.00/2024 and Acceleration to Associate Professor Grant from Universitas Padjadjaran, Bandung, Indonesia, under contract 1592/UN6.3.1/PT.00/2024, all outside the submitted work. NEI reports leadership or fiduciary roles in other board, society, committee, or advocacy groups, unpaid, as Bursar and Council Member of the Malaysian Academy of Pharmacy, and as a Committee Member of the Education Chapter, Malaysian Pharmacists Society, outside the submitted work. RDGJ reports payment or honoraria for lectures, presentations, speakers bureaus, manuscript writing, or educational events from the International Parkinson's and Movement Disorder Society, Royal Care Hospital, Taiwan Medical University, and HI-Eisai, and support for attending meetings or travel from Torrent Pharma, Innogen, AbbVie, Medtronic, Sun, Natrapharm, and OnePharma, all outside the submitted work. VCFP reports grants or contracts from Sanofi Consumer Healthcare to conduct research on self-care in the Philippines, and payment or honoraria for manuscript writing from the Zuellig Family Foundation to write papers on health systems strengthening, all outside the submitted work. YLS reports grants or contracts from Institute of Epidemiology and Preventive Medicine, National Taiwan University through their post-doctoral fellow contract, leadership or fiduciary role in other board, society, committee or advocacy group, paid or unpaid, as co-founder of the Benang Merah Research Center (benangmerah.net), and other financial or non-financial interests in Jago Beasiswa (idebeasiswa.com) as a mentor, all outside the submitted work. JHVT reports leadership or fiduciary role in other board, society, committee or advocacy group, paid or unpaid, as co-founder of the Benang Merah Research Center (benangmerah.net), outside the submitted work. All other authors report no competing interests.
