# Supplementary material for: The epidemiology and burden of injury in countries of the Association of Southeast Asian Nations (ASEAN), 1990–2021: findings from the Global Burden of Disease Study 2021
Source: Lancet Public Health. 2025 May 27;10(6):e456–66. doi: 10.1016/S2468-2667(25)00069-6 (PMC12127264; doi:10.1016/S2468-2667(25)00069-6)
Supplement: Supplementary appendix [file mmc1.pdf]

THE LANCET
Public Health

Supplementary appendix

This appendix formed part of the original submission and has been peer reviewed. We post it as supplied by the authors.

Supplement to: GBD 2021 ASEAN Injuries Collaborators. The epidemiology and burden of injury in countries of the Association of Southeast Asian Nations (ASEAN), 1990–2021: findings from the Global Burden of Disease Study 2021. *Lancet Public Health* 2025; **10:** e456–66.

Appendix

Supplement to: The epidemiology and burden of injury in countries of the Association of Southeast Asian Nations (ASEAN), 1990-2021: A systematic analysis of the Global Burden of Disease Study 2021

Table of Contents

Appendix 1
Search terms used in Research in Context 3
Supplementary methods 4
GBD cause hierarchy for injuries 4
Case definition of levels 1, 2, and 3 causes of injury 5
Data Sources 8
Statistical analysis 17
GATHER Checklist 19
Supplemental results 21
Authors' affiliations 52
Authors' contributions 54

Search terms used in Research in Context

Table S1 Search terms used in PUBMED literature search to identify existing evidence on ASEAN burden of injury

No	Search terms	Results
1	"wounds and injuries" / or burns/ or crush injuries/ or “transport injuries”/ or “traffic injuries”/ or “road injuries”/ or drowning/ or electric injuries/ or foreign bodies/ or fractures, bone/ or frostbite/ or unintentional injuries/	246,613
2	accident prevention/ or accidental falls/ or accident, home/ or accident, traffic/ or drowning/	169,993
3	self-injurious behavior or self-mutilation or self-harm* or self harm* or suicid* or self-inflicted injuries	137,282
4	“occupational injuries”	112
5	"Global Burden of Disease"	7,204
6	estimate	1,540,138
7	burden	394,504
8	ASEAN or Brunei Darussalam or Cambodia or Indonesia or Laos or Lao PDR or Malaysia or Myanmar or Philippines or Singapore or Thailand or Vietnam	474,792
9	1 or 2 or 3 or 4	537,991
10	5 or 6 or 7	1,762,543
11	8 and 9 and 10	746

* indicates a truncated word

Supplementary methods

GBD cause hierarchy for injuries

Table S2 GBD Cause Hierarchy specific to injuries

Level 1	Level 2	Level 3	Level 4
**Injuries**	Transport injuries	Road injuries	Pedestrian road injuries
			Cyclist road injuries
			Motorcyclist road injuries
			Motor vehicle road injuries
			Other road injuries
		Other transport injuries	
	Unintentional injuries	Falls	
		Drowning	
		Fire, heat, and hot substances	
		Poisonings	Poisoning by carbon monoxide
			Poisoning by other means
		Exposure to mechanical forces	Unintentional firearm injuries
			Other exposure to mechanical forces
		Adverse effects of medical treatment	
		Animal contact	Venomous animal contact
			Non-venomous animal contact
		Foreign body	Pulmonary aspiration and foreign body in airway
			Foreign body in eyes
			Foreign body in other body part
		Environmental heat and cold exposure	
		Exposure to forces of nature	
		Other unintentional injuries	
	Self-harm and interpersonal violence	Self-harm	Self-harm by firearm
			Self-harm by other specified means
		Interpersonal violence	Physical violence by firearm
			Physical violence by sharp object
			Sexual violence
			Physical violence by other means
		Conflict and terrorism	
		Police conflict and executions	

Case definition of levels 1, 2, and 3 causes of injury

Table S3 Abbreviated definition of causes of injuries

Cause Name	Abbreviated definition
Injuries	GBD defines injuries as death or disability due to the direct or indirect result of a physical force, immersion, or exposure, including accidental, interpersonal, or self-inflicted forces as well as war, conflict, violence, and natural disasters.
Transport injuries	Death or disability resulting from all subtypes of road injuries, including non-road transport injuries. ICD-9: E800-E807, E830E838, E840-E849; ICD-10: V00-V86.9, V87.2-V87.3, V88.2-V88.3, V90-V98.8.
Road injuries	Road injuries includes death or disability due to unintentional interaction with an automobile, motorcycle, pedal cycle, or other vehicles. ICD-10: V01-V04.99, V06-V80.929, V82-V82.9, V87.2-V87.3; ICD-9: comparable codes from the E800 chapter.
Other transport injuries	Death or disability due to injury sustained from transport other than automobile, motorcycle, pedal cycle, or other road vehicles. ICD-10: V00-V00.898, V05-V05.99, V81-V81.9, V83-V86.99, V88.2-V88.3, V90-V98.8; ICD-9: comparable E800 and E900 chapter codes.
Unintentional injuries	Death or bodily damage due to an injury that was unplanned or caused by external factors.
Falls	Falls includes death or disability resulting from a sudden movement downward due to slipping, tripping, or other unintentional movement that results in a person coming to rest at a lower level or against an object. Included are ICD-9: E880–E886.99, E888E888.9, E929.3; ICD-10: W00–W19.9.
Drowning	Drowning includes deaths and disability associated with unintentional immersion in water or another fluid. Cases due to water transport or natural disasters are assigned elsewhere. ICD-9: E910-E910.99; ICD-10: W65-W70.9, W73-W74.9.
Fire, heat, and hot substances	Death or disability due to unintentional exposure to substances of high temperature, including hot liquid, solid, or gas such as cooking stoves, smoke, steam, drinks, machinery, appliances, tools, radiators, and objects radiating heat energy. Included are ICD-9: E890-E899.09, E924-E924.99, E929.4; ICD-10: X00-X06.9, X08-X19.9.
Poisonings	Unintentional exposure to a non-infectious substance which contacts or enters the body via inhalation, ingestion, injection, or absorption, and causes deranged physiological function or death. ICD-9: E856-E857.99, E860-E865, E867-E869.99, E929.2; ICD-10: J70.5, X46-X48.9.
Exposure to mechanical forces	Unintentional death or disability resulting from contact with or threat of an animate or inanimate object, human, or plant.

	This category includes unintentional firearm injuries as well as other miscellaneous forces (eg, power tools or household machinery). ICD-9: E916E922.99, E928.1-E928.7; ICD-10: W20-W38.9, W40-W43.9, W45.0-W45.2, W46.0-W46.2, W49-W52.
Adverse effects of medical treatment	Death or short-term or long-term disability sustained as the result of undergoing a procedure, treatment, or other exposure to the health-care system. This exposure can occur in inpatient admission, outpatient facilities, emergency care, or during home treatment.
Animal contact	Animal contact includes deaths and injuries resulting from unintentionally being attacked, struck, impaled, bitten, stung, crushed, exposed to, or stepped on by a non-human animal. Included are ICD-9: E905–E906.99, ICD-10: W52.0–W62.9, W64–W64.9, X20–X29.9.
Foreign body	Unintentional death or bodily damage from an extraneous material or substance being within the body, including the airway, lungs, nose, and eyes. ICD-9: 360.5-360.69, 374.86, 376.6, 709.4, 770.1-770.18, E911-E912.09, E913.0-E913.19, E913.8-E913.99, E914-E914.09, E915E915.09; ICD-10: H02.81-H02.819, H44.6-H44.799, M60.2-M60.28, W44-W45, W45.3-W45.9, W75-W76.9, W78-W80.9, W83-84.9.
Environmental heat and cold exposure	Deaths or disability resulting from exposure to high or low temperatures in the environment, not including contact with fire, heat, or hot substances. ICD-9: E900-E902.99, E926-E926.99, E929.5; ICD-10: L55-L55.9, L58-L58.9, W88-W99.9, X30-X32.9, X39-X39.9.
Exposure to forces of nature	Death or bodily harm resulting from an unforeseen and often sudden natural event such as a hurricane, earthquake, tsunami, tornado, lightning strike, volcanic eruption, avalanche, or flood. ICD9: E907-E909.9, ICD10: X33-X38.9 .
Other unintentional injuries	Death or disability resulting from unintentional injuries not included in transport injuries or elsewhere in unintentional injuries. ICD-9: E903-E904.99, E913.2-E913.39, E923-E923.99, E925-E925.99, E927-E928.09, E928.8-E928.89; ICD-10: W39-W39.9, W77-W77.9, W81W81.9, W85-W87.9, X50-X58.9.
Self-harm and interpersonal violence	This cause includes death or disability from deliberate bodily damage inflicted on oneself or from intentional use of physical force or power from another person or group not including military or police forces. ICD-9: E950–E969, ICD-10: X60–X64.9, X66–Y08.9, Y87.0Y87.2.
Self-harm	Self-harm is deliberate bodily damage inflicted on oneself resulting in death or injury. ICD-9: E950-E959; ICD-10: X60-X64.9, X66-X84.9, Y87.0.
Interpersonal violence	GBD defines interpersonal violence as death or disability from intentional use of physical force or power from another

	person or group not including military or police forces. Included are ICD-9: E960–E969; ICD-10: X85–Y08.9, Y87.1–Y87.2.
Conflict and terrorism	Death or bodily harm resulting from the instrumental use of violence by people who identify themselves as members of a group—whether this group is transitory or has a more permanent identity—against another group or set of individuals, in order to achieve political, economic, or social objectives. ICD-9: E979-E979.9, E990-E999.1; ICD-10: U00-U03, Y36-Y38.9, Y89.1.
Police conflict and executions	State-sanctioned executions or police-related altercations leading to death or bodily harm. ICD-9: E970-E978; ICD-10: Y35Y35.93, Y89.0.

Source: https://www.healthdata.org/research-analysis/diseases-injuries-risks/factsheets-hierarchy

Data Sources

A summary of all the data sources used for causes of death and non-fatal outcomes are presented in SM Table .

Table S4 Causes of death data sources used in estimation of mortality and YLL of injuries in the ten ASEAN countries

Citation	Country	Year
Climate Change and African Political Stability Project (CCAPS). Armed Conflict Location and Event Dataset, Realtime - Robert S. Strauss Center as referenced in Raleigh, Clionadh, Andrew Linke, Havard Hegre and Joakim Karlsen. 2010. Introducing ACLED-Armed Conflict Location and Event Data. Journal of Peace Research 47(5), 651-60.	Brunei	2010
Peace Research Institute Oslo (PRIO). Battle Deaths Dataset Version 3.1, 2009. Oslo, Norway: Peace Research Institute Oslo (PRIO), 2009.	Brunei	2009
Brunei Darussalam Vital Registration - Deaths 2017-2018 ICD10. as it appears in WHO Mortality Database Version December 2019	Brunei	2017-2018
Brunei Vital Registration - Deaths 1996-2010 ICD10.	Brunei	1996-2010
Brunei Vital Registration - Deaths 2011-2015 ICD10. as it appears in WHO Mortality Database Version October 2017	Brunei	2011-2015
Brunei Vital Registration - Deaths 2016 ICD10. as it appears in WHO Mortality Database Version December 2018	Brunei	2016
World Health Organization (WHO). Global Status Report on Road Safety 2009. Geneva, Switzerland: World Health Organization (WHO), 2009.	Brunei	2009
National Consortium for the Study of Terrorism and Responses to Terrorism (START). Global Terrorism Database. College Park , MD, United States of America: University of Maryland.	Brunei	NA
United Nations Office on Drugs and Crime (UNODC). United Nations Office on Drugs and Crime Global Study on Homicide 2011. Vienna, Austria: United Nations Office on Drugs and Crime (UNODC), 2011.	Brunei	2011
Climate Change and African Political Stability Project (CCAPS). Armed Conflict Location and Event Dataset, Realtime - Robert S. Strauss Center as referenced in Raleigh, Clionadh, Andrew Linke, Havard Hegre and Joakim Karlsen. 2010. Introducing ACLED-Armed Conflict Location and Event Data. Journal of Peace Research 47(5), 651-60.	Cambodia	2010
Peace Research Institute Oslo (PRIO). Battle Deaths Dataset Version 3.1, 2009. Oslo, Norway: Peace Research Institute Oslo (PRIO), 2009.	Cambodia	2009
National Institute of Statistics (Cambodia), Statistics Sweden. Cambodia Socio-Economic Survey 2003-2005. Phnom Penh, Cambodia: National Institute of Statistics (Cambodia).	Cambodia	2003-2005
Centre for Research on the Epidemiology of Disasters (CRED). EM-DAT: The OFDA/CRED International Disaster Database. Brussels, Belgium: Catholic University of Leuven.	Cambodia	NA
National Consortium for the Study of Terrorism and Responses to Terrorism (START). Global Terrorism Database. College Park , MD, United States of America: University of Maryland.	Cambodia	NA

International Institute for Strategic Studies. International Institute for Strategic Studies Armed Conflict Database. London, United Kingdom: International Institute for Strategic Studies.	Cambodia	NA
International Organization for Migration (IOM). Missing Migrants Project 2014-2020. Geneva, Switzerland: International Organization for Migration (IOM).	Cambodia	2014-2020
Goyet S, Rammaert B, McCarron M, Khieu V, Fournier I, Kitsutani P, Ly S, Mounts A, Letson WG, Buchy P, Vong S. Mortality in Cambodia An 18-Month Prospective Community-based Surveillance of All-age Deaths Using Verbal Autopsies. Asia Pac J Public Health. 2013; 1010539513.	Cambodia	2013
Rummel RJ. Statistics Of Democide: Genocide and Mass Murder Since 1900. Charlottesville, Virginia: Center for National Security Law, University of Virginia, 1997.	Cambodia	1900-
Department of Peace and Conflict Research, Uppsala University. UCDP Georeferenced Event Dataset, Version 17.1, 2016. Uppsala, Sweden: Department of Peace and Conflict Research, Uppsala University, 2017.	Cambodia	2016
United Nations Office on Drugs and Crime (UNODC). United Nations Office on Drugs and Crime Global Study on Homicide 2011. Vienna, Austria: United Nations Office on Drugs and Crime (UNODC), 2011.	Cambodia	2011
Wikipedia. 2015 Mina Stampede. San Francisco, United States: Wikipedia; [updated 2016].	Indonesia	2015
INDEPTH. Africa, Asia, Oceania - INDEPTH Network Cause-Specific Mortality - Release 2014. Accra, Ghana: INDEPTH, 2014.	Indonesia	2014
Climate Change and African Political Stability Project (CCAPS). Armed Conflict Location and Event Dataset, Realtime - Robert S. Strauss Center as referenced in Raleigh, Clionadh, Andrew Linke, Havard Hegre and Joakim Karlsen. 2010. Introducing ACLED-Armed Conflict Location and Event Data. Journal of Peace Research 47(5), 651-60.	Indonesia	2010
Peace Research Institute Oslo (PRIO). Battle Deaths Dataset Version 3.1, 2009. Oslo, Norway: Peace Research Institute Oslo (PRIO), 2009.	Indonesia	2009
Department of Political Science, University of Chicago. Chicago Project on Security and Threats (CPOST). 2020. Database on Suicide Attacks (October 02, 2020 Release). [Data File].	Indonesia	2020
Amnesty International. Death Sentences and Executions 2007-2008, 2013. London, United Kingdom: Amnesty International, 2008.	Indonesia	2007-2008, 2013
Wahab A, Choiriyyah I, Wilopo SA. Determining the Cause of Death: Mortality Surveillance Using Verbal Autopsy in Indonesia. Am J Trop Med Hyg. 2017; 97(5): 1461–8.	Indonesia	2017
Centre for Research on the Epidemiology of Disasters (CRED). EM-DAT: The OFDA/CRED International Disaster Database. Brussels, Belgium: Catholic University of Leuven.	Indonesia	NA
National Consortium for the Study of Terrorism and Responses to Terrorism (START). Global Terrorism Database. College Park , MD, United States of America: University of Maryland.	Indonesia	NA
Agency of Health Research and Development (Indonesia). Indonesia Basic Health Research 2007-2008.	Indonesia	2007-2008

Ministry of Health (Indonesia). Indonesia Cause of Death Survey 2010-2011.	Indonesia	2010-2011
Agency of Health Research and Development (Indonesia). Indonesia Mortality Registration System Strengthening Project.	Indonesia	
Statistics Indonesia. Indonesia National Socioeconomic Survey 2004,2007.	Indonesia	2004,2007
Ministry of Health (Indonesia). Indonesia Sample Registration System - Deaths 2012-2014	Indonesia	2012-2014
Agency of Health Research and Development (Indonesia). Indonesia Sample Registration System - Deaths 2015.	Indonesia	2015
International Institute for Strategic Studies. International Institute for Strategic Studies Armed Conflict Database. London, United Kingdom: International Institute for Strategic Studies.	Indonesia	NA
International Organization for Migration (IOM). Missing Migrants Project 2014-2020. Geneva, Switzerland: International Organization for Migration (IOM).	Indonesia	2014-2020
Putri R, Paddock RC. Out of Sixth Grade, and Straight to the Factory She Died In. New York Times [Internet]. 2017 Oct 30; Asia Pacific.	Indonesia	2017
Department of Peace and Conflict Research, Uppsala University. UCDP Georeferenced Event Dataset, Version 17.1, 2016. Uppsala, Sweden: Department of Peace and Conflict Research, Uppsala University, 2017.	Indonesia	2016
Climate Change and African Political Stability Project (CCAPS). Armed Conflict Location and Event Dataset, Realtime - Robert S. Strauss Center as referenced in Raleigh, Clionadh, Andrew Linke, Havard Hegre and Joakim Karlsen. 2010. Introducing ACLED-Armed Conflict Location and Event Data. Journal of Peace Research 47(5), 651-60.	Laos	2010
Peace Research Institute Oslo (PRIO). Battle Deaths Dataset Version 3.1, 2009. Oslo, Norway: Peace Research Institute Oslo (PRIO), 2009.	Laos	2009
Amnesty International. Death Sentences and Executions 2015. London, United Kingdom: Amnesty International, 2016.	Laos	2015
Centre for Research on the Epidemiology of Disasters (CRED). EM-DAT: The OFDA/CRED International Disaster Database. Brussels, Belgium: Catholic University of Leuven.	Laos	NA
National Consortium for the Study of Terrorism and Responses to Terrorism (START). Global Terrorism Database. College Park , MD, United States of America: University of Maryland.	Laos	NA
International Institute for Strategic Studies. International Institute for Strategic Studies Armed Conflict Database. London, United Kingdom: International Institute for Strategic Studies.	Laos	NA
International Organization for Migration (IOM). Missing Migrants Project 2014-2020. Geneva, Switzerland: International Organization for Migration (IOM).	Laos	2014-2020
Department of Peace and Conflict Research, Uppsala University. UCDP Georeferenced Event Dataset, Version 17.1, 2016. Uppsala, Sweden: Department of Peace and Conflict Research, Uppsala University, 2017.	Laos	2016

United Nations Office on Drugs and Crime (UNODC). United Nations Office on Drugs and Crime Global Study on Homicide 2011. Vienna, Austria: United Nations Office on Drugs and Crime (UNODC), 2011.	Laos	2011
Climate Change and African Political Stability Project (CCAPS). Armed Conflict Location and Event Dataset, Realtime - Robert S. Strauss Center as referenced in Raleigh, Clionadh, Andrew Linke, Havard Hegre and Joakim Karlsen. 2010. Introducing ACLED-Armed Conflict Location and Event Data. Journal of Peace Research 47(5), 651-60.	Malaysia	2010
Peace Research Institute Oslo (PRIO). Battle Deaths Dataset Version 3.1, 2009. Oslo, Norway: Peace Research Institute Oslo (PRIO), 2009.	Malaysia	2009
Amnesty International. Death Sentences and Executions 2008, 2010, 2013, 2015-2017. London, United Kingdom: Amnesty International, 2009.	Malaysia	2008, 2010, 2013, 2015-2017
Centre for Research on the Epidemiology of Disasters (CRED). EM-DAT: The OFDA/CRED International Disaster Database. Brussels, Belgium: Catholic University of Leuven.	Malaysia	NA
National Consortium for the Study of Terrorism and Responses to Terrorism (START). Global Terrorism Database. College Park , MD, United States of America: University of Maryland.	Malaysia	NA
International Institute for Strategic Studies. International Institute for Strategic Studies Armed Conflict Database. London, United Kingdom: International Institute for Strategic Studies.	Malaysia	NA
Department of Statistics (Malaysia). Vital Statistics: Peninsular Malaysia 1980-1982. Kuala Lumpur, Malaysia: Department of Statistics (Malaysia), 1983.	Malaysia	1980-1982
Malaysia Vital Registration - Deaths 1997 ICD9.	Malaysia	1997
Malaysia Vital Registration - Deaths 2000-2014 ICD10. as it appears in WHO Mortality Database Version October 2017	Malaysia	2000-2014
World Health Organization (WHO). Malaysia World Health Survey 2003. Geneva, Switzerland: World Health Organization (WHO), 2005.	Malaysia	2003
International Organization for Migration (IOM). Missing Migrants Project 2014-2020. Geneva, Switzerland: International Organization for Migration (IOM).	Malaysia	2014-2020
Department of Peace and Conflict Research, Uppsala University. UCDP Georeferenced Event Dataset, Version 17.1, 2016. Uppsala, Sweden: Department of Peace and Conflict Research, Uppsala University, 2017.	Malaysia	2016
United Nations Office on Drugs and Crime (UNODC). United Nations Office on Drugs and Crime Global Study on Homicide 2011. Vienna, Austria: United Nations Office on Drugs and Crime (UNODC), 2011.	Malaysia	2011
United Nations Office on Drugs and Crime (UNODC). United Nations Surveys on Crime Trends and the Operations of Criminal Justice Systems 1970-2006 as provided by Kavi Bhalla.	Malaysia	1970-2006
Climate Change and African Political Stability Project (CCAPS). Armed Conflict Location and Event Dataset, Realtime - Robert S. Strauss Center as referenced in Raleigh, Clionadh, Andrew Linke, Havard Hegre and Joakim Karlsen. 2010. Introducing ACLED-Armed Conflict Location and Event Data. Journal of Peace Research 47(5), 651-60.	Myanmar	2010

Peace Research Institute Oslo (PRIO). Battle Deaths Dataset Version 3.1, 2009. Oslo, Norway: Peace Research Institute Oslo (PRIO), 2009.	Myanmar	2009
Myint, S, Ministry of Health (Myanmar). Cause of Death Verification Study in Myanmar. Presentation at: World Health Organization Regional Office for South East Asia. Regional Consultation on Mortality Statistics; 2007; New Delhi, India.	Myanmar	2007
Bloomberg Philanthropies, Central Statistical Office (Myanmar), Melbourne School of Population and Global Health, University of Melbourne, Ministry of Health (Myanmar). Causes of death in Myanmar using verbal autopsies 2017-2018 [Unpublished data].	Myanmar	2017-2018
Centre for Research on the Epidemiology of Disasters (CRED). EM-DAT: The OFDA/CRED International Disaster Database. Brussels, Belgium: Catholic University of Leuven.	Myanmar	NA
National Consortium for the Study of Terrorism and Responses to Terrorism (START). Global Terrorism Database. College Park , MD, United States of America: University of Maryland.	Myanmar	NA
International Organization for Migration (IOM). Missing Migrants Project 2014-2020. Geneva, Switzerland: International Organization for Migration (IOM).	Myanmar	2014-2020
Ministry of Health (Myanmar). Myanmar National Mortality Survey 2016.	Myanmar	2016
Doctors Without Borders (MSF). Myanmar/Bangladesh: MSF surveys estimate that at least 6,700 Rohingya were killed during the attacks in Myanmar. Doctors Without Borders (MSF) [press release]. 2017 Dec 12.	Myanmar	2017
Department of Peace and Conflict Research, Uppsala University. UCDP Georeferenced Event Dataset, Version 17.1, 2016. Uppsala, Sweden: Department of Peace and Conflict Research, Uppsala University, 2017.	Myanmar	2017
United Nations Office on Drugs and Crime (UNODC). United Nations Office on Drugs and Crime Global Study on Homicide 2011. Vienna, Austria: United Nations Office on Drugs and Crime (UNODC), 2011.	Myanmar	2011
Climate Change and African Political Stability Project (CCAPS). Armed Conflict Location and Event Dataset, Realtime - Robert S. Strauss Center as referenced in Raleigh, Clionadh, Andrew Linke, Havard Hegre and Joakim Karlsen. 2010. Introducing ACLED-Armed Conflict Location and Event Data. Journal of Peace Research 47(5), 651-60.	Philippines	2010
Peace Research Institute Oslo (PRIO). Battle Deaths Dataset Version 3.1, 2009. Oslo, Norway: Peace Research Institute Oslo (PRIO), 2009.	Philippines	2009
Centre for Research on the Epidemiology of Disasters (CRED). EM-DAT: The OFDA/CRED International Disaster Database. Brussels, Belgium: Catholic University of Leuven.	Philippines	NA
National Disaster Risk Reduction and Management Council (Philippines). Final Report on the Effects of Typhoon Yolanda (Haiyan). 2014.	Philippines	2014
National Disaster Risk Reduction and Management Council (Philippines). Final Report on Tropical Storm Ketsana (Ondoy) and Typhoon Parma (Pepeng). 2009.	Philippines	2009

National Consortium for the Study of Terrorism and Responses to Terrorism (START). Global Terrorism Database. College Park , MD, United States of America: University of Maryland.	Philippines	NA
Calupitan J, Favila A. Hope fades in Philippines for dozens buried in landslides. Associated Press [Internet]. 2018 Sep 17.	Philippines	2018
Shukla, A. Philippines landslide death toll rises to 85 as rescue workers search for 20 missing people. The Independent [Internet]. 2019 Jan 2; Asia.	Philippines	2019
National Statistics Office (Philippines). Philippines Vital Registration - Deaths 2006-2012.	Philippines	2006-2012
Philippines Statistics Authority. Philippines Vital Registration - Deaths 2013-2018.	Philippines	2013-2018
National Statistics Office (Philippines). Philippines Vital Statistics Report 1999-2005. Manila, Philippines: National Statistics Office (Philippines).	Philippines	1999-2005
Department of Peace and Conflict Research, Uppsala University. UCDP Georeferenced Event Dataset, Version 17.1, 2016. Uppsala, Sweden: Department of Peace and Conflict Research, Uppsala University, 2017.	Philippines	2016
World Health Organization (WHO). Global Status Report on Road Safety 2009. Geneva, Switzerland: World Health Organization (WHO), 2009.	Singapore	2009
National Consortium for the Study of Terrorism and Responses to Terrorism (START). Global Terrorism Database. College Park , MD, United States of America: University of Maryland.	Singapore	NA
Ministry of Health (Singapore). Singapore Causes of Death 1980-2017.	Singapore	1980-2017
World Health Organization (WHO). Singapore Vital Registration - Deaths 2019 ICD10. as it appears in WHO Mortality Database Version March 2021	Singapore	2019
Reuters. 20 killed on temple trip in Thailand as bus, train collide. Cable News Network (CNN) [Internet]. 2020 Oct 11.	Thailand	2020
Climate Change and African Political Stability Project (CCAPS). Armed Conflict Location and Event Dataset, Realtime - Robert S. Strauss Center as referenced in Raleigh, Clionadh, Andrew Linke, Havard Hegre and Joakim Karlsen. 2010. Introducing ACLED-Armed Conflict Location and Event Data. Journal of Peace Research 47(5), 651-60.	Thailand	2010
Peace Research Institute Oslo (PRIO). Battle Deaths Dataset Version 3.1, 2009. Oslo, Norway: Peace Research Institute Oslo (PRIO), 2009.	Thailand	2009
Amnesty International. Death Sentences and Executions 2009. London, United Kingdom: Amnesty International, 2010.	Thailand	2009
Centre for Research on the Epidemiology of Disasters (CRED). EM-DAT: The OFDA/CRED International Disaster Database. Brussels, Belgium: Catholic University of Leuven.	Thailand	NA
World Health Organization (WHO). Global Status Report on Road Safety 2009. Geneva, Switzerland: World Health Organization (WHO), 2009.	Thailand	2009
National Consortium for the Study of Terrorism and Responses to Terrorism (START). Global Terrorism Database. College Park , MD, United States of America: University of Maryland.	Thailand	NA

Ministry of Public Health (Thailand). Thailand Burden of Disease and Injuries 1998-1999.	Thailand	1998-1999
Porapakkham Y, Rao C, Pattaraarchachai J, Polprasert W, Vos T, Adair T, Lopez AD. Estimated causes of death in Thailand, 2005: implications for health policy. Popul Health Metr. 2010; 8:14.	Thailand	2005
Thailand Vital Registration - Deaths 1983,1985,1990 ICD9.	Thailand	1983,1985,1990
Thailand Vital Registration - Deaths 1996-1999, 2004, 2009-2010 ICD10.	Thailand	1996-1999, 2004, 2009-2010
Thailand Vital Registration - Deaths 2011-2015 ICD10. as it appears in WHO Mortality Database Version October 2017	Thailand	2011-2015
Thailand Vital Registration - Deaths 2016 ICD10. as it appears in WHO Mortality Database Version April 2018	Thailand	2016
Thailand Vital Registration - Deaths 2017 ICD10. as it appears in WHO Mortality Database Version December 2019	Thailand	2017
Thailand Vital Registration - Deaths 2018 ICD10. as it appears in WHO Mortality Database Version March 2021	Thailand	2018
World Health Organization (WHO). Thailand Vital Registration - Deaths 2019 ICD10. as it appears in WHO Mortality Database Version March 2021	Thailand	2019
Department of Peace and Conflict Research, Uppsala University. UCDP Georeferenced Event Dataset, Version 17.1, 2016. Uppsala, Sweden: Department of Peace and Conflict Research, Uppsala University, 2017.	Thailand	2016
United Nations Office on Drugs and Crime (UNODC). United Nations Office on Drugs and Crime Global Study on Homicide 2011. Vienna, Austria: United Nations Office on Drugs and Crime (UNODC), 2011.	Thailand	2011
INDEPTH. Africa, Asia, Oceania - INDEPTH Network Cause-Specific Mortality - Release 2014. Accra, Ghana: INDEPTH, 2014.	Viet Nam	2014
Huong DL, Minh HV, Byass P. Applying verbal autopsy to determine cause of death in rural Vietnam. Scand J Public Health Suppl. 2003; 62: 19-25.	Viet Nam	2003
Hoa DP, Höjer B, Persson LA. Are there social inequities in child morbidity and mortality in rural Vietnam. J Trop Pediatr. 1997; 43(4): 226-31.	Viet Nam	1997
Climate Change and African Political Stability Project (CCAPS). Armed Conflict Location and Event Dataset, Realtime - Robert S. Strauss Center as referenced in Raleigh, Clionadh, Andrew Linke, Havard Hegre and Joakim Karlsen. 2010. Introducing ACLED-Armed Conflict Location and Event Data. Journal of Peace Research 47(5), 651-60.	Viet Nam	2010
Peace Research Institute Oslo (PRIO). Battle Deaths Dataset Version 3.1, 2009. Oslo, Norway: Peace Research Institute Oslo (PRIO), 2009.	Viet Nam	2009
Nga NT, Hoa DTP, Målqvist M, Persson L-Å, Ewald U. Causes of neonatal death: results from NeoKIP community-based trial in Quang Ninh province, Vietnam. Acta Paediatr. 2012; 101(4): 368-73.	Viet Nam	2012
Hong TT, Phuong Hoa N, Walker SM, Hill PS, Rao C. Completeness and reliability of mortality data in Viet Nam: Implications for the national	Viet Nam	2018

routine health management information system. PLoS One. 2018; 13(1): e0190755.		
Amnesty International. Death Sentences and Executions 1991-2011.	Viet Nam	1991-2011
Amnesty International. Death Sentences and Executions 2007-2009,2013, 2018-2021. London, United Kingdom: Amnesty International, 2008.	Viet Nam	2007-2009, 2013, 2018-2021
Centre for Research on the Epidemiology of Disasters (CRED). EM-DAT: The OFDA/CRED International Disaster Database. Brussels, Belgium: Catholic University of Leuven.	Viet Nam	NA
National Consortium for the Study of Terrorism and Responses to Terrorism (START). Global Terrorism Database. College Park , MD, United States of America: University of Maryland.	Viet Nam	NA
International Institute for Strategic Studies. International Institute for Strategic Studies Armed Conflict Database. London, United Kingdom: International Institute for Strategic Studies.	Viet Nam	NA
International Organization for Migration (IOM). Missing Migrants Project 2014-2020. Geneva, Switzerland: International Organization for Migration (IOM).	Viet Nam	2014-2020
Hoa NP, Rao C, Hoy DG, Hinh ND, Chuc NT, Ngo DA. Mortality measures from sample-based surveillance: evidence of the epidemiological transition in Viet Nam. Bull World Health Organ. 2012; 90(10): 764-72.	Viet Nam	2012
Hoa NP, Rao C, Hoy DG, Hinh ND, Chuc NT, Ngo DA. Mortality measures from sample-based surveillance: evidence of the epidemiological transition in Viet Nam. Bull World Health Organ. 2012; 90(10): 764-72. [Unpublished data].	Viet Nam	2012
Ngo AD, Rao C, Hoa NP, Adair T, Chuc NTK. Mortality patterns in Vietnam, 2006: Findings from a national verbal autopsy survey. BMC Res Notes. 2010; 3: 78.	Viet Nam	2006
Quyen BTT, Nhung NT, Cuong PV. The causes of deaths in Chililab between 2008-2010 based on verbal autopsy method. Vietnam J Public Health. 2012; 1(1): 24-31.	Viet Nam	2008-2010
United Nations Office on Drugs and Crime (UNODC). United Nations Office on Drugs and Crime Global Study on Homicide 2011. Vienna, Austria: United Nations Office on Drugs and Crime (UNODC), 2011.	Viet Nam	2011
Hanoi School of Public Health, Ministry of Health (Vietnam), School of Population Health, University of Queensland (Australia). Vietnam Burden of Disease and Injury Study 2008.	Viet Nam	2008
General Statistics Office (Vietnam). Vietnam Population Change and Family Planning Survey 2006.	Viet Nam	2006

Table S5 Data sources used in estimation of non-fatal outcomes of injuries in the ten ASEAN countries

Citation	Country	Year
Macro International, Inc, Ministry of Health (Cambodia), National Institute of Statistics (Cambodia). Cambodia Demographic and Health Survey 2000. Fairfax, United States of America: ICF International.	Cambodia	NA
Macro International, Inc, National Institute of Public Health (Cambodia), National Institute of Statistics (Cambodia). Cambodia Demographic and Health Survey 2005-2006. Fairfax, United States of America: ICF International.	Cambodia	2005-2006
ICF International, Ministry of Health (Cambodia), National Institute of Statistics (Cambodia). Cambodia Demographic and Health Survey 2014. Fairfax, United States of America: ICF International, 2017.	Cambodia	2014
Ministry of Health (Indonesia). Indonesia Integrated Hospital Data 2013. Jakarta, Indonesia: Ministry of Health (Indonesia), 2014.	Indonesia	2013
World Health Organization (WHO). Laos World Health Survey 2003.	Laos	2003
World Health Organization (WHO). Malaysia World Health Survey 2003. Geneva, Switzerland: World Health Organization (WHO), 2005.	Malaysia	2003
ICF International, Ministry of Health and Sports (Myanmar). Myanmar Demographic and Health Survey 2015-2016. Fairfax, United States of America: ICF International, 2017.	Myanmar	2015-2016
World Health Organization (WHO). Myanmar World Health Survey 2003. Geneva, Switzerland: World Health Organization (WHO), 2005.	Myanmar	2003
Philippine Health Insurance Corporation. Philippine Health Insurance Corporation Claims 2013-2016.	Philippines	2013-2016
Macro International, Inc, National Statistics Office (Philippines). Philippines Demographic and Health Survey 2008. Fairfax, United States of America: ICF International, 2010.	Philippines	2008
ICF International, Philippines Statistics Authority. Philippines Demographic and Health Survey 2013. Fairfax, United States of America: ICF International, 2014.	Philippines	2013
World Health Organization (WHO). Philippines World Health Survey 2003. Geneva, Switzerland: World Health Organization (WHO), 2005.	Philippines	2003
Institute of Health Research, Chulalongkorn University (Thailand), Ministry of Public Health (Thailand), The Alliance for Safe Children (TASC), United Nations Children's Fund (UNICEF). Thailand National Injury Survey 2003-2004.	Thailand	2003-2004
Hang HM, Ekman R, Bach TT, Byass P, Svanström L. Community-based assessment of unintentional injuries: a pilot study in rural Vietnam. Scand J Public Health Suppl. 2003; 38-44.	Viet Nam	2003
Nguyen TLH, Nguyen THT, Morita S, Sakamoto J. Injury and pre-hospital trauma care in Hanoi, Vietnam. Injury. 2008; 39(9): 1026-33.	Viet Nam	2008
Nguyen TV, Dalman C, Le TC, Nguyen TV, Tran NV, Allebeck P. Suicide attempt in a rural area of Vietnam: Incidence, methods used and access to mental health care. Int J Ment Health Syst. 2010; 4(1): 3.	Viet Nam	2010
Ministry of Health (Vietnam). Vietnam Hospital Data 2013.	Viet Nam	2013

Hanoi School of Public Health, Ministry of Health (Vietnam), Ministry of Labour, Invalids and Social Affairs (MOLISA) (Vietnam), World Health Organization (WHO). Vietnam Injury Survey 2010.[Unpublished].	Viet Nam	2010
Hanoi School of Public Health, The Alliance for Safe Children (TASC), United Nations Children's Fund (UNICEF). Vietnam Multi-Center Injury Survey 2001.	Viet Nam	2001
World Health Organization (WHO). Vietnam World Health Survey 2002-2003. Geneva, Switzerland: World Health Organization (WHO), 2005.	Viet Nam	2002-2003

Statistical analysis

Causes of death modelling

Various standardised procedures were applied to ensure causes of death data from various sources aligned on the specific definitions and were properly mapped to the GBD cause list. For data with ill-defined causes, algorithms were applied to redistribute deaths to the appropriate categories.[1] Once data were processed, the GBD Cause of Death Ensemble model (CODEm) method was applied to generate cause-specific mortality estimates by age, sex, location, year, and cause of injury. CODEm is a model-averaging meta-analytical approach that combines estimates from multiple submodels to derive results. Out-of-sample cross-validation was performed for all estimations to ensure optimal accuracy. Estimates generated from CODEm for each cause of injury were subsequently rescaled to ensure coherent and consistent estimates across the cause hierarchy. Further details can be found in a previous publication.[1]

Disease model—Bayesian meta-regression (DisMod-MR) 2.1

DisMod-MR 2.1 is a tool for estimating non-fatal outcomes of injury. A core feature of DisMod-MR 2.1 is the application of mixed-effects models for estimating levels and trends while accounting for heterogeneity across data sources and populations. In addition, to achieve coherence among metrics, transitions between different disease states (eg, incidence, remission, and mortality) in the compartmental model were determined by solving a set of ordinary differential equations. Depending on the cause, specific priors may be introduced in the modelling process. These may include hierarchical priors to represent geographical hierarchy, informative priors to reflect expert knowledge on the level and variability of a disease or injury, and age-specific priors to capture inherent age patterns in specific conditions. A distinct model was developed for each cause of injury, and incidence was estimated by age, sex, location, and year. Data sources integrated in the analysis included inpatient data, outpatient data, surveillance data, survey data, and literature studies that are population-representative. Further details can be found in previous publications.[2,3]

Estimation of incidence, prevalence and YLD

The estimation of the incidence and prevalence of each injury cause involves first determining the distribution of the nature of injuries associated with each cause. Using clinical data sources containing both cause and nature of injuries, the probability of the nature of injuries was determined for each new injury case and applied to the total incidence to establish the cause-nature distribution. This process was conducted separately for inpatient and outpatient incidences. To derive prevalence from incidence, additional analytical steps were taken to account for the duration of disability (long- versus short-term). Incidences with long-term disability were converted to prevalence using specific ordinary differential equation solver functions in DisMod-MR 2.1. Short-term incidence was converted to prevalence by multiplying incidence with the duration of injury, which was derived from longitudinal studies or expert opinion. After reconciling short-term and long-term prevalence estimates and aligning them

with the nature of injuries, YLDs for each cause of injury were calculated as the prevalence of each cause-nature multiplied by the corresponding disability weight,[4,5] then summed across the nature of injuries for each cause by age, sex, location, and year. Further details can be found in a previous publication [2] and GBD 2021 online methods appendices (https://www.healthdata.org/gbd/methods-appendices-2021).

Age standardisation

To calculate age-standardised rates, age-specific rates estimated from the analytical models were weighted using the GBD standard population.[6] Subsequently, the weighted age-specific rates were summed across all age groups to produce the age-standardised rate, allowing for comparisons over time and across geography without demographic structure confounding. Details of these calculations can be found in previous publications.[2,3]

GATHER Checklist

Item #	Checklist item	Reporting location
Objectives and funding		
1	Define the indicator(s), populations (including age, sex, and geographic entities), and time period(s) for which estimates were made.	Main text – Methods, Overview
2	List the funding sources for the work.	Main text – Acknowledgement section
Data Inputs		
For all data inputs from multiple sources that are synthesized as part of the study:		
3	Describe how the data were identified and how the data were accessed.	Main text – Methods, Overview<br>Supplementary Appendix – Supplementary Methods, Tables S4 and S5
4	Specify the inclusion and exclusion criteria. Identify all ad-hoc exclusions.	Main text – Methods with reference to previous publications
5	Provide information on all included data sources and their main characteristics. For each data source used, report reference information or contact name/institution, population represented, data collection method, year(s) of data collection, sex and age range, diagnostic criteria or measurement method, and sample size, as relevant.	Supplementary Appendix – Supplementary Methods, Figure S1 and Table S1
6	Identify and describe any categories of input data that have potentially important biases (e.g., based on characteristics listed in item 5).	Main text – Limitations subsection
For data inputs that contribute to the analysis but were not synthesized as part of the study:		
7	Describe and give sources for any other data inputs.	N/A
For all data inputs:		
8	Provide all data inputs in a file format from which data can be efficiently extracted (e.g., a spreadsheet rather than a PDF), including all relevant meta-data listed in item 5. For any data inputs that cannot be shared because of ethical or legal reasons, such as third-party ownership, provide a contact name or the name of the institution that retains the right to the data.	Data inputs in a spreadsheet format are available on the GHDx at https://ghdx.healthdata.org/gbd-2021/sources
Data analysis		
9	Provide a conceptual overview of the data analysis method. A diagram may be helpful.	Main text – Methods<br>Supplementary Appendix – Supplementary Methods
10	Provide a detailed description of all steps of the analysis, including mathematical formulae. This description should cover, as relevant, data cleaning, data pre-processing, data adjustments and weighting of data sources, and mathematical or statistical model(s).	Main text – Methods with additional references from previous publications
11	Describe how candidate models were evaluated and how the final model(s) were selected.	Supplemental Appendix – Supplemental Methods, Model flow charts
12	Provide the results of an evaluation of model performance, if done, as well as the results of any relevant sensitivity analysis.	N/A
13	Describe methods for calculating uncertainty of the estimates. State which sources of uncertainty were, and were not, accounted for in the uncertainty analysis.	Main text – Methods

14	State how analytic or statistical source code used to generate estimates can be accessed.	The source code can be found on the GHDx at https://ghdx.healthdata.org/gbd-2021/code
Results and Discussion		
15	Provide published estimates in a file format from which data can be efficiently extracted.	The results can be efficiently extracted at https://vizhub.healthdata.org/gbd-results/
16	Report a quantitative measure of the uncertainty of the estimates (e.g. uncertainty intervals).	95% Uncertainty Intervals (UIs) given for all findings, including in the text, figures, and tables in the main text and Supplemental Appendix; online viz tools (see information above)
17	Interpret results in light of existing evidence. If updating a previous set of estimates, describe the reasons for changes in estimates.	Main text – Discussion section
18	Discuss limitations of the estimates. Include a discussion of any modelling assumptions or data limitations that affect interpretation of the estimates.	Main text – Discussion, Limitations subsection

Supplemental results

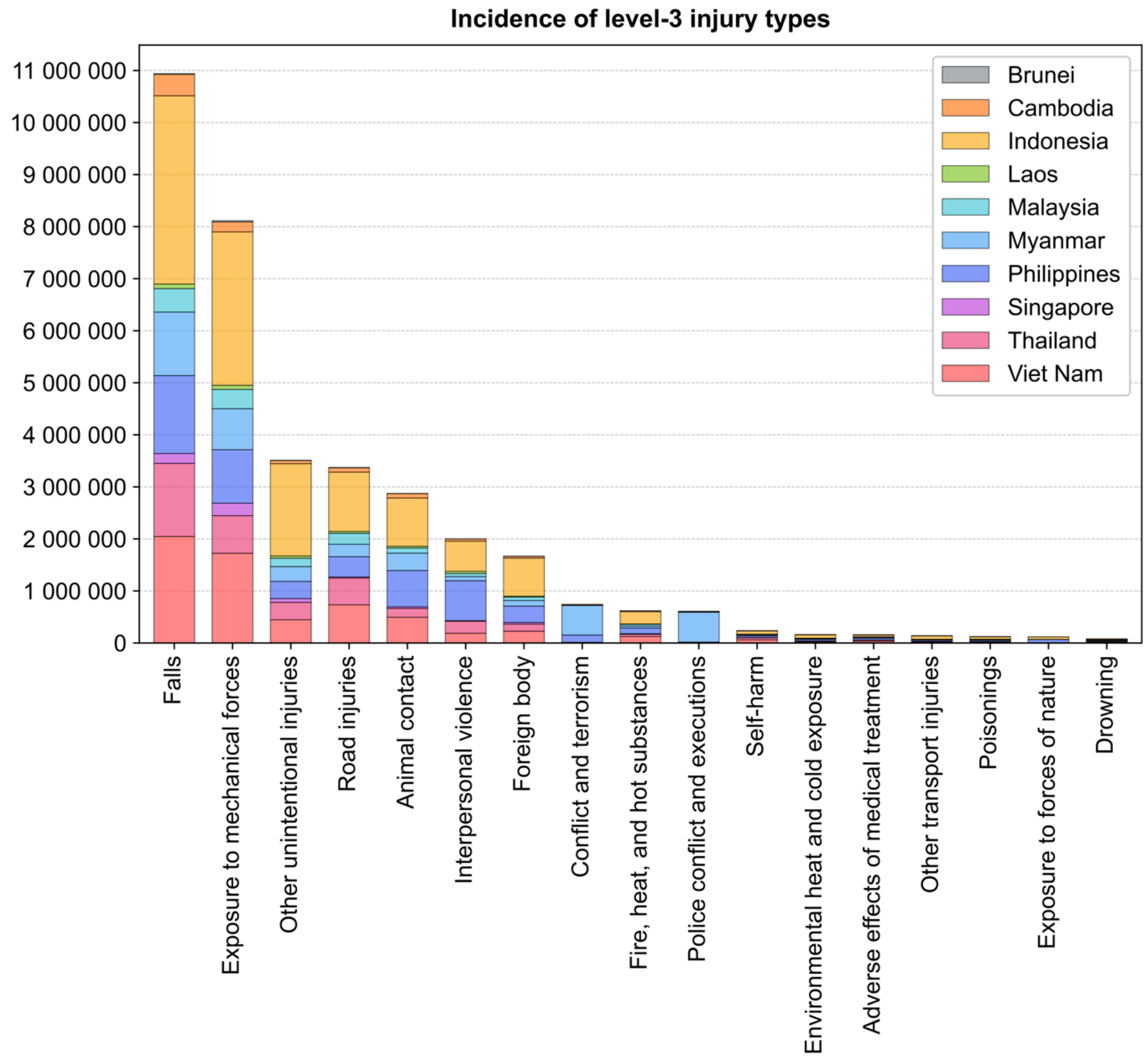

Figure S1 | Incident cases for Level 3 injury causes by country, 2021.

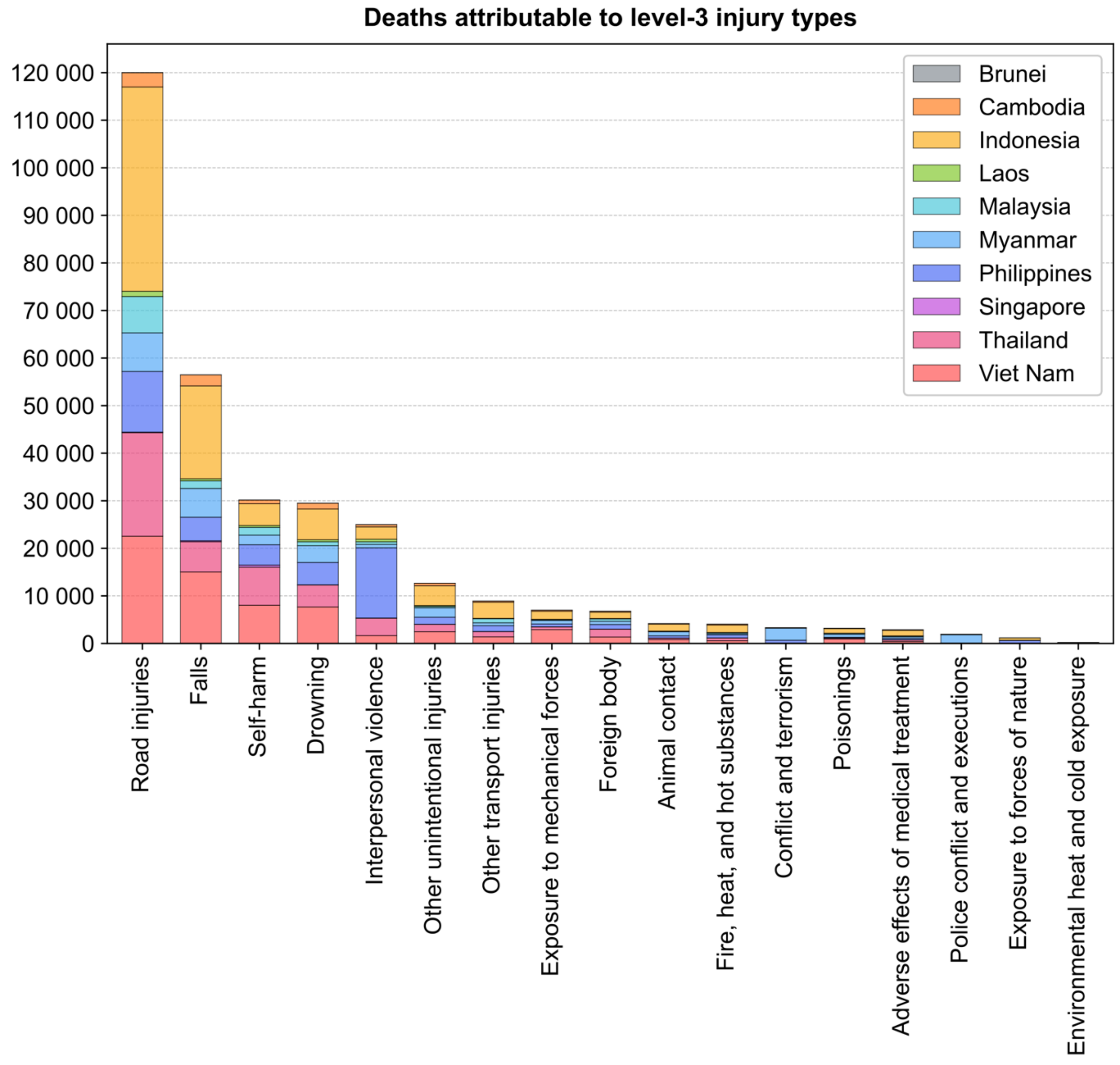

Figure S2 | Number of deaths attributable to Level 3 injury causes by country, 2021.

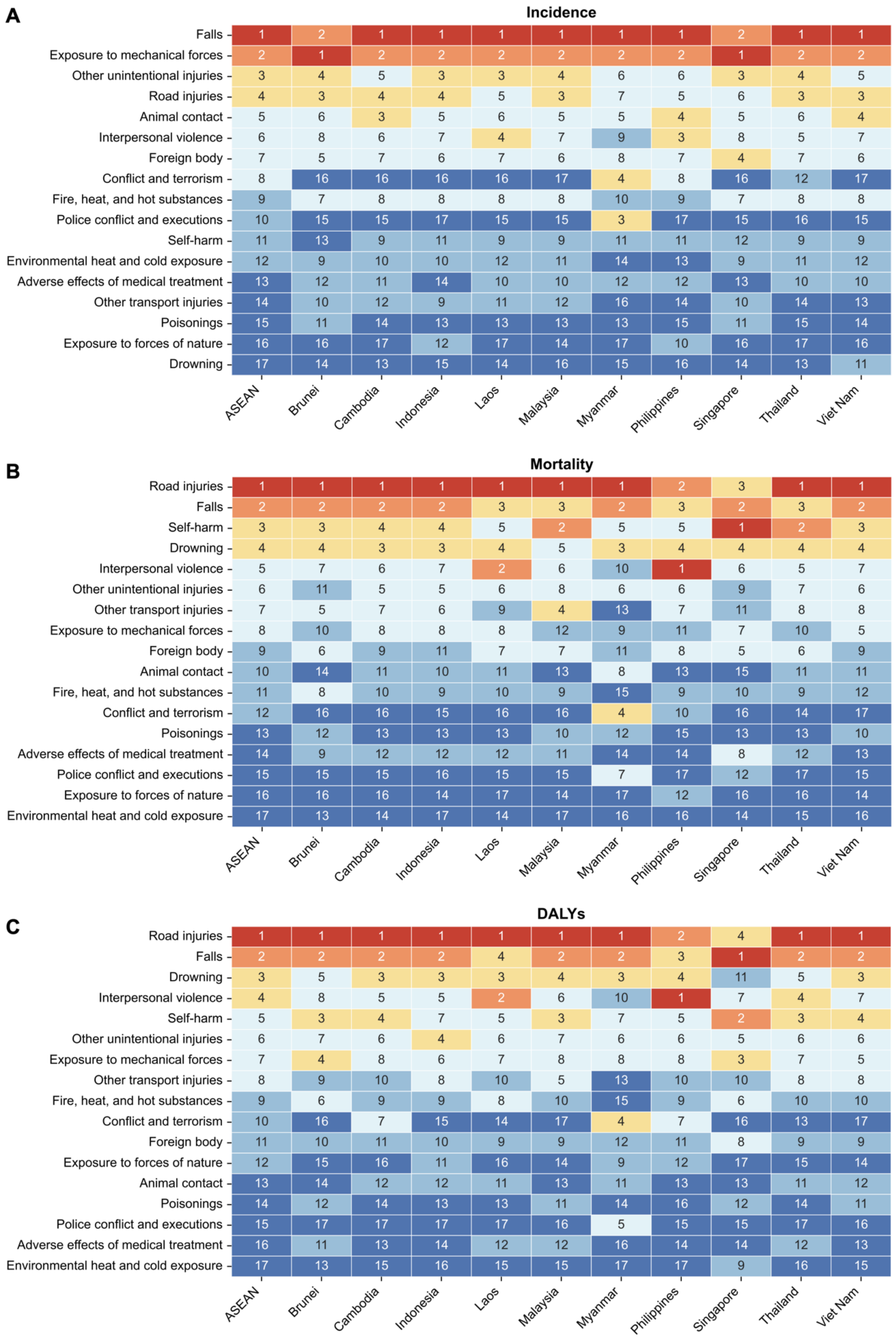

Figure S3 | Rankings of Level 3 injury causes based on the rates of (A) incidence, (B) deaths, (C) DALYs for ASEAN and its member countries, 2021.

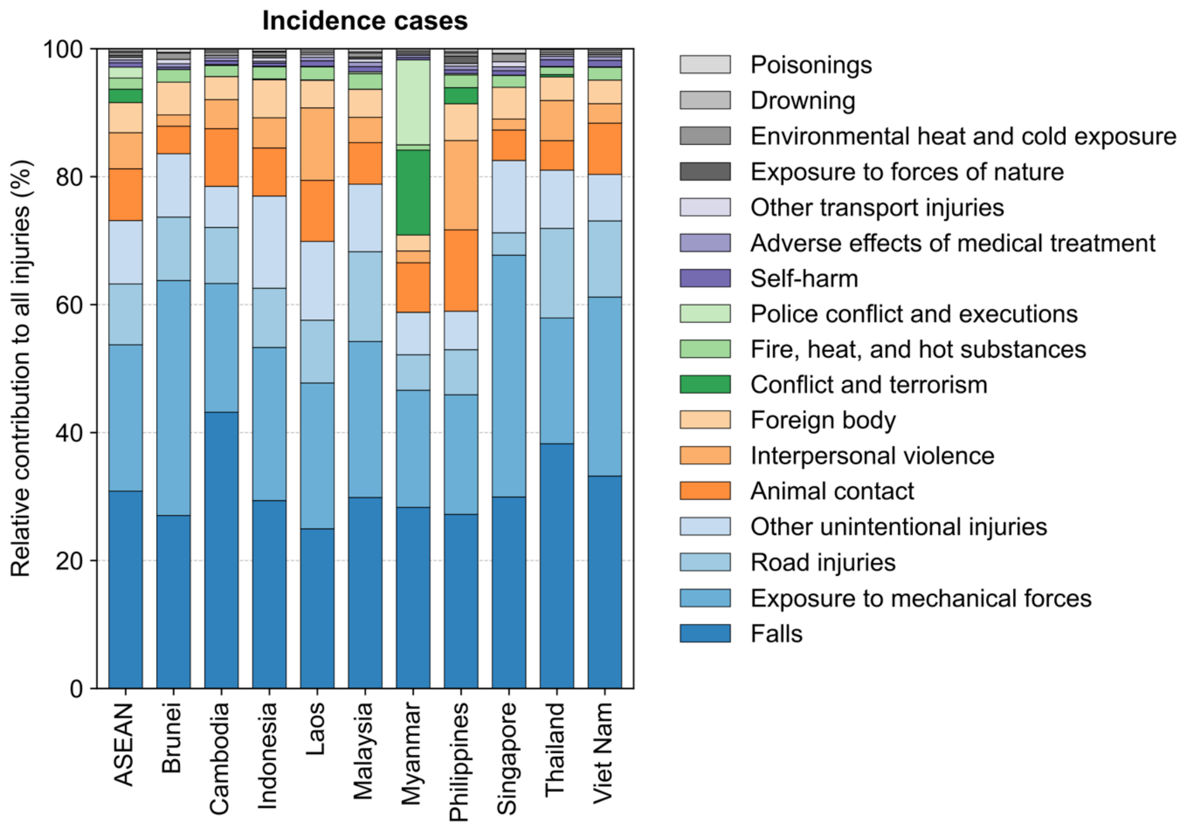

Figure S4 | Relative contribution (%) of incidence cases to total injury incidence cases for Level 3 injury causes in ASEAN and by country, 2021.

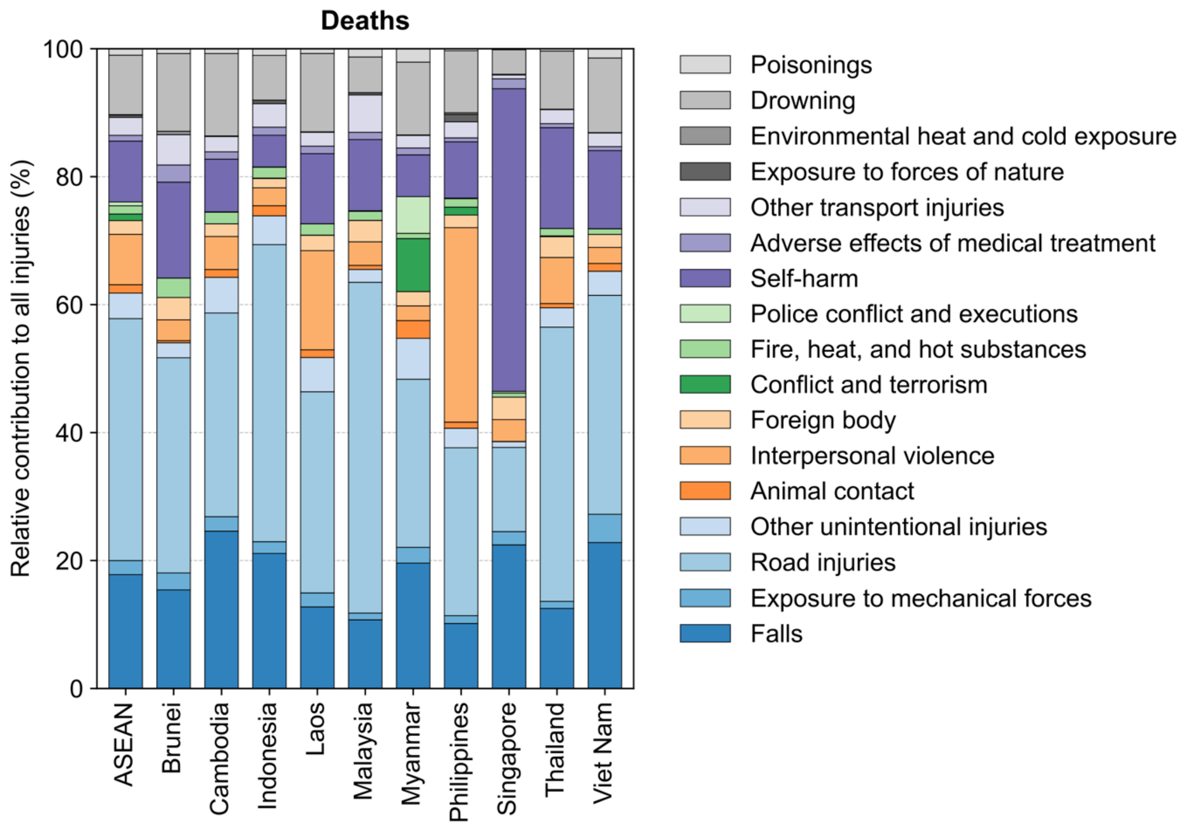

Figure S5 | Relative contribution (%) of Level 3 cause-specific deaths to total injury deaths in ASEAN and by country, 2021.

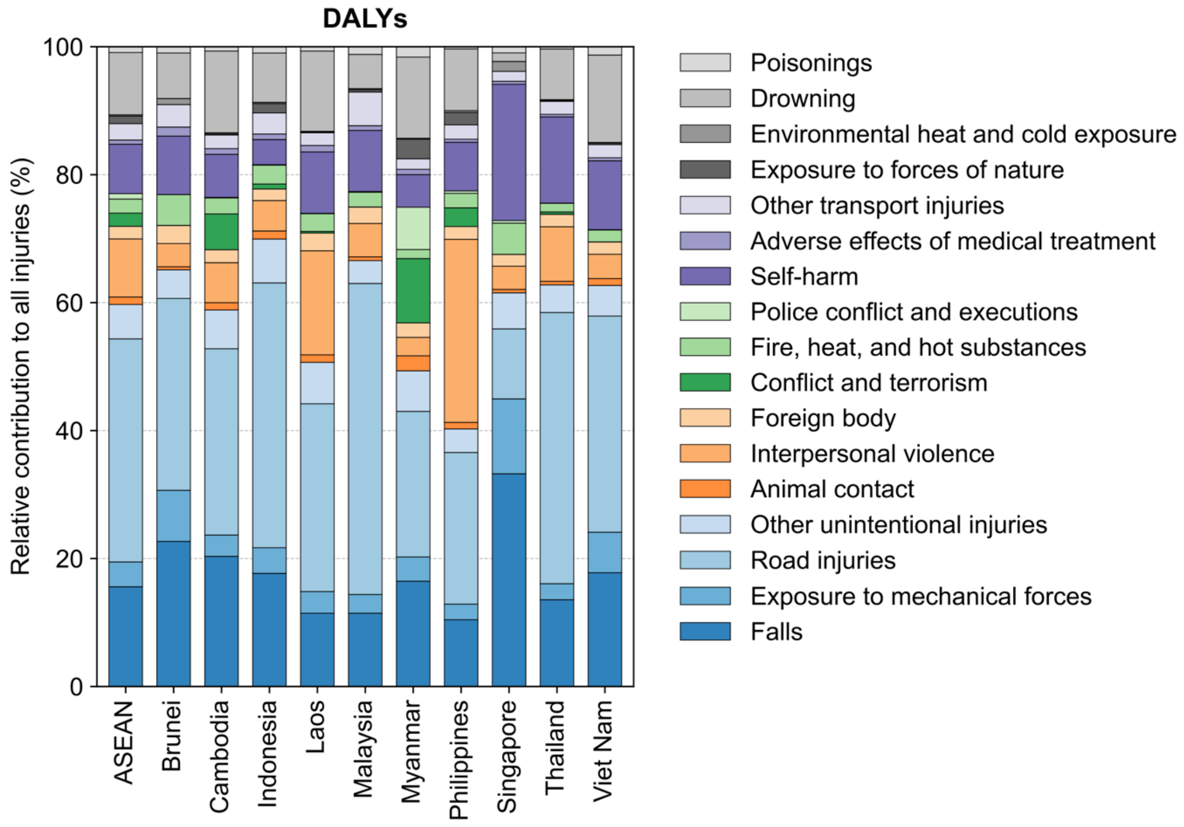

Figure S6 | Relative contribution (%) of Level 3 cause-specific DALYs to total injury-related DALYs in ASEAN and by country, 2021.

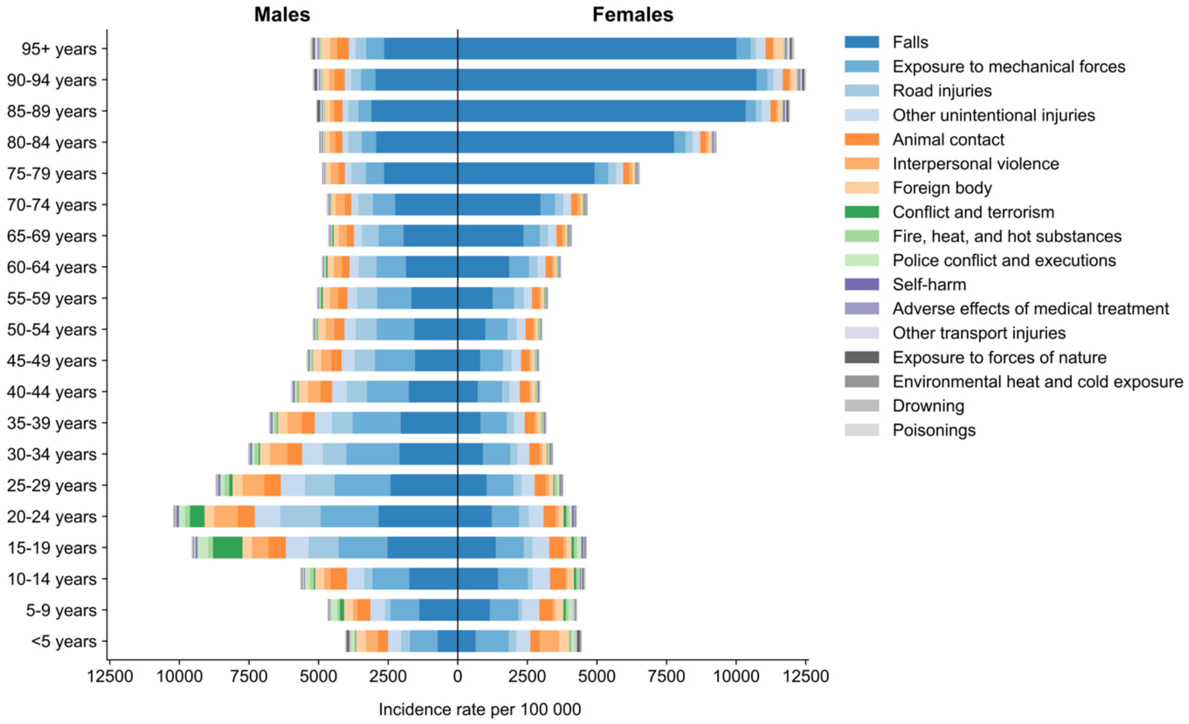

Figure S7 | Incidence rate per 100 000 attributable to Level 3 injuries, by sex and age group in ASEAN, 2021.

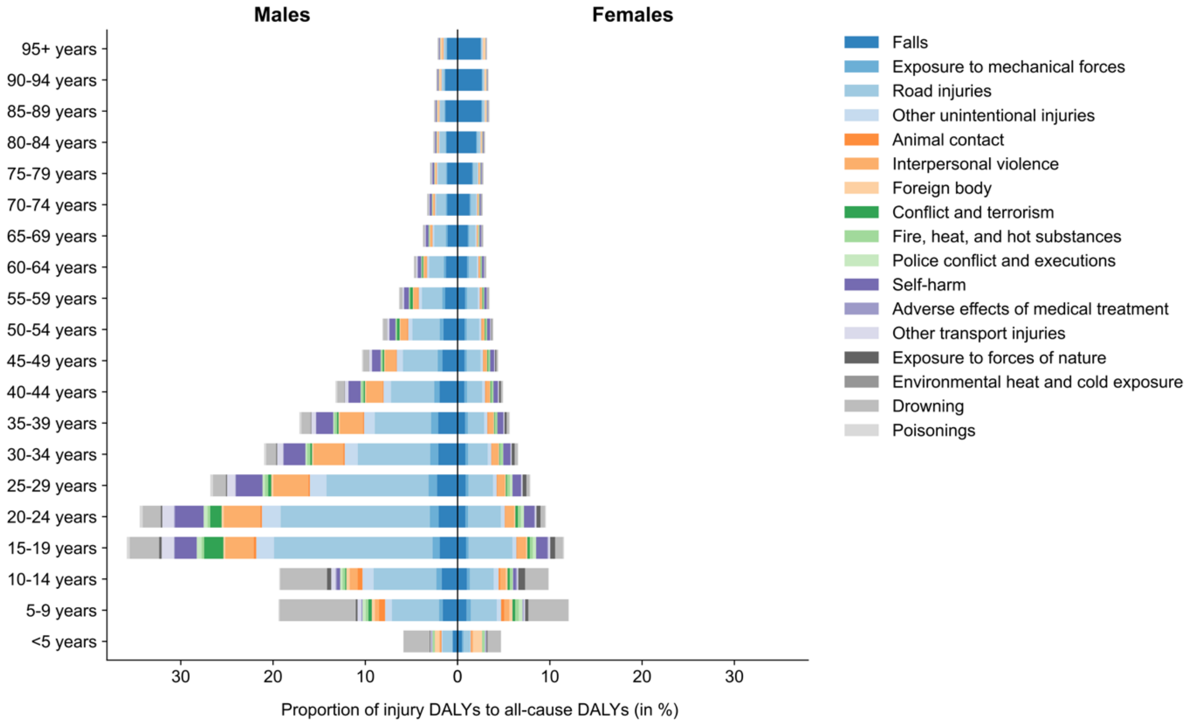

Figure S8 | Proportion of Level 3 cause-specific DALYs to all-cause DALYs (in %) by sex and age group in ASEAN, 2021.

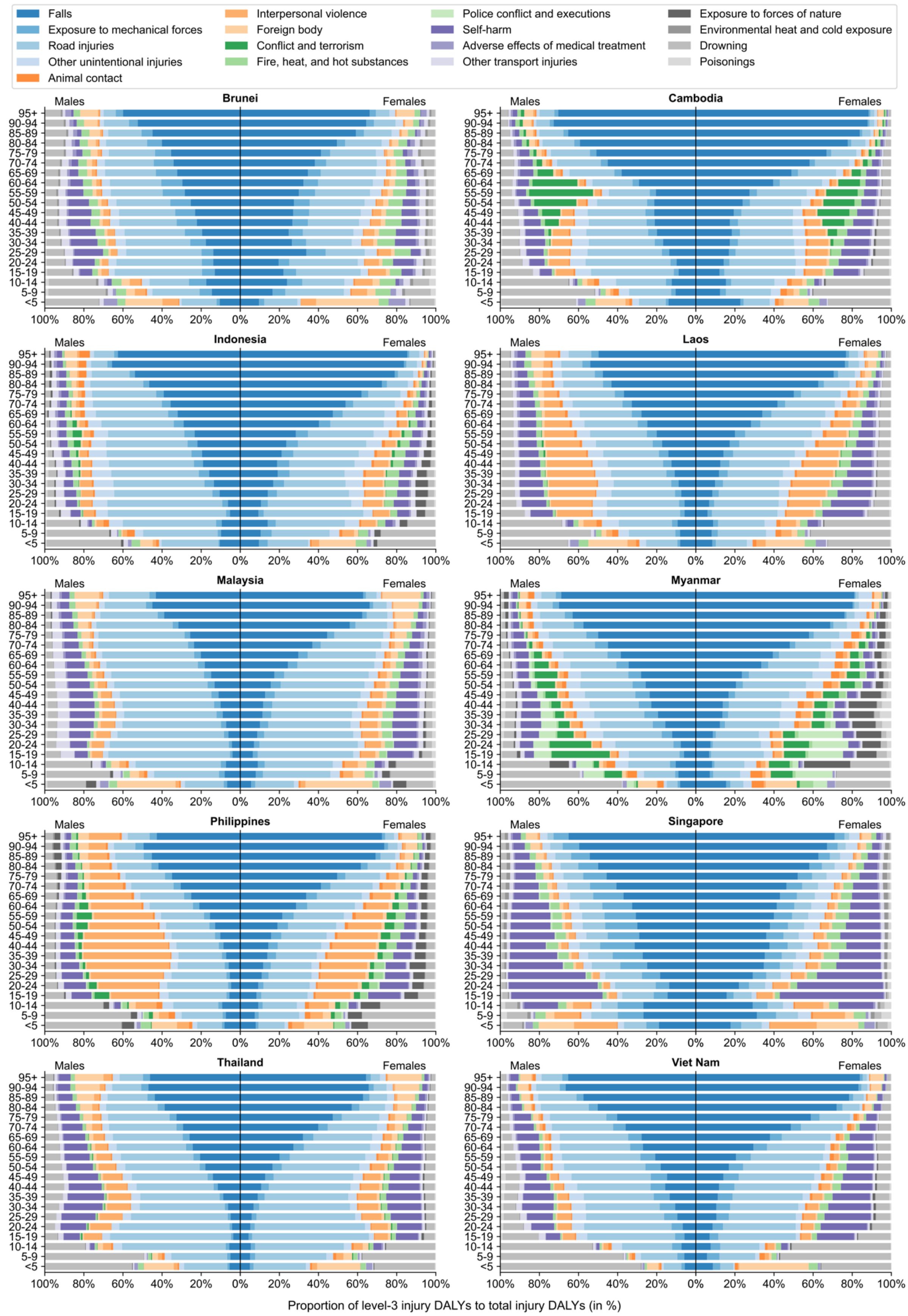

Figure S9 | Relative contribution (%) of Level 3 cause-specific DALYs to the total injury DALYs by country, sex and age group in 2021.

Contribution of road injury DALYs to all-cause DALYs

A (males)

	<5	5-9	10-14	15-19	20-24	25-29	30-34	35-39	40-44	45-49	50-54	55-59	60-64	65-69	70-74	75-79	80-84	85-89	90-94	95+
ASEAN	1.1 (0.9–1.2)	5.1 (4.1–6.2)	6.8 (5.5–8.4)	17.2 (14.6–19.8)	16.2 (14.1–18.6)	11.1 (9.5–12.8)	7.9 (6.8–9.1)	6.1 (5.3–7.0)	4.7 (4.1–5.3)	3.8 (3.3–4.3)	3.0 (2.6–3.4)	2.2 (1.9–2.5)	1.6 (1.4–1.8)	1.3 (1.1–1.4)	1.1 (0.9–1.2)	0.9 (0.8–1.0)	0.7 (0.6–0.7)	0.5 (0.4–0.6)	0.3 (0.2–0.3)	0.2 (0.2–0.3)
Brunei	1.3 (1.0–1.6)	3.0 (2.1–4.2)	2.2 (1.5–3.0)	8.1 (5.7–10.9)	11.8 (8.8–14.8)	10.9 (8.3–13.5)	7.3 (5.7–9.2)	5.8 (4.7–7.2)	4.5 (3.7–5.4)	3.7 (3.0–4.6)	2.9 (2.4–3.5)	2.3 (1.9–2.7)	1.8 (1.5–2.2)	1.6 (1.3–1.9)	1.3 (1.1–1.6)	1.0 (0.9–1.2)	0.8 (0.7–1.0)	0.6 (0.5–0.7)	0.4 (0.3–0.5)	0.1 (0.1–0.2)
Cambodia	0.5 (0.4–0.7)	4.6 (3.1–6.9)	5.2 (3.2–7.7)	15.4 (10.4–21.6)	16.0 (11.2–21.0)	11.3 (7.4–15.4)	7.8 (5.5–10.9)	6.1 (4.4–8.2)	4.8 (3.4–6.5)	3.8 (2.6–5.1)	2.9 (2.0–3.9)	2.1 (1.5–3.0)	1.4 (1.0–2.0)	1.1 (0.8–1.6)	0.9 (0.6–1.2)	0.7 (0.5–1.0)	0.5 (0.4–0.7)	0.4 (0.3–0.5)	0.2 (0.2–0.3)	0.3 (0.2–0.3)
Indonesia	1.7 (1.4–1.9)	6.5 (5.0–8.1)	8.3 (6.3–10.4)	19.0 (15.5–22.7)	16.0 (13.2–19.2)	9.7 (7.7–12.1)	6.6 (5.2–8.3)	5.0 (3.9–6.2)	3.7 (2.9–4.5)	2.9 (2.3–3.5)	2.3 (1.9–2.7)	1.6 (1.4–1.9)	1.1 (0.9–1.3)	0.9 (0.8–1.1)	0.8 (0.7–1.0)	0.7 (0.6–0.9)	0.5 (0.4–0.6)	0.4 (0.3–0.4)	0.1 (0.1–0.2)	0.2 (0.1–0.2)
Laos	0.5 (0.3–0.6)	5.1 (3.3–7.2)	5.6 (3.7–8.2)	13.1 (9.2–18.1)	13.9 (9.4–19.2)	10.0 (7.1–13.7)	7.0 (4.8–9.9)	5.3 (3.6–7.3)	4.1 (2.9–5.6)	3.2 (2.2–4.6)	2.4 (1.7–3.2)	1.8 (1.3–2.4)	1.2 (0.9–1.6)	0.9 (0.7–1.3)	0.7 (0.6–1.0)	0.6 (0.4–0.7)	0.4 (0.3–0.6)	0.3 (0.2–0.4)	0.2 (0.1–0.2)	0.2 (0.2–0.3)
Malaysia	0.8 (0.6–1.0)	3.6 (2.3–5.1)	6.5 (4.6–8.6)	23.7 (19.0–28.5)	23.6 (19.4–27.9)	15.7 (12.6–19.1)	11.3 (8.7–14.4)	8.3 (6.5–10.3)	6.5 (4.9–8.5)	5.2 (3.8–6.9)	4.2 (3.2–5.4)	3.3 (2.5–4.4)	2.6 (2.0–3.5)	2.3 (1.7–3.0)	2.0 (1.5–2.6)	1.7 (1.3–2.2)	1.2 (0.9–1.6)	0.8 (0.6–1.0)	0.5 (0.4–0.6)	0.4 (0.3–0.6)
Myanmar	0.4 (0.3–0.6)	3.9 (2.5–6.0)	5.2 (3.5–7.3)	11.5 (8.3–15.4)	12.0 (8.8–16.0)	8.6 (6.2–11.6)	5.5 (3.9–7.7)	4.0 (2.8–5.5)	3.0 (2.2–4.1)	2.4 (1.7–3.2)	2.1 (1.5–2.8)	1.5 (1.1–2.0)	1.1 (0.8–1.5)	0.8 (0.6–1.1)	0.7 (0.5–0.9)	0.5 (0.4–0.7)	0.4 (0.3–0.5)	0.3 (0.2–0.4)	0.2 (0.1–0.3)	0.2 (0.1–0.3)
Philippines	0.5 (0.4–0.5)	3.5 (2.8–4.2)	3.6 (2.8–4.4)	8.5 (6.8–10.2)	10.2 (8.6–11.8)	7.8 (6.8–8.8)	5.5 (4.9–6.1)	4.1 (3.7–4.6)	3.0 (2.7–3.3)	2.3 (2.1–2.5)	1.7 (1.5–1.8)	1.2 (1.1–1.3)	0.8 (0.7–0.9)	0.6 (0.5–0.6)	0.5 (0.4–0.5)	0.4 (0.3–0.4)	0.3 (0.3–0.3)	0.2 (0.2–0.2)	0.1 (0.1–0.1)	0.1 (0.1–0.2)
Singapore	0.6 (0.5–0.7)	1.0 (0.8–1.3)	0.7 (0.6–0.9)	3.1 (2.3–4.0)	5.2 (4.2–6.4)	4.5 (3.7–5.5)	2.6 (2.2–3.0)	1.9 (1.7–2.3)	1.4 (1.3–1.6)	1.3 (1.2–1.5)	1.2 (1.1–1.4)	1.0 (0.9–1.1)	0.9 (0.8–1.0)	0.7 (0.6–0.8)	0.6 (0.6–0.7)	0.5 (0.4–0.5)	0.4 (0.3–0.4)	0.3 (0.2–0.3)	0.2 (0.2–0.2)	0.1 (0.1–0.1)
Thailand	2.0 (1.5–2.5)	6.7 (4.8–8.9)	13.8 (10.7–17.6)	31.2 (25.4–36.5)	28.2 (23.7–33.3)	21.6 (17.7–25.6)	15.5 (12.4–19.3)	11.3 (9.1–14.0)	8.4 (6.5–10.2)	6.3 (5.0–8.0)	4.8 (3.9–5.9)	3.7 (3.0–4.7)	2.8 (2.2–3.5)	2.3 (1.8–2.9)	1.9 (1.5–2.3)	1.4 (1.1–1.7)	0.9 (0.7–1.1)	0.6 (0.5–0.8)	0.4 (0.3–0.5)	0.3 (0.2–0.4)
Viet Nam	1.0 (0.7–1.5)	5.1 (3.4–7.5)	6.7 (4.5–9.5)	19.0 (13.5–25.0)	18.4 (13.8–24.1)	12.2 (8.9–16.2)	9.7 (6.7–13.0)	8.4 (6.2–11.1)	7.6 (5.6–10.1)	6.7 (5.0–8.9)	5.3 (3.9–7.0)	4.0 (3.0–5.3)	2.9 (2.1–3.7)	2.2 (1.6–2.9)	1.6 (1.3–2.1)	1.3 (1.0–1.7)	1.0 (0.7–1.3)	0.8 (0.6–1.1)	0.5 (0.4–0.6)	0.4 (0.3–0.6)

B (females)

	<5	5-9	10-14	15-19	20-24	25-29	30-34	35-39	40-44	45-49	50-54	55-59	60-64	65-69	70-74	75-79	80-84	85-89	90-94	95+
ASEAN	0.7 (0.6–0.8)	2.8 (2.3–3.5)	2.6 (1.9–3.3)	4.7 (3.6–6.2)	3.6 (2.8–4.5)	2.7 (2.1–3.3)	2.1 (1.7–2.6)	1.7 (1.4–2.1)	1.7 (1.4–2.0)	1.5 (1.2–1.8)	1.3 (1.1–1.6)	1.2 (1.0–1.3)	0.9 (0.8–1.0)	0.7 (0.6–0.8)	0.6 (0.5–0.7)	0.5 (0.4–0.5)	0.3 (0.3–0.3)	0.2 (0.2–0.3)	0.1 (0.1–0.2)	0.1 (0.1–0.1)
Brunei	0.9 (0.7–1.2)	3.0 (2.0–4.2)	1.5 (1.1–2.2)	2.8 (1.9–3.9)	3.6 (2.6–5.1)	1.3 (1.1–1.6)	1.9 (1.5–2.5)	2.0 (1.6–2.7)	1.8 (1.5–2.3)	1.7 (1.4–2.1)	1.6 (1.3–2.0)	1.5 (1.3–1.8)	1.4 (1.1–1.7)	1.3 (1.1–1.6)	1.1 (1.0–1.4)	0.9 (0.8–1.1)	0.7 (0.5–0.8)	0.5 (0.4–0.6)	0.3 (0.3–0.4)	0.1 (0.1–0.2)
Cambodia	0.5 (0.3–0.7)	2.9 (1.7–4.5)	2.2 (1.3–3.3)	4.6 (2.6–7.3)	3.7 (2.3–5.8)	2.6 (1.6–3.9)	2.1 (1.3–3.1)	1.7 (1.2–2.5)	1.7 (1.1–2.4)	1.5 (1.0–2.2)	1.4 (1.0–2.0)	1.2 (0.9–1.6)	0.9 (0.7–1.3)	0.7 (0.5–1.0)	0.6 (0.5–0.9)	0.5 (0.3–0.6)	0.3 (0.2–0.4)	0.2 (0.1–0.2)	0.1 (0.1–0.1)	0.1 (0.1–0.1)
Indonesia	1.0 (0.8–1.3)	2.7 (2.0–3.6)	2.8 (2.0–3.9)	5.1 (3.6–7.2)	3.3 (2.3–4.7)	2.2 (1.7–3.2)	1.6 (1.2–2.2)	1.2 (1.0–1.6)	1.3 (1.1–1.7)	1.2 (1.0–1.5)	1.1 (0.9–1.3)	0.9 (0.8–1.1)	0.7 (0.6–0.8)	0.5 (0.4–0.6)	0.5 (0.4–0.6)	0.4 (0.3–0.5)	0.3 (0.2–0.3)	0.2 (0.2–0.3)	0.1 (0.1–0.2)	0.1 (0.1–0.2)
Laos	0.4 (0.3–0.6)	3.4 (2.1–5.0)	2.7 (1.7–4.1)	4.8 (2.8–7.4)	3.9 (2.4–5.9)	2.8 (1.8–4.2)	2.1 (1.4–3.1)	1.8 (1.2–2.4)	1.6 (1.1–2.4)	1.4 (1.0–1.9)	1.3 (0.9–1.8)	1.1 (0.8–1.5)	0.9 (0.6–1.2)	0.7 (0.5–0.9)	0.6 (0.4–0.8)	0.4 (0.3–0.6)	0.3 (0.2–0.3)	0.2 (0.1–0.2)	0.1 (0.1–0.1)	0.1 (0.1–0.1)
Malaysia	0.7 (0.5–0.9)	2.5 (1.6–3.8)	2.3 (1.6–3.3)	4.6 (3.0–6.6)	4.5 (3.0–6.3)	3.4 (2.3–4.8)	2.6 (1.8–3.7)	2.3 (1.6–3.2)	2.2 (1.5–3.0)	1.9 (1.3–2.6)	1.6 (1.1–2.2)	1.4 (1.1–1.9)	1.1 (0.8–1.5)	0.9 (0.7–1.2)	0.7 (0.6–1.0)	0.6 (0.4–0.7)	0.3 (0.3–0.4)	0.2 (0.2–0.3)	0.1 (0.1–0.2)	0.1 (0.1–0.1)
Myanmar	0.3 (0.2–0.4)	2.3 (1.3–3.7)	2.2 (1.4–3.1)	4.4 (2.5–6.7)	3.5 (2.1–5.1)	2.6 (1.6–3.8)	2.0 (1.3–2.9)	1.7 (1.1–2.5)	1.5 (1.0–2.2)	1.3 (0.9–1.9)	1.3 (0.8–1.8)	1.1 (0.8–1.6)	0.8 (0.5–1.1)	0.6 (0.4–0.8)	0.4 (0.3–0.6)	0.3 (0.2–0.4)	0.2 (0.1–0.2)	0.1 (0.1–0.2)	0.1 (0.1–0.1)	0.0 (0.0–0.1)
Philippines	0.4 (0.4–0.5)	2.5 (2.0–2.9)	1.6 (1.2–1.9)	2.1 (1.6–2.6)	1.8 (1.4–2.3)	1.4 (1.1–1.7)	1.2 (0.9–1.4)	1.0 (0.8–1.1)	0.9 (0.8–1.0)	0.8 (0.7–0.9)	0.7 (0.6–0.8)	0.6 (0.6–0.7)	0.5 (0.5–0.6)	0.4 (0.4–0.5)	0.4 (0.3–0.4)	0.3 (0.3–0.3)	0.2 (0.2–0.2)	0.1 (0.1–0.1)	0.1 (0.1–0.1)	0.1 (0.1–0.1)
Singapore	0.5 (0.4–0.6)	0.7 (0.6–0.9)	0.6 (0.5–0.7)	1.2 (0.9–1.5)	0.8 (0.7–1.0)	0.6 (0.5–0.7)	0.5 (0.4–0.6)	0.6 (0.5–0.7)	0.6 (0.5–0.7)	0.6 (0.5–0.6)	0.6 (0.5–0.6)	0.5 (0.5–0.6)	0.5 (0.4–0.6)	0.5 (0.4–0.5)	0.4 (0.4–0.5)	0.3 (0.3–0.4)	0.3 (0.2–0.3)	0.2 (0.1–0.2)	0.1 (0.1–0.1)	0.1 (0.0–0.1)
Thailand	1.8 (1.3–2.3)	5.7 (4.0–7.9)	6.1 (4.1–8.6)	9.8 (6.8–13.2)	8.2 (5.6–11.4)	6.6 (4.5–9.3)	5.1 (3.6–6.8)	3.7 (2.6–5.1)	2.9 (2.2–4.1)	2.5 (1.8–3.2)	2.1 (1.6–2.7)	1.7 (1.4–2.2)	1.3 (1.1–1.7)	1.0 (0.8–1.3)	0.8 (0.6–1.0)	0.5 (0.4–0.7)	0.3 (0.3–0.4)	0.2 (0.2–0.3)	0.2 (0.1–0.2)	0.1 (0.1–0.1)
Viet Nam	0.7 (0.5–1.0)	3.3 (2.0–4.7)	2.7 (1.6–4.0)	6.0 (3.7–9.0)	4.8 (3.0–7.0)	3.6 (2.4–5.4)	3.3 (2.1–4.7)	3.2 (2.1–4.5)	3.0 (2.0–4.4)	2.8 (1.9–4.0)	2.6 (1.7–3.6)	2.2 (1.5–3.2)	1.7 (1.2–2.2)	1.3 (0.9–1.7)	1.0 (0.8–1.4)	0.8 (0.6–1.1)	0.5 (0.4–0.6)	0.4 (0.3–0.5)	0.2 (0.2–0.3)	0.1 (0.1–0.2)

Colour scale: % (0, 5, 10, 15, 20, 25, 30)

Figure S10 | Contribution (95% UI) of road injury DALYs to all-cause DALYs (in %) for **A**) males, and **B**) females, by country and age group (in years), 2021.

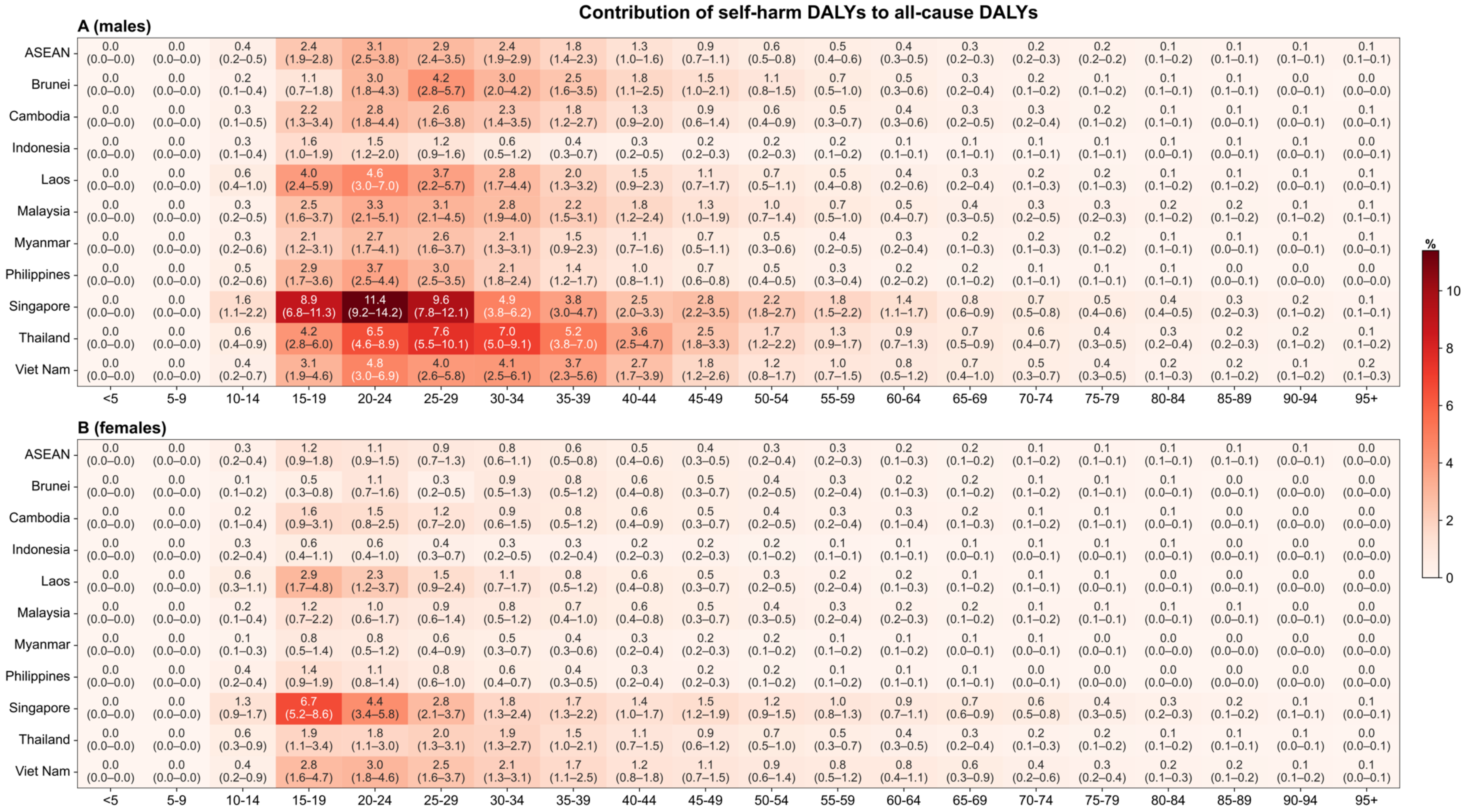

Figure S11 | Contribution (95% UI) of self-harm DALYs to all-cause DALYs (in %) for **A**) males, and **B**) females, by country and age group (in years), 2021.

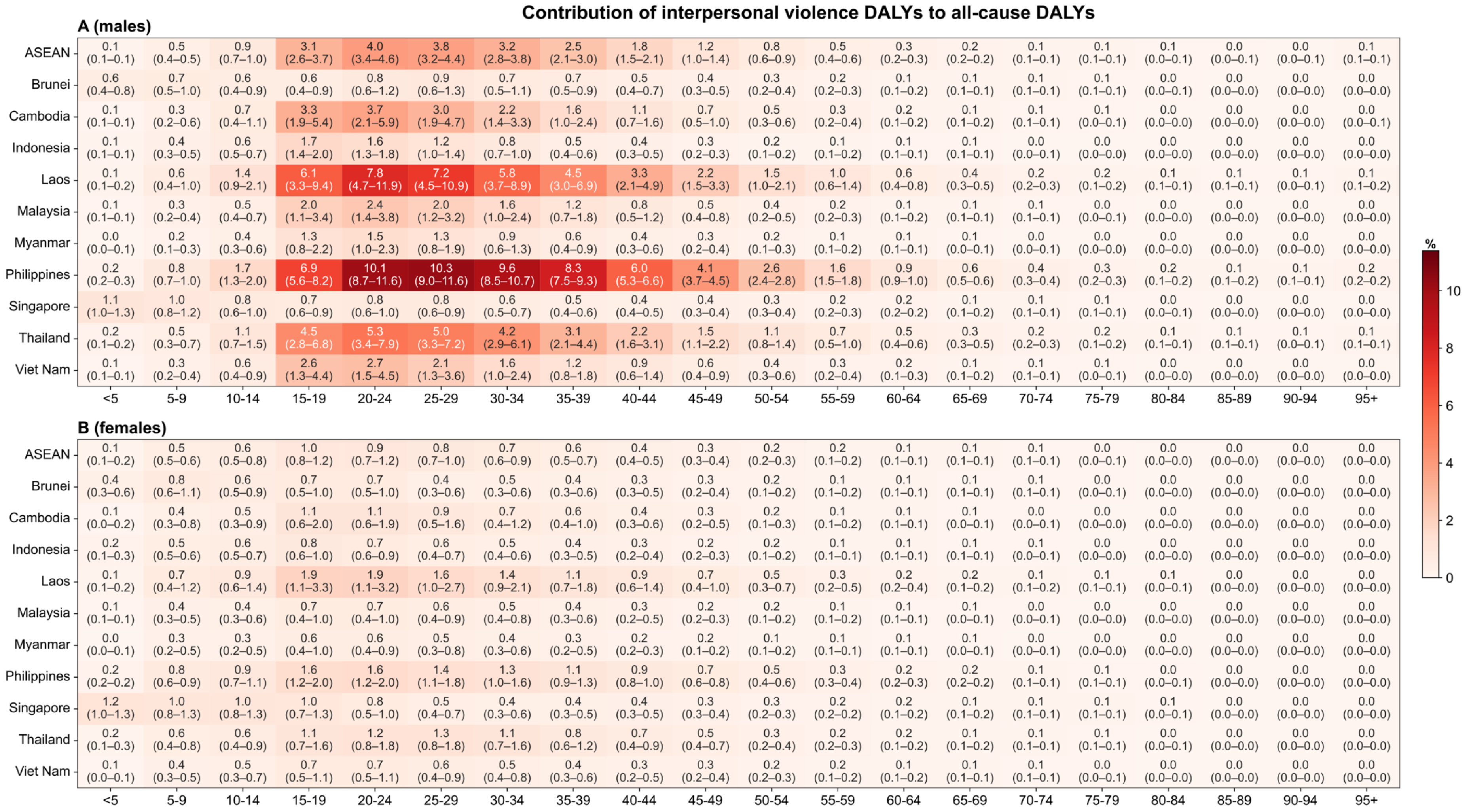

Figure S12 | Contribution (95% UI) of interpersonal violence DALYs to all-cause DALYs (in %) for **A**) males, and **B**) females, by country and age group (in years), 2021.

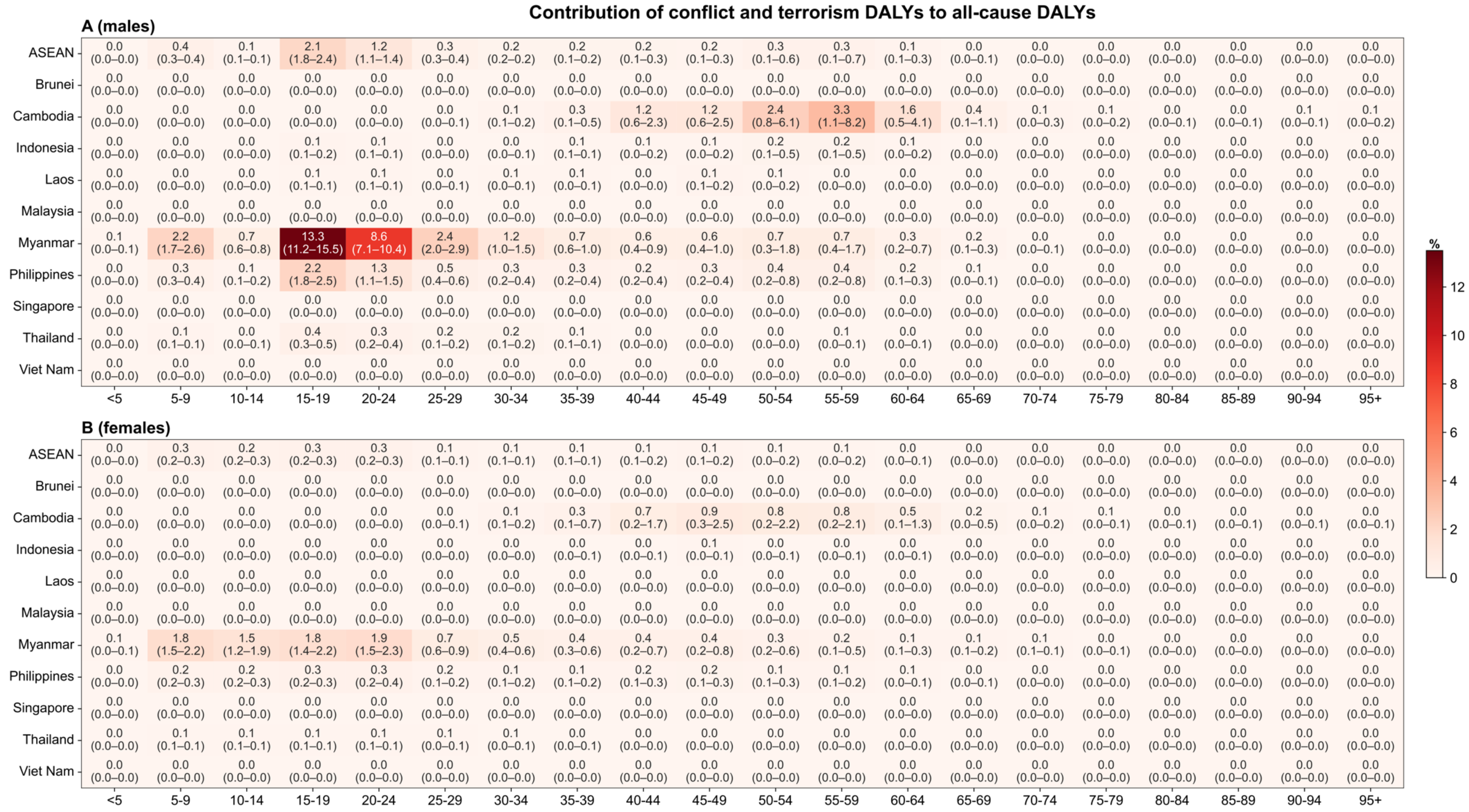

Figure S13 | Contribution (95% UI) of conflict and terrorism DALYs to all-cause DALYs (in %) for **A**) males, and **B**) females, by country and age group (in years), 2021.

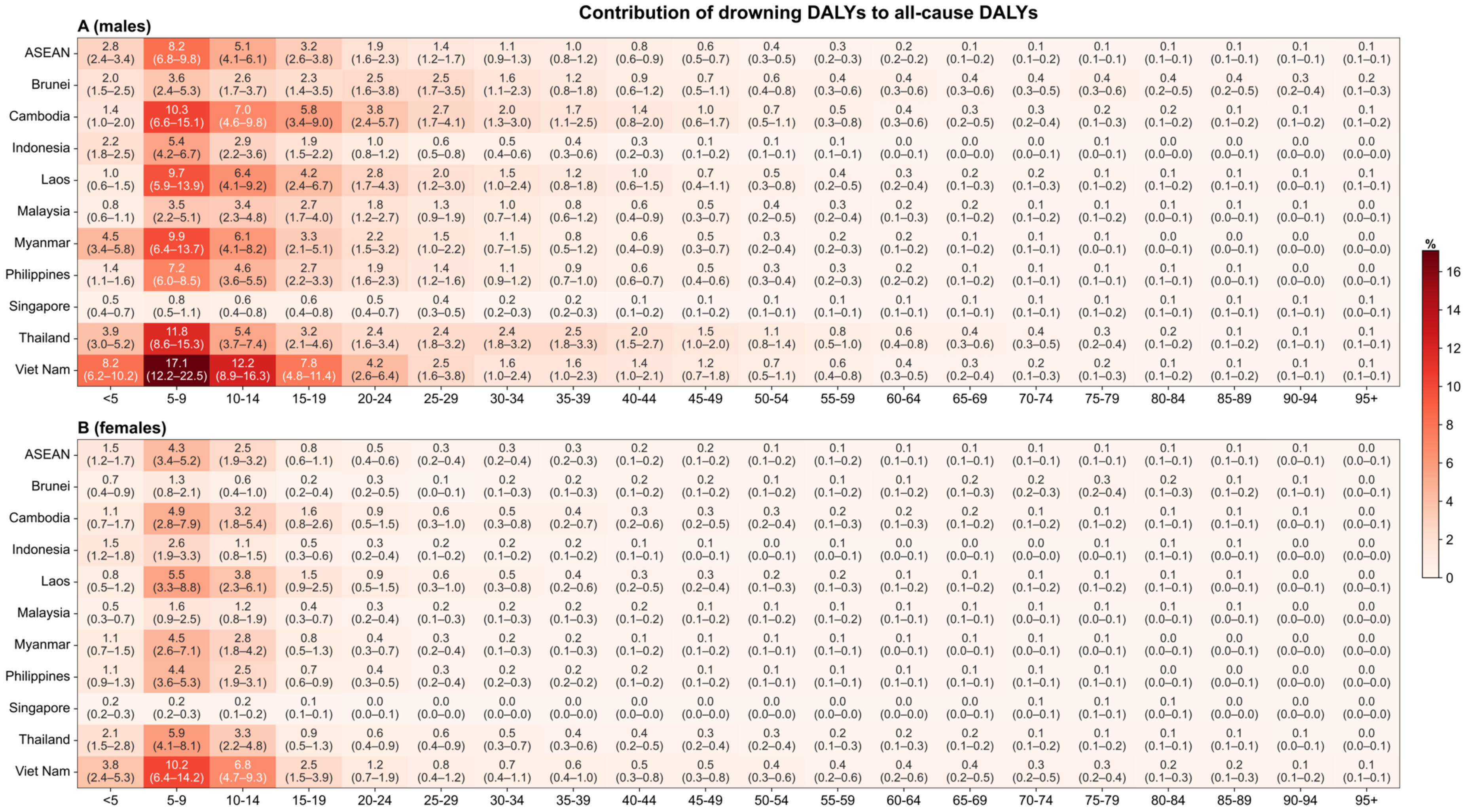

Figure S14 | Contribution (95% UI) of drowning DALYs to all-cause DALYs (in %) for **A**) males, and **B**) females, by country and age group (in years), 2021.

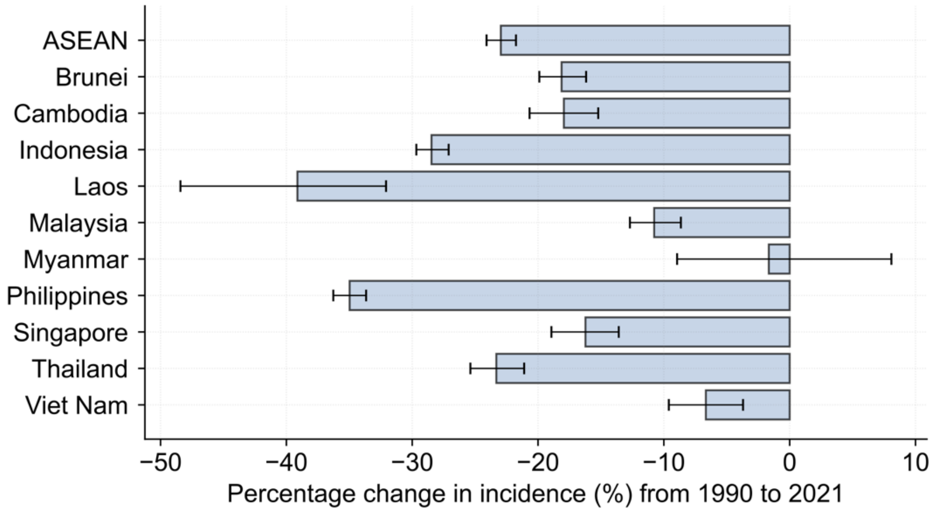

Figure S15 | Percentage change in age-standardised incidence rates of injuries from 1990 to 2021. The error bars represent the 95% uncertainty interval.

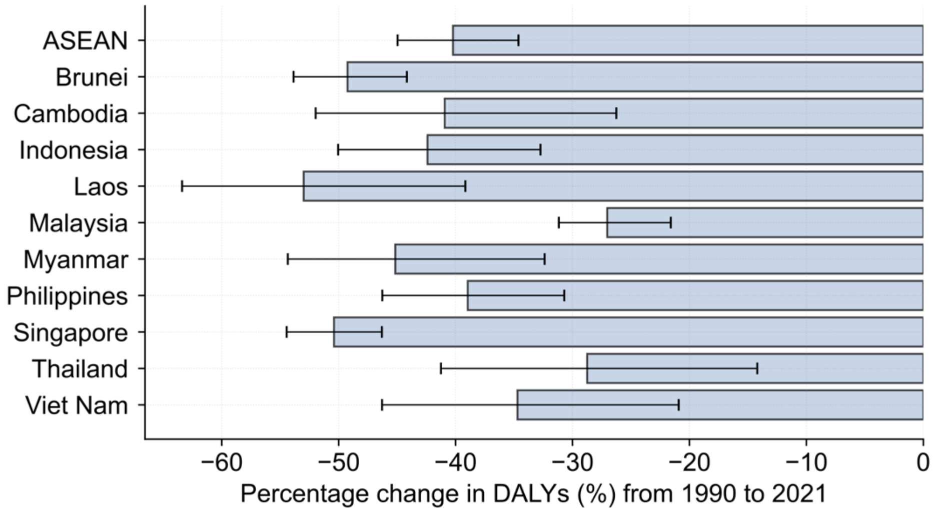

Figure S16 | Percentage change in age-standardised DALY rates associated with injuries from 1990 to 2021. The error bars represent the 95% uncertainty interval.

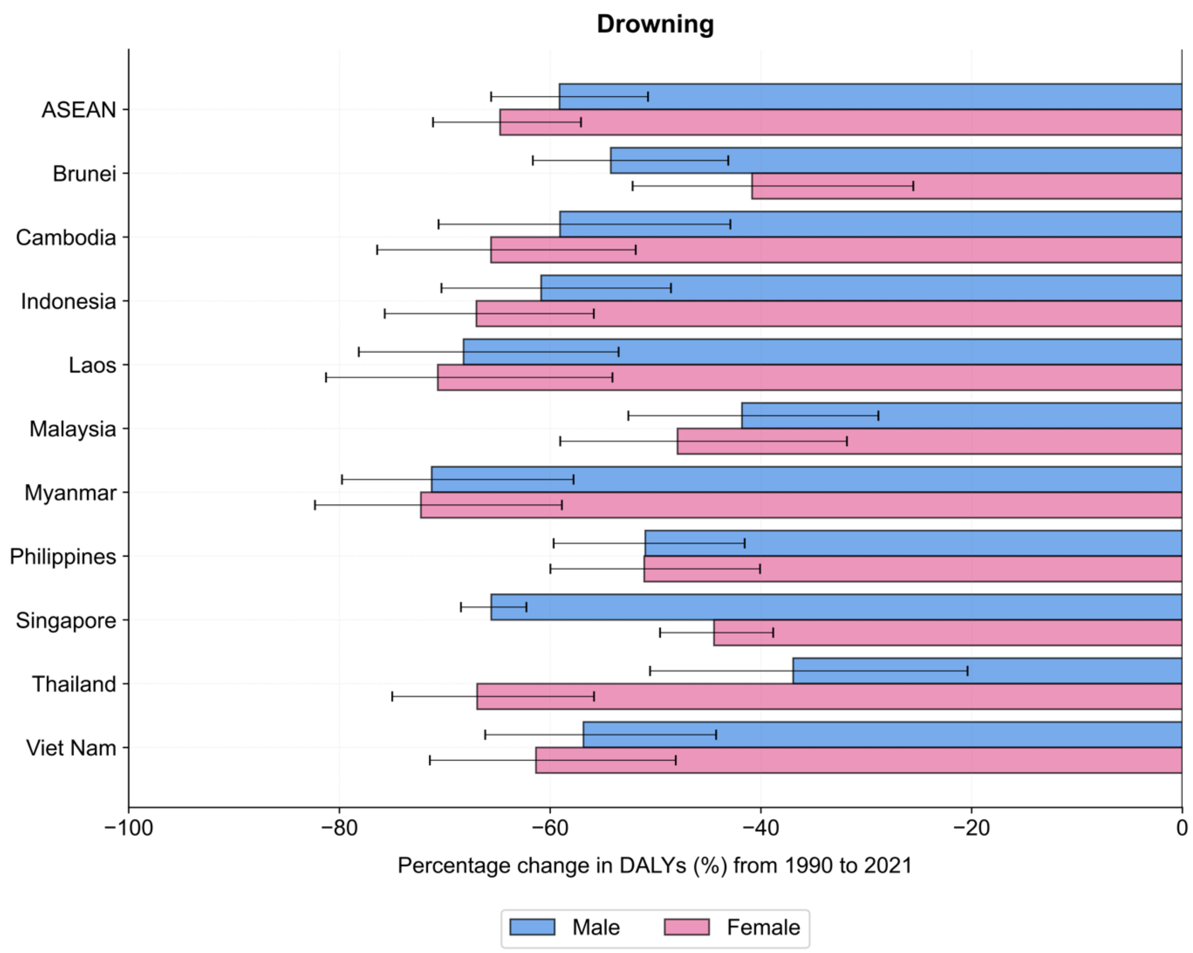

Figure S17 | Percentage change in age-standardised DALY rates associated with drowning injuries by sex from 1990 to 2021. The error bars represent the 95% uncertainty interval.

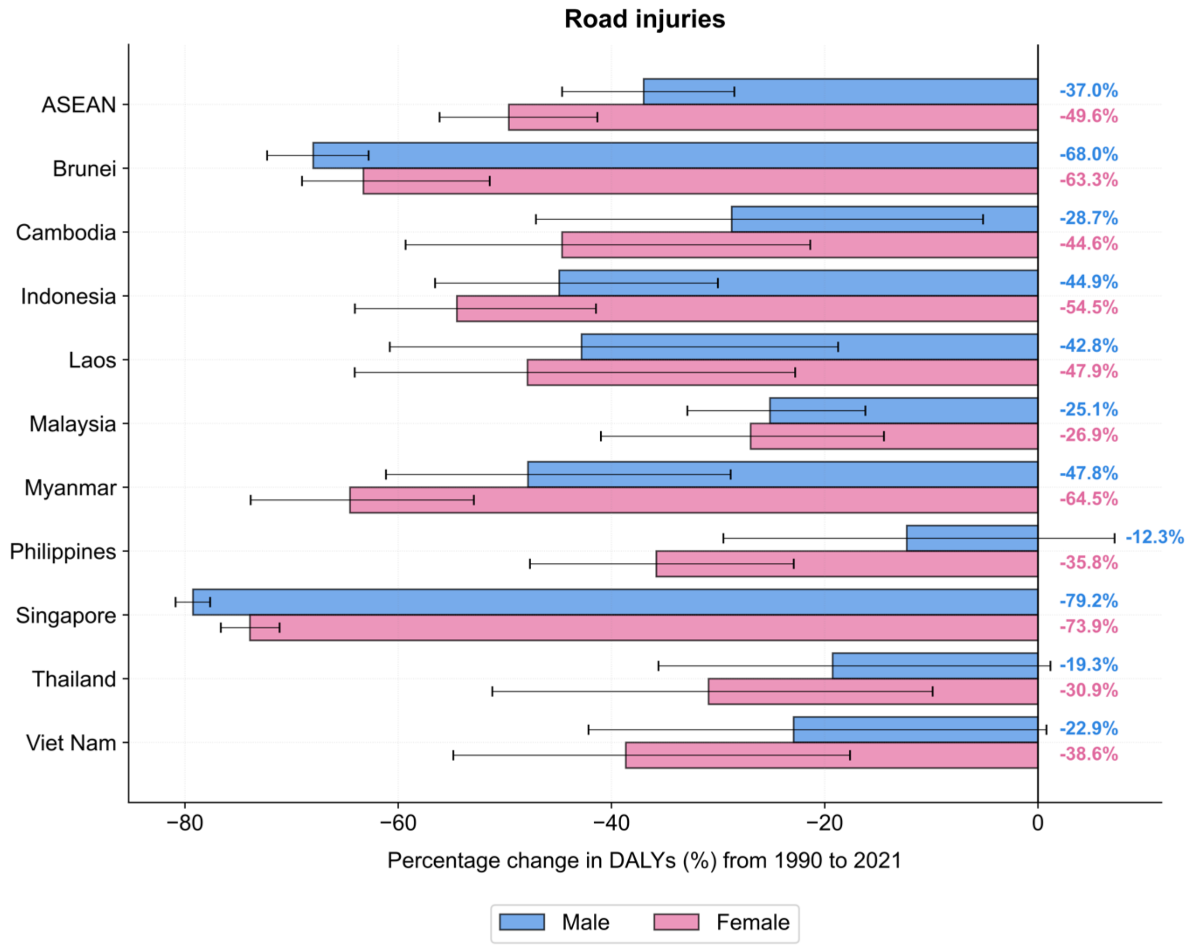

Figure S18 | Percentage change in age-standardised DALY rates associated with road injuries by sex from 1990 to 2021. The error bars represent the 95% uncertainty interval.

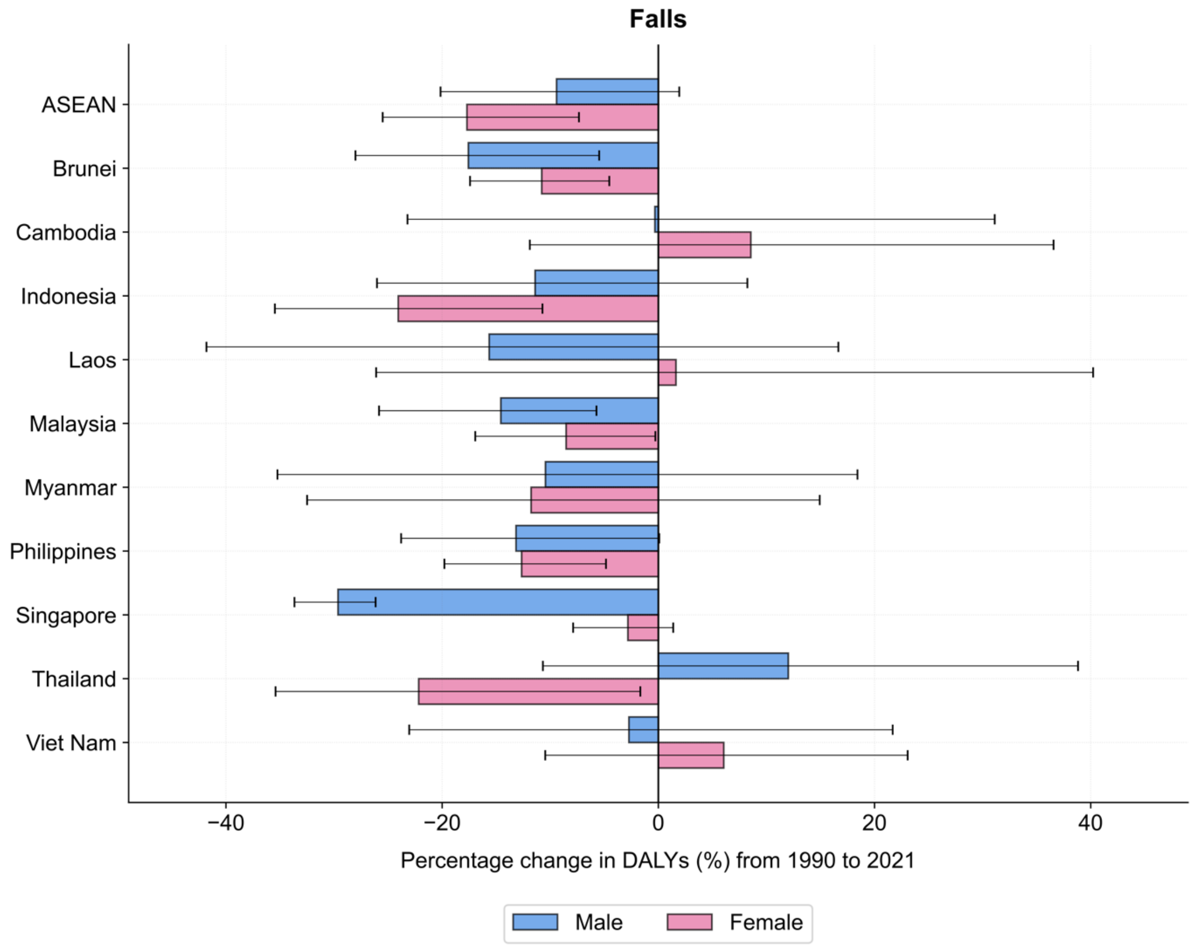

Figure S19 | Percentage change in age-standardised DALY rates associated with injuries attributable to falls, by sex from 1990 to 2021. The error bars represent the 95% uncertainty interval.

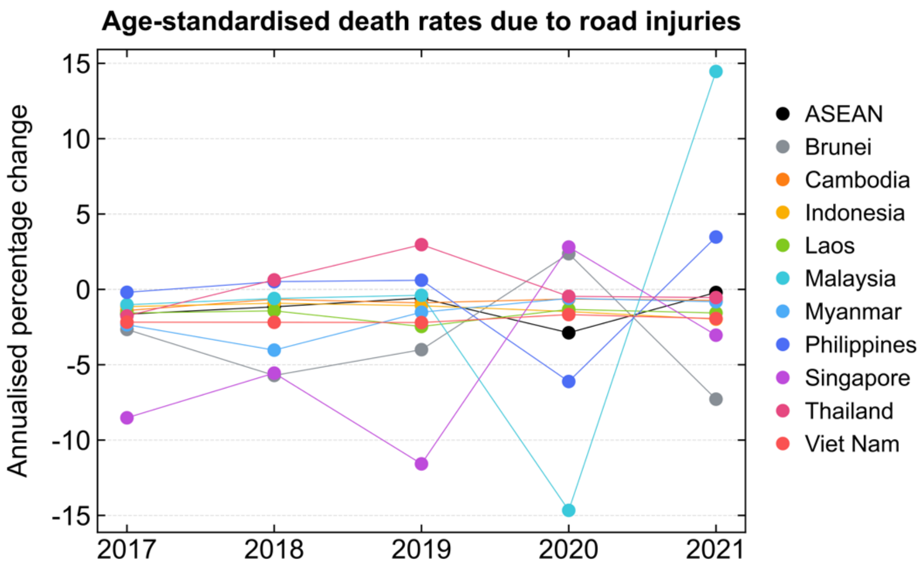

Figure S20 | Annualised percentage change in road injury death rates for ASEAN and its member states.

Table S6 | Relative contribution (%) of Level 3 cause-specific incidence cases to total injury incidence in ASEAN and by country, 2021.

	Relative contribution of incidence cases (in %)										
	ASEAN	Brunei	Cambodia	Indonesia	Laos	Malaysia	Myanmar	Philippines	Singapore	Thailand	Viet Nam
**Adverse effects of medical treatment**	0·44	0·52	0·47	0·28	0·56	0·69	0·34	0·58	0·61	0·53	0·58
**Animal contact**	8·10	4·31	9·02	7·52	9·56	6·50	7·75	12·71	4·76	4·61	8·03
**Conflict and terrorism**	2·09	0·00	0·02	0·13	0·07	0·01	13·27	2·54	0·00	0·36	0·00
**Drowning**	0·22	0·12	0·28	0·13	0·28	0·19	0·23	0·24	0·09	0·34	0·32
**Environmental heat and cold exposure**	0·45	0·98	0·53	0·52	0·36	0·63	0·29	0·53	1·25	0·36	0·30
**Exposure to forces of nature**	0·33	0·00	0·02	0·38	0·00	0·31	0·00	1·13	0·00	0·03	0·05
**Exposure to mechanical forces**	22·88	36·73	20·12	23·94	22·79	24·38	18·31	18·69	37·82	19·65	27·99
**Falls**	30·85	27·04	43·18	29·37	24·96	29·86	28·31	27·22	29·93	38·26	33·20
**Fire, heat, and hot substances**	1·75	1·99	1·73	1·91	2·06	2·44	0·82	1·98	1·85	1·18	2·01
**Foreign body**	4·70	5·13	3·58	5·94	4·30	4·38	2·50	5·74	4·96	3·69	3·68
**Interpersonal violence**	5·64	1·75	4·54	4·72	11·32	3·95	1·83	13·97	1·71	6·28	3·05
**Other transport injuries**	0·40	0·69	0·32	0·53	0·36	0·50	0·19	0·41	0·79	0·27	0·29
**Other unintentional injuries**	9·90	9·90	6·43	14·40	12·31	10·55	6·63	6·01	11·31	9·11	7·25
**Poisonings**	0·35	0·56	0·26	0·38	0·32	0·47	0·32	0·41	0·71	0·19	0·28
**Police conflict and executions**	1·72	0·00	0·14	0·09	0·07	0·28	13·30	0·21	0·05	0·08	0·06
**Road injuries**	9·51	9·91	8·76	9·26	9·82	14·04	5·55	7·04	3·49	14·02	11·91
**Self-harm**	0·67	0·36	0·59	0·50	0·87	0·82	0·36	0·59	0·70	1·06	1·01

Table S7 | Relative contribution (%) of Level 3 cause-specific deaths to total injury deaths in ASEAN and by country, 2021.

	Relative contribution of deaths (in %)										
	**ASEAN**	**Brunei**	**Cambodia**	**Indonesia**	**Laos**	**Malaysia**	**Myanmar**	**Philippines**	**Singapore**	**Thailand**	**Viet Nam**
**Adverse effects of medical treatment**	0·91	2·68	1·15	1·26	1·17	1·13	1·08	0·63	1·55	0·63	0·63
**Animal contact**	1·32	0·33	1·23	1·58	1·19	0·64	2·78	0·96	0·04	0·66	1·21
**Conflict and terrorism**	1·04	0·00	0·01	0·08	0·03	0·01	8·29	1·26	0·00	0·13	0·00
**Drowning**	9·29	12·16	12·88	6·99	12·24	5·58	11·38	9·68	3·84	9·08	11·66
**Environmental heat and cold exposure**	0·06	0·52	0·08	0·02	0·07	0·01	0·07	0·25	0·11	0·04	0·02
**Exposure to forces of nature**	0·37	0·00	0·01	0·53	0·00	0·35	0·00	1·18	0·00	0·03	0·05
**Exposure to mechanical forces**	2·20	2·66	2·27	1·84	2·18	1·04	2·46	1·22	2·08	1·11	4·42
**Falls**	17·79	15·41	24·59	21·11	12·76	10·74	19·60	10·16	22·45	12·51	22·82
**Fire, heat, and hot substances**	1·28	3·04	1·82	1·69	1·75	1·41	0·81	1·32	0·64	1·14	0·87
**Foreign body**	2·14	3·50	1·97	1·45	2·41	3·34	2·24	1·96	3·51	3·24	2·04
**Interpersonal violence**	7·87	3·25	5·16	2·80	15·50	3·69	2·30	30·40	3·41	7·20	2·50
**Other transport injuries**	2·81	4·74	2·40	3·68	2·17	5·84	1·97	2·50	0·60	2·19	2·12
**Other unintentional injuries**	3·99	2·35	5·57	4·50	5·36	2·01	6·39	3·05	0·90	3·04	3·76
**Poisonings**	1·00	0·74	0·75	1·04	0·73	1·28	2·09	0·32	0·15	0·36	1·44
**Police conflict and executions**	0·60	0·00	0·05	0·05	0·03	0·10	5·74	0·09	0·27	0·02	0·03
**Road injuries**	37·82	33·63	31·83	46·44	31·44	51·71	26·29	26·24	13·15	42·86	34·22
**Self-harm**	9·50	14·99	8·23	4·94	10·96	11·13	6·52	8·79	47·29	15·75	12·20

Table S8 | Relative contribution (%) of Level 3 cause-specific DALYs to total injury-related DALYs in ASEAN and by country, 2021.

	Relative contribution of DALYs (in %)										
	ASEAN	Brunei	Cambodia	Indonesia	Laos	Malaysia	Myanmar	Philippines	Singapore	Thailand	Viet Nam
**Adverse effects of medical treatment**	0·65	1·42	0·89	0·86	1·01	0·75	0·82	0·47	0·48	0·39	0·47
**Animal contact**	1·16	0·48	1·14	1·26	1·16	0·60	2·35	1·02	0·53	0·55	1·07
**Conflict and terrorism**	2·05	0·01	5·59	0·78	0·25	0·03	10·05	2·93	0·00	0·37	0·01
**Drowning**	9·74	7·11	12·76	7·70	12·53	5·34	12·69	9·65	1·33	7·92	13·67
**Environmental heat and cold exposure**	0·17	0·96	0·17	0·18	0·13	0·16	0·14	0·28	1·56	0·12	0·09
**Exposure to forces of nature**	1·21	0·01	0·16	1·48	0·10	0·38	3·07	1·95	0·00	0·16	0·24
**Exposure to mechanical forces**	3·88	7·99	3·33	4·00	3·40	2·93	3·78	2·44	11·70	2·51	6·34
**Falls**	15·59	22·68	20·34	17·69	11·45	11·46	16·47	10·44	33·25	13·56	17·78
**Fire, heat, and hot substances**	2·20	4·82	2·50	2·94	2·77	2·29	1·41	2·26	4·88	1·35	1·86
**Foreign body**	1·98	2·83	2·02	1·80	2·77	2·54	2·27	2·00	1·86	1·94	1·93
**Interpersonal violence**	9·08	3·63	6·26	4·76	16·27	5·22	2·88	28·61	3·63	8·53	3·80
**Other transport injuries**	2·56	3·50	2·14	3·29	1·98	5·25	1·65	2·24	1·54	2·01	2·06
**Other unintentional injuries**	5·37	4·48	6·06	6·86	6·48	3·56	6·34	3·69	5·63	4·32	4·79
**Poisonings**	0·90	0·98	0·68	0·98	0·69	1·20	1·62	0·34	0·97	0·35	1·29
**Police conflict and executions**	0·83	0·01	0·09	0·08	0·06	0·13	6·62	0·38	0·40	0·04	0·05
**Road injuries**	34·87	29·98	29·12	41·39	29·34	48·62	22·76	23·71	10·95	42·39	33·78
**Self-harm**	7·74	9·13	6·74	3·93	9·60	9·55	5·11	7·61	21·30	13·48	10·77

Table S9 | Age-standardised mortality rates and age-standardized DALY rates attributable to road injuries, falls, and self-harm by country and sex in 2021. These causes represent the top three Level 3 leading injury causes of mortality in ASEAN.

	Mortality rate per 100 000			DALY rate per 100 000		
	Road injuries	Falls	Self-harm	Road injuries	Falls	Self-harm
**ASEAN**						
Total	17.4 (15.5–19.4)	10.4 (8.0–11.9)	4.3 (3.7–4.9)	916.9 (821.6–1,014.0)	448.7 (380.8–519.1)	198.0 (171.0–224.0)
Male	27.5 (24.4–30.9)	11.8 (9.8–14.1)	6.6 (5.7–7.5)	1,438.7 (1,288.7–1,604.9)	559.7 (480.3–654.2)	299.8 (255.9–341.3)
Female	7.7 (6.7–9.1)	8.7 (5.4–10.8)	2.2 (1.8–2.7)	393.5 (342.7–454.9)	330.9 (265.6–393.6)	96.8 (80.4–125.6)
**Brunei**						
Total	9.1 (8.0–10.3)	6.1 (5.0–7.1)	3.7 (2.9–4.2)	606.1 (532.2–685.4)	513.8 (409.1–647.7)	166.8 (137.6–192.2)
Male	12.7 (11.0–14.6)	9.2 (7.5–11.3)	5.6 (4.5–6.5)	844.9 (729.4–961.1)	630.0 (513.8–775.0)	252.7 (203.5–295.6)
Female	5.2 (4.3–6.5)	3.7 (2.8–4.6)	1.6 (1.2–2.0)	338.7 (288.0–394.8)	392.3 (292.5–507.3)	70.1 (55.1–88.8)
**Cambodia**						
Total	18.7 (14.2–24.5)	22.5 (17.3–27.6)	4.9 (3.6–6.4)	967.4 (740.5–1,257.2)	835.1 (684.2–1,033.1)	223.9 (163.9–299.5)
Male	29.4 (22.1–38.3)	25.6 (19.3–33.4)	7.3 (5.4–9.7)	1,514.8 (1,161.6–1,965.7)	1,057.3 (833.6–1,353.7)	324.2 (236.0–434.1)
Female	9.2 (6.7–12.6)	19.2 (14.0–24.3)	2.8 (1.9–4.0)	448.0 (340.1–614.2)	620.7 (506.4–760.1)	132.5 (91.2–193.5)
**Indonesia**						
Total	15.8 (13.0–19.5)	10.8 (8.3–12.7)	1.7 (1.4–2.1)	840.3 (705.5–1,032.9)	421.6 (358.2–486.2)	77.9 (63.7–99.1)
Male	24.0 (18.8–30.9)	10.9 (8.5–13.5)	2.4 (1.9–3.1)	1,290.1 (1,045.6–1,644.3)	470.1 (392.4–572.7)	106.6 (83.9–142.4)
Female	7.5 (5.9–10.0)	10.2 (6.3–12.8)	1.1 (0.8–1.5)	377.3 (305.3–493.1)	361.1 (286.3–431.1)	48.6 (36.6–70.4)
**Laos**						
Total	16.2 (12.2–21.1)	10.0 (7.8–12.5)	5.5 (4.1–7.4)	869.8 (669.6–1,116.3)	422.6 (341.5–527.3)	278.4 (204.1–371.1)
Male	23.8 (17.9–30.9)	12.4 (9.2–16.6)	8.3 (6.0–11.6)	1,277.3 (987.4–1,645.4)	553.4 (434.0–708.7)	395.7 (283.1–538.2)
Female	8.8 (6.4–11.7)	7.6 (5.3–9.9)	2.9 (1.9–4.2)	460.8 (345.1–603.7)	289.2 (231.9–359.4)	161.4 (106.9–231.2)
**Malaysia**						
Total	23.7 (21.8–25.7)	6.2 (4.5–7.2)	5.1 (4.6–5.8)	1,140.1 (1,060.5–1,224.6)	301.1 (251.4–358.9)	220.0 (196.5–254.1)
Male	38.0 (34.7–41.6)	7.8 (5.8–9.5)	7.9 (7.0–9.0)	1,822.2 (1,672.4–1,971.1)	393.3 (324.7–465.4)	338.5 (298.2–392.3)
Female	8.8 (7.0–10.1)	4.6 (3.1–5.9)	2.1 (1.8–3.0)	412.6 (339.0–466.2)	204.6 (163.5–252.7)	92.5 (76.2–133.0)
**Myanmar**						
Total	14.5 (11.5–18.4)	14.0 (10.8–18.2)	3.6 (2.4–4.6)	784.7 (629.4–981.4)	636.4 (520.5–793.7)	175.4 (122.6–227.1)
Male	22.5 (17.7–28.7)	19.4 (14.7–28.5)	6.4 (4.2–8.3)	1,203.8 (956.3–1,511.3)	913.0 (726.4–1,212.4)	297.9 (199.8–389.6)
Female	7.5 (5.0–9.7)	9.6 (6.3–12.3)	1.2 (0.9–1.7)	397.4 (280.5–510.2)	390.3 (319.2–476.6)	64.5 (46.9–87.8)
**Philippines**						
Total	11.8 (9.9–13.8)	6.4 (5.0–7.4)	3.8 (3.2–4.6)	629.2 (544.3–725.2)	327.4 (273.1–390.4)	197.1 (159.2–234.4)
Male	18.6 (15.2–22.5)	7.9 (6.2–9.8)	6.3 (4.9–7.7)	999.9 (836.4–1,181.2)	423.0 (351.8–517.2)	312.6 (247.2–383.9)
Female	4.9 (4.0–6.0)	5.0 (3.6–6.1)	1.5 (1.1–1.9)	249.6 (207.9–300.3)	229.6 (188.6–281.5)	79.2 (60.7–100.6)
**Singapore**						
Total	1.9 (1.7–2.0)	2.7 (2.4–2.9)	6.6 (6.2–7.0)	148.5 (130.3–169.9)	388.3 (284.5–513.1)	313.1 (294.9–333.1)
Male	3.1 (2.8–3.2)	4.2 (3.8–4.5)	8.8 (8.3–9.4)	221.2 (197.3–248.8)	442.7 (338.3–569.4)	416.1 (390.8–445.6)
Female	0.8 (0.7–0.8)	1.4 (1.2–1.6)	4.4 (4.1–4.7)	75.8 (63.0–91.4)	334.3 (231.6–455.9)	211.0 (197.3–225.3)

**Thailand**						
Total	29.7	6.5	10.1	1,697.3	404.6	500.1
	(23.8–36.9)	(4.9–8.6)	(7.8–12.5)	(1,383.3–2,077.5)	(326.6–500.9)	(392.7–614.8)
Male	49.4	9.5	17.2	2,807.8	613.3	853.9
	(39.2–62.2)	(7.2–12.2)	(13.3–21.4)	(2,282.8–3,477.5)	(491.0–753.2)	(664.6–1,056.5)
Female	11.2	3.7	3.5	637.0	210.2	167.4
	(8.9–14.2)	(2.6–5.9)	(2.6–4.8)	(514.6–791.2)	(166.3–267.1)	(125.5–231.5)
**Viet Nam**						
Total	21.7	18.3	7.7	1,055.5	608.2	328.1
	(17.0–26.7)	(10.5–24.2)	(5.8–9.7)	(850.6–1,277.7)	(476.8–725.0)	(254.7–420.6)
Male	35.2	20.4	11.0	1,670.5	756.6	460.3
	(27.9–42.8)	(14.7–25.2)	(8.3–13.8)	(1,336.0–2,050.7)	(618.2–908.0)	(346.9–595.6)
Female	9.7	15.4	4.7	458.5	443.5	200.2
	(7.0–12.5)	(6.4–22.8)	(3.4–6.1)	(352.8–568.9)	(306.0–565.7)	(148.7–272.3)

95% UI=95% uncertainty interval.

Table S10 | Incidence rate of injuries per 100 000 by age and country, 2021.

	Incidence rate per 100 000										
	**ASEAN**	**Brunei**	**Cambodia**	**Indonesia**	**Laos**	**Malaysia**	**Myanmar**	**Philippines**	**Singapore**	**Thailand**	**Viet Nam**
**Age-standardised**											
Total	5 262·0	13 921·8	5 589·2	4 523·9	4 632·5	4 675·0	7 532·8	4 752·7	12 656·1	5 674·0	6 234·8
	(4 963·2–5 581·9)	(12 911·8–14 897·5)	(5 287·5–5 892·7)	(4 214·3–4 846·7)	(4 389·1–4 899·3)	(4 398·8–4 968·4)	(6 927·2–8 338·3)	(4 466·0–5 058·0)	(11 406·8–13 880·7)	(5 358·8–5 981·7)	(5 887·2–6 564·2)
Male	6 388·3	16 860·9	6 799·7	4 973·1	6 084·6	5 804·4	10 405·1	5 982·3	15 164·4	7 777·0	7 639·4
	(6 057·0–6 727·2)	(15 630·0–18 020·3)	(6 437·8–7 140·2)	(4 654·3–5 309·9)	(5 759·8–6 403·2)	(5 487·6–6 147·4)	(9 508·0–11 554·4)	(5 632·3–6 361·6)	(13 598·7–16 666·0)	(7 393·4–8 185·3)	(7 227·3–8 043·6)
Female	4 076·7	10 528·0	4 343·4	4 023·0	3 156·8	3 448·1	4 767·5	3 465·9	10 109·6	3 647·0	4 686·3
	(3 791·3–4 366·4)	(9 693·9–11 329·5)	(4 081·7–4 636·0)	(3 698·9–4 350·3)	(2 934·1–3 385·1)	(3 185·8–3 724·4)	(4 372·7–5 244·5)	(3 206·4–3 760·0)	(9 072·2–11 202·7)	(3 395·2–3 915·5)	(4 384·8–4 988·9)
**<5 years**											
Total	4 245·8	13 381·0	3 424·6	4 907·5	2 714·5	3 328·6	5 871·6	2 739·0	13 043·3	3 222·0	4 118·2
	(3 809·7–4 772·6)	(11 871·5–15 085·1)	(3 063·3–3 837·0)	(4 382·7–5 605·5)	(2 415·2–3 068·7)	(2 881·5–3 893·7)	(5 093·3–7 009·7)	(2 420·9–3 097·4)	(11 185·0–15 178·9)	(2 829·4–3 678·0)	(3 653·5–4 618·7)
Male	4 043·3	14 054·6	3 176·4	4 513·3	2 853·7	3 284·1	6 118·2	2 692·5	13 054·6	3 179·6	3 822·0
	(3 612·2–4 546·3)	(12 323·6–15 892·7)	(2 836·2–3 561·9)	(4 009·8–5 128·6)	(2 524·8–3 229·7)	(2 827·2–3 854·2)	(5 263·2–7 252·2)	(2 382·8–3 046·9)	(11 076·5–15 356·5)	(2 766·3–3 663·8)	(3 366·2–4 313·5)
Female	4 460·9	12 653·2	3 684·0	5 322·1	2 569·7	3 376·0	5 613·5	2 789·2	13 031·5	3 266·8	4 439·0
	(4 020·7–5 036·3)	(11 127·2–14 392·9)	(3 261·7–4 178·6)	(4 735·8–6 111·1)	(2 267·2–2 957·6)	(2 917·8–3 956·9)	(4 825·9–6 709·5)	(2 464·9–3 158·5)	(11 170·5–15 041·4)	(2 872·6–3 740·9)	(3 925·0–4 995·9)
**5-9 years**											
Total	4 501·0	12 465·0	4 312·5	4 195·4	3 561·5	3 613·3	7 793·9	3 910·1	14 029·8	3 698·7	4 433·2
	(3 785·0–5 321·8)	(10 036·6–15 542·4)	(3 633·8–5 075·7)	(3 456·3–5 071·2)	(2 983·6–4 248·7)	(2 870·7–4 470·3)	(6 271·5–9 888·2)	(3 268·2–4 620·7)	(10 839·4–17 549·8)	(3 032·4–4 502·0)	(3 638·7–5 345·8)
Male	4 687·6	13 336·0	4 396·4	4 219·7	3 950·1	3 765·1	9 073·4	4 034·3	15 121·2	3 913·7	4 478·0
	(3 940·0–5 540·2)	(10 489·9–16 757·7)	(3 693·7–5 211·2)	(3 493·4–5 091·7)	(3 331·9–4 727·5)	(3 003·5–4 738·8)	(7 304·1–11 613·3)	(3 367·9–4 748·9)	(11 470·5–19 602·8)	(3 210·0–4 770·0)	(3 649·5–5 432·6)
Female	4 303·2	11 529·5	4 224·3	4 170·0	3 158·3	3 452·0	6 468·2	3 776·8	12 927·4	3 471·1	4 384·2
	(3 588·2–5 106·9)	(9 164·0–14 324·3)	(3 524·5–5 045·1)	(3 382·1–5 068·0)	(2 558·6–3 794·4)	(2 743·0–4 289·4)	(5 224·3–8 186·5)	(3 137·5–4 535·2)	(10 062·8–16 457·6)	(2 760·3–4 247·5)	(3 546·4–5 344·7)
**10-14 years**											
Total	5 149·4	13 370·9	5 261·0	4 760·5	4 610·1	4 480·8	7 124·2	4 829·8	15 553·2	5 011·9	5 403·4
	(4 233·3–6 135·8)	(10 262·6–16 669·2)	(4 336·8–6 246·3)	(3 853·6–5 721·2)	(3 786·8–5 466·3)	(3 596·3–5 407·1)	(5 924·1–8 561·6)	(3 930·9–5 766·3)	(11 994·8–19 689·9)	(4 041·6–5 999·0)	(4 382·3–6 519·9)
Male	5 669·8	14 632·4	5 795·0	4 935·7	5 424·3	5 126·4	9 064·0	5 116·8	18 068·4	5 889·3	6 079·6
	(4 720·2–6 703·5)	(11 191·8–18 631·8)	(4 844·8–6 838·0)	(4 013·4–5 843·1)	(4 494·2–6 439·8)	(4 107·7–6 188·6)	(7 387·4–11 218·5)	(4 212·1–6 064·1)	(13 669·5–23 166·6)	(4 812·0–7 023·6)	(4 934·9–7 366·2)
Female	4 597·7	11 964·3	4 697·0	4 574·3	3 769·0	3 797·4	5 138·5	4 524·8	13 138·1	4 091·2	4 671·4
	(3 712·1–5 569·1)	(9 236·7–14 807·2)	(3 817·4–5 714·8)	(3 678·7–5 553·7)	(2 990·3–4 532·8)	(2 968·8–4 732·7)	(4 272·6–6 171·2)	(3 604·6–5 467·4)	(10 090·4–16 630·7)	(3 228·1–4 952·7)	(3 725·6–5 768·9)
**15-19 years**											
Total	7 173·7	16 286·7	7 442·5	5 587·9	6 253·2	6 041·5	15 311·5	6 173·0	17 889·1	7 976·5	7 542·3
	(6 238·6–8 230·2)	(13 378·5–19 916·4)	(6 529·9–8 488·3)	(4 682·0–6 539·1)	(5 419·8–7 139·2)	(5 202·6–6 995·5)	(12 258·2–19 325·1)	(5 290·8–7 166·9)	(14 524·9–22 259·2)	(7 043·7–9 052·3)	(6 517·5–8 716·9)
Male	9 571·5	19 247·3	9 809·5	6 759·9	8 550·5	8 103·8	23 999·6	7 859·8	22 136·6	11 446·3	10 213·2
	(8 415·9–10 836·0)	(15 489·0–23 844·6)	(8 588·7–11 061·0)	(5 717·7–7 842·0)	(7 485·8–9 697·9)	(7 065·1–9 318·7)	(19 050·3–30 438·2)	(6 785·4–9 078·5)	(17 634·5–28 039·2)	(10 111·1–12 890·4)	(8 850·8–11 748·4)
Female	4 652·3	12 879·1	4 957·0	4 339·7	3 909·8	3 871·1	6 622·7	4 400·7	12 968·2	4 437·9	4 694·9
	(3 889·4–5 519·2)	(10 642·2–15 602·5)	(4 166·6–5 858·5)	(3 532·7–5 262·0)	(3 258·3–4 680·7)	(3 210·5–4 681·7)	(5 409·0–8 218·3)	(3 636·1–5 264·4)	(10 499·2–16 144·9)	(3 700·5–5 246·2)	(3 890·8–5 626·0)
**20-24 years**											
Total	7 320·4	19 572·8	8 200·6	5 720·6	7 119·0	6 588·8	12 861·0	6 716·1	18 734·3	9 192·0	8 281·5
	(6 471·1–8 306·4)	(16 792·6–23 142·3)	(7 198·2–9 360·9)	(4 904·7–6 720·4)	(6 372·2–8 079·2)	(5 829·5–7 539·4)	(11 070·4–15 065·7)	(5 881·8–7 782·1)	(15 593·7–23 122·1)	(8 185·5–10 331·4)	(7 321·8–9 460·2)
Male	10 237·5	25 472·6	11 613·9	7 423·2	10 495·4	9 282·7	20 017·8	9 236·9	25 069·8	14 122·8	11 896·6
	(9 212·6–11 445·0)	(21 915·7–29 983·3)	(10 279·9–13 029·2)	(6 441·3–8 671·8)	(9 445·6–11 726·7)	(8 296·5–10 427·4)	(17 273·4–23 262·5)	(8 123·8–10 580·1)	(20 810·6–30 814·1)	(12 676·6–15 736·6)	(10 480·4–13 466·4)
Female	4 290·3	12 606·5	4 685·6	3 921·6	3 726·1	3 708·9	5 933·4	4 097·1	12 431·3	4 298·9	4 471·0
	(3 612·7–5 136·4)	(10 596·6–15 293·6)	(3 947·4–5 618·1)	(3 211·5–4 803·8)	(3 169·6–4 470·7)	(3 064·6–4 499·5)	(4 950·5–7 127·6)	(3 438·2–4 998·8)	(10 118·4–15 545·8)	(3 629·7–5 248·2)	(3 778·5–5 424·1)
**25-29 years**											
Total	6 292·1	18 728·9	6 786·5	4 969·0	6 349·0	5 774·7	9 159·9	6 396·6	16 204·9	8 444·8	6 806·2

	(5 518·5–7 325·0)	(16 383·1–22 005·3)	(5 968·9–7 794·4)	(4 198·3–6 019·3)	(5 612·6–7 265·8)	(5 067·7–6 750·2)	(7 839·6–10 613·7)	(5 548·6–7 539·8)	(13 507·7–19 765·5)	(7 600·2–9 584·7)	(5 914·8–7 899·4)
Male	8 713·0	25 696·2	9 484·6	6 355·6	9 330·6	7 997·6	13 728·3	9 062·3	22 434·8	13 415·4	9 508·5
	(7 758·0–9 965·7)	(22 376·0–30 329·6)	(8 397·5–10 757·7)	(5 436·7–7 563·7)	(8 311·5–10 558·2)	(7 111·3–9 175·3)	(11 765·7–15 730·9)	(7 941·5–10 571·5)	(18 594·0–27 507·1)	(12 165·9–14 878·7)	(8 337·6–10 935·9)
Female	3 805·2	10 671·5	4 045·7	3 514·5	3 323·3	3 306·2	4 855·1	3 645·4	10 809·3	3 843·3	3 976·6
	(3 175·0–4 731·5)	(8 877·1–13 195·5)	(3 377·0–4 908·0)	(2 863·0–4 463·7)	(2 775·4–4 056·3)	(2 695·4–4 166·6)	(3 918·1–5 922·9)	(2 988·1–4 598·6)	(8 660·7–13 909·0)	(3 203·0–4 768·8)	(3 301·6–4 839·6)
**30-34 years**											
Total	5 514·7	16 142·1	5 718·1	4 400·5	5 453·9	5 145·1	6 726·9	5 898·1	13 694·5	7 677·5	5 996·0
	(4 698·3–6 336·0)	(13 532·4–18 992·6)	(4 903·1–6 558·7)	(3 606·8–5 299·0)	(4 700·8–6 220·4)	(4 357·6–6 016·7)	(5 784·8–7 735·7)	(4 979·3–6 800·7)	(11 074·5–17 201·9)	(6 785·5–8 652·5)	(5 080·3–6 919·2)
Male	7 540·3	21 939·1	7 908·1	5 473·1	7 802·8	6 989·9	10 403·9	8 319·6	18 016·9	12 097·9	8 238·1
	(6 551·1–8 546·9)	(18 339·3–25 783·0)	(6 820·7–8 966·7)	(4 583·0–6 459·6)	(6 748·5–8 740·8)	(6 030·7–8 141·5)	(8 967·9–11 954·3)	(7 100·4–9 503·0)	(14 532·5–22 762·1)	(10 789·3–13 504·7)	(7 121·9–9 486·3)
Female	3 430·7	9 264·2	3 532·7	3 289·4	3 036·6	3 023·9	3 290·3	3 339·9	9 360·5	3 555·2	3 652·7
	(2 769·0–4 222·7)	(7 535·2–11 482·4)	(2 881·3–4 251·4)	(2 618·1–4 120·9)	(2 475·0–3 722·0)	(2 374·2–3 863·2)	(2 679·0–3 959·6)	(2 676·1–4 098·0)	(7 192·5–12 102·1)	(2 895·0–4 364·8)	(2 998·9–4 453·1)
**35-39 years**											
Total	5 010·1	14 231·1	5 230·3	3 923·9	4 853·4	4 554·9	5 732·5	5 326·7	11 594·4	6 534·7	5 996·2
	(4 248·1–5 954·5)	(11 903·4–17 347·9)	(4 498·2–6 078·9)	(3 223·1–4 825·4)	(4 189·8–5 749·3)	(3 868·0–5 480·9)	(4 973·3–6 684·5)	(4 509·1–6 270·0)	(9 189·5–14 532·3)	(5 717·7–7 454·1)	(5 112·0–7 050·2)
Male	6 786·9	18 925·4	7 252·1	4 762·3	6 825·3	6 070·9	8 635·3	7 456·2	14 666·4	9 973·8	8 453·4
	(5 915·5–7 861·5)	(15 749·4–23 110·8)	(6 262·8–8 396·5)	(3 981·4–5 703·9)	(5 934·1–7 971·6)	(5 246·6–7 137·5)	(7 471·9–10 028·2)	(6 313·3–8 722·0)	(11 608·1–18 447·4)	(8 818·4–11 197·3)	(7 271·7–9 861·8)
Female	3 194·8	8 486·1	3 255·6	3 065·0	2 832·5	2 801·6	3 018·6	3 068·9	8 283·7	3 296·8	3 458·6
	(2 582·0–3 971·2)	(6 820·1–10 509·1)	(2 640·9–3 940·6)	(2 400·4–3 938·7)	(2 319·5–3 517·4)	(2 205·4–3 640·3)	(2 504·8–3 681·2)	(2 497·5–3 781·8)	(6 283·2–10 839·9)	(2 676·3–4 066·2)	(2 805·0–4 211·2)
**40-44 years**											
Total	4 485·2	12 882·5	4 741·8	3 493·5	4 359·4	4 041·0	4 907·9	4 676·8	9 749·0	5 442·8	5 852·8
	(3 826·9–5 287·6)	(11 021·1–15 369·0)	(4 106·1–5 541·3)	(2 889·0–4 240·6)	(3 785·0–5 089·0)	(3 464·8–4 817·9)	(4 237·3–5 768·7)	(3 962·9–5 548·3)	(7 796·5–12 261·9)	(4 785·0–6 194·4)	(5 032·6–6 855·9)
Male	5 983·7	17 128·6	6 547·0	4 152·4	6 079·2	5 343·0	7 200·2	6 498·9	11 902·0	7 983·6	8 263·3
	(5 212·1–6 949·1)	(14 692·2–20 498·9)	(5 682·8–7 591·9)	(3 470·1–4 978·8)	(5 334·9–7 039·8)	(4 632·8–6 190·8)	(6 253·4–8 437·1)	(5 563·5–7 670·9)	(9 474·0–15 269·1)	(7 149·5–8 960·3)	(7 196·8–9 624·9)
Female	2 969·6	7 815·2	3 062·4	2 826·4	2 599·5	2 573·5	2 804·8	2 749·8	7 381·2	2 985·9	3 398·0
	(2 410·2–3 616·4)	(6 343·6–9 499·7)	(2 556·0–3 676·6)	(2 253·9–3 501·8)	(2 144·8–3 137·8)	(2 021·5–3 252·8)	(2 297·0–3 377·4)	(2 203·6–3 370·6)	(5 698·0–9 371·7)	(2 429·2–3 668·2)	(2 788·5–4 096·3)
**45-49 years**											
Total	4 184·0	11 636·8	4 507·7	3 324·6	3 970·0	3 824·5	4 656·6	4 157·8	8 647·6	4 673·3	5 768·3
	(3 576·6–4 855·4)	(9 918·5–13 820·5)	(3 884·1–5 189·3)	(2 765·5–3 991·7)	(3 472·3–4 513·5)	(3 314·4–4 480·3)	(4 035·7–5 379·5)	(3 485·0–4 992·5)	(6 963·6–10 727·1)	(4 066·2–5 382·0)	(4 969·2–6 711·3)
Male	5 434·5	15 138·7	5 943·4	3 892·9	5 449·5	4 968·9	6 576·8	5 678·5	10 075·1	6 477·6	7 947·3
	(4 704·9–6 278·5)	(12 856·4–17 977·5)	(5 160·7–6 777·1)	(3 261·8–4 642·5)	(4 829·2–6 231·4)	(4 363·7–5 798·1)	(5 667·2–7 557·6)	(4 741·3–6 766·4)	(8 100·6–12 682·9)	(5 717·5–7 392·4)	(6 892·9–9 144·6)
Female	2 939·3	7 777·1	3 209·2	2 753·6	2 493·3	2 590·8	2 955·8	2 566·4	6 977·3	2 976·4	3 570·0
	(2 429·4–3 566·1)	(6 331·0–9 557·6)	(2 695·3–3 841·3)	(2 225·5–3 371·2)	(2 068·1–2 947·8)	(2 119·5–3 158·8)	(2 486·1–3 499·9)	(2 059·1–3 147·1)	(5 519·7–8 852·7)	(2 434·0–3 565·2)	(2 952·0–4 302·3)
**50-54 years**											
Total	4 117·7	10 580·1	4 630·5	3 305·8	3 788·8	3 850·9	4 716·6	3 836·3	7 755·7	4 370·8	5 810·3
	(3 509·3–4 816·9)	(8 865·7–12 744·7)	(3 989·9–5 363·9)	(2 715·2–4 006·4)	(3 287·3–4 371·9)	(3 262·9–4 501·6)	(4 105·6–5 444·8)	(3 230·0–4 499·2)	(6 157·4–9 788·2)	(3 785·5–5 108·2)	(4 994·3–6 726·8)
Male	5 217·9	12 854·2	5 817·0	3 829·2	5 093·7	4 913·9	6 486·1	5 107·6	8 655·6	5 762·6	7 838·0
	(4 503·5–6 001·5)	(10 748·1–15 703·3)	(4 994·3–6 736·7)	(3 218·5–4 535·6)	(4 450·9–5 840·2)	(4 257·0–5 648·0)	(5 573·7–7 532·6)	(4 292·5–6 024·9)	(6 816·0–11 040·1)	(4 988·4–6 626·7)	(6 753·8–9 054·4)
Female	3 045·0	8 260·7	3 628·9	2 781·7	2 533·3	2 745·7	3 205·3	2 539·9	6 698·9	3 094·4	3 836·5
	(2 517·1–3 688·8)	(6 785·9–10 093·5)	(3 054·7–4 347·4)	(2 228·5–3 431·5)	(2 112·6–3 023·9)	(2 230·8–3 361·4)	(2 754·1–3 797·9)	(2 040·2–3 121·9)	(5 234·1–8 488·8)	(2 561·3–3 791·8)	(3 199·5–4 592·7)
**55-59 years**											
Total	4 135·7	9 979·6	4 834·7	3 403·9	3 708·6	3 943·0	4 804·3	3 678·0	6 986·3	4 118·1	5 819·0
	(3 589·1–4 784·5)	(8 527·7–11 888·7)	(4 171·0–5 537·5)	(2 869·6–4 045·0)	(3 264·4–4 223·4)	(3 449·1–4 519·1)	(4 188·6–5 492·5)	(3 099·7–4 316·0)	(5 778·8–8 678·5)	(3 616·7–4 688·4)	(5 065·1–6 727·3)
Male	5 068·9	11 793·3	5 867·3	3 758·7	4 870·1	4 942·2	6 603·4	4 790·7	7 725·7	5 216·8	7 686·0
	(4 457·2–5 819·5)	(9 881·3–14 181·8)	(5 040·3–6 796·8)	(3 214·1–4 417·2)	(4 300·3–5 572·0)	(4 334·1–5 646·1)	(5 694·2–7 619·7)	(4 029·8–5 691·6)	(6 244·5–9 632·2)	(4 567·5–5 954·1)	(6 707·9–8 835·9)
Female	3 251·1	8 128·6	3 917·8	3 051·8	2 584·1	2 930·3	3 322·4	2 584·6	6 194·5	3 127·7	4 137·1
	(2 781·7–3 854·5)	(6 965·7–9 671·1)	(3 349·5–4 532·5)	(2 492·7–3 683·6)	(2 226·0–3 040·4)	(2 487·6–3 504·0)	(2 869·1–3 838·6)	(2 155·2–3 099·2)	(5 048·5–7 790·0)	(2 664·1–3 711·1)	(3 548·1–4 848·1)
**60-64 years**											

Total	4 279·2	9 738·6	5 303·3	3 654·7	3 624·2	4 243·0	4 825·8	3 554·6	6 594·8	4 069·2	6 020·4
	(3 720·7–4 908·1)	(8 499·0–11 179·8)	(4 667·5–6 034·6)	(3 025·8–4 388·7)	(3 217·3–4 080·1)	(3 733·8–4 792·2)	(4 225·5–5 490·4)	(2 988·6–4 167·9)	(5 524·9–8 151·6)	(3 586·8–4 615·6)	(5 203·9–6 918·8)
Male	4 890·2	10 891·1	6 000·5	3 706·8	4 474·6	5 060·6	6 310·1	4 443·9	7 203·4	4 809·3	7 409·6
	(4 300·5–5 553·3)	(9 379·5–12 613·8)	(5 221·7–6 844·7)	(3 138·2–4 335·5)	(3 979·0–5 032·5)	(4 453·4–5 707·8)	(5 434·3–7 274·7)	(3 769·9–5 195·1)	(5 971·0–8 902·1)	(4 213·8–5 474·3)	(6 468·9–8 439·8)
Female	3 716·7	8 615·2	4 792·8	3 603·1	2 789·7	3 438·5	3 642·3	2 725·8	5 924·3	3 415·0	4 847·4
	(3 171·0–4 318·8)	(7 467·2–10 067·2)	(4 122·7–5 534·8)	(2 903·9–4 435·2)	(2 421·9–3 257·4)	(2 998·9–3 956·2)	(3 159·4–4 192·8)	(2 268·2–3 241·8)	(4 828·0–7 362·3)	(2 972·7–3 923·7)	(4 065·8–5 697·4)
**65-69 years**											
Total	4 362·8	9 483·7	5 487·1	3 870·1	3 485·9	4 456·2	4 782·6	3 472·0	6 180·6	4 003·5	6 151·4
	(3 756·1–5 050·6)	(8 391·8–10 843·6)	(4 702·1–6 381·6)	(3 172·3–4 740·4)	(3 087·4–3 923·6)	(3 892·6–5 027·3)	(4 164·8–5 460·1)	(2 902·8–4 092·9)	(5 167·9–7 510·4)	(3 507·7–4 608·3)	(5 228·5–7 201·1)
Male	4 658·1	9 780·3	5 671·3	3 683·9	4 069·5	5 098·9	5 861·5	4 143·0	6 583·3	4 583·7	6 842·2
	(4 049·2–5 342·9)	(8 484·5–11 342·9)	(4 871·8–6 529·6)	(3 109·5–4 409·1)	(3 619·6–4 618·1)	(4 471·5–5 754·2)	(5 063·1–6 792·5)	(3 450·1–4 874·3)	(5 455·0–8 062·7)	(4 018·7–5 262·6)	(5 851·6–7 930·5)
Female	4 103·7	9 199·9	5 369·1	4 050·7	2 919·7	3 838·3	3 964·6	2 898·2	5 761·0	3 507·5	5 619·7
	(3 466·5–4 869·0)	(8 044·1–10 548·4)	(4 516·1–6 308·6)	(3 231·7–5 049·3)	(2 502·6–3 366·1)	(3 273·0–4 439·4)	(3 385·8–4 580·9)	(2 429·8–3 460·1)	(4 728·5–6 933·5)	(2 998·0–4 075·5)	(4 680·3–6 699·6)
**70-74 years**											
Total	4 683·4	9 934·4	5 797·7	4 436·5	3 569·6	4 884·4	4 816·4	3 603·8	6 415·7	3 940·6	6 822·9
	(3 945·6–5 416·5)	(8 774·9–11 192·9)	(5 003·6–6 597·4)	(3 487·8–5 364·4)	(3 171·8–4 030·1)	(4 304·1–5 558·6)	(4 226·1–5 394·3)	(2 951·0–4 225·7)	(5 460·3–7 548·4)	(3 439·8–4 470·9)	(5 595·5–8 025·3)
Male	4 700·4	9 558·0	5 745·3	3 895·1	4 033·9	5 444·7	5 378·4	4 102·7	6 718·6	4 398·9	7 002·3
	(4 056·4–5 361·7)	(8 387·3–10 820·4)	(5 029·8–6 621·8)	(3 210·8–4 591·5)	(3 579·0–4 496·1)	(4 779·9–6 161·4)	(4 691·2–6 115·9)	(3 367·3–4 800·5)	(5 755·4–7 961·5)	(3 857·3–4 951·9)	(5 888·0–8 170·5)
Female	4 669·2	10 283·2	5 830·3	4 935·1	3 155·6	4 346·4	4 409·6	3 224·2	6 110·3	3 556·6	6 692·0
	(3 841·6–5 478·8)	(9 064·3–11 665·9)	(4 906·0–6 828·3)	(3 771·1–6 105·1)	(2 728·9–3 642·7)	(3 755·5–4 999·1)	(3 813·4–5 038·0)	(2 595·0–3 814·2)	(5 125·8–7 243·6)	(3 067·1–4 092·4)	(5 331·0–7 923·1)
**75-79 years**											
Total	5 826·3	10 530·8	7 418·6	5 653·9	4 016·4	5 703·4	5 903·6	3 963·1	7 663·9	4 324·3	9 382·3
	(5 011·2–6 769·8)	(9 507·6–11 706·8)	(6 456·6–8 601·1)	(4 548·0–6 886·1)	(3 626·5–4 491·0)	(5 098·6–6 446·9)	(5 270·2–6 617·0)	(3 290·3–4 693·3)	(6 853·7–8 615·4)	(3 865·6–4 940·9)	(7 851·3–11 200·4)
Male	4 878·5	10 727·2	5 999·8	4 130·4	3 988·7	5 665·5	5 429·5	3 948·5	7 806·4	4 242·1	7 477·7
	(4 301·9–5 564·7)	(9 652·6–11 980·2)	(5 294·1–6 914·7)	(3 479·5–4 859·6)	(3 588·7–4 425·4)	(5 073·0–6 395·5)	(4 797·3–6 168·7)	(3 353·1–4 613·7)	(6 913·0–8 758·7)	(3 766·8–4 803·5)	(6 354·2–8 718·1)
Female	6 534·2	10 335·4	8 264·5	6 911·2	4 040·1	5 738·9	6 215·2	3 972·3	7 547·3	4 388·7	10 590·6
	(5 545·0–7 714·1)	(9 142·6–11 554·3)	(6 987·3–9 666·8)	(5 373·6–8 639·9)	(3 543·4–4 576·1)	(4 963·2–6 637·6)	(5 478·1–7 111·6)	(3 248·4–4 801·9)	(6 626·9–8 653·4)	(3 841·2–5 146·8)	(8 667·8–12 962·8)
**80-84 years**											
Total	7 598·0	12 787·6	9 824·5	7 384·5	4 580·0	6 405·3	7 810·9	4 624·9	8 166·6	4 730·0	14 264·1
	(6 446·1–8 823·9)	(11 643·6–14 122·6)	(8 611·0–11 143·8)	(5 867·6–9 114·8)	(4 093·9–5 120·5)	(5 653·4–7 287·0)	(6 985·8–8 665·9)	(3 765·9–5 545·0)	(7 322·1–9 074·8)	(4 126·3–5 336·3)	(11 924·4–17 026·9)
Male	4 988·3	13 499·6	6 286·1	4 456·1	3 813·4	5 381·0	6 072·6	3 443·0	8 189·5	3 830·5	8 219·9
	(4 341·7–5 740·7)	(12 268·2–14 796·6)	(5 485·3–7 149·3)	(3 686·9–5 345·1)	(3 430·7–4 313·3)	(4 783·6–6 053·0)	(5 400·1–6 870·6)	(2 885·8–4 079·5)	(7 293·7–9 177·5)	(3 364·8–4 362·6)	(6 942·8–9 692·5)
Female	9 302·2	12 149·7	11 731·6	9 561·6	5 162·6	7 321·2	8 862·4	5 258·2	8 150·3	5 375·3	17 257·2
	(7 817·3–10 973·4)	(10 867·0–13 632·2)	(10 146·7–13 463·8)	(7 473·9–11 950·6)	(4 477·8–5 890·0)	(6 373·6–8 534·8)	(7 841·4–9 999·2)	(4 190·4–6 390·6)	(7 176·9–9 172·6)	(4 639·0–6 255·4)	(14 377·5–20 704·4)
**85-89 years**											
Total	9 416·4	16 565·5	11 927·3	9 274·7	5 067·6	6 797·9	9 447·5	5 633·7	7 630·2	4 926·8	19 404·7
	(7 726·7–11 140·7)	(14 981·2–18 556·4)	(10 042·8–14 048·5)	(7 153·4–11 823·0)	(4 400·8–5 777·0)	(5 910·4–7 761·6)	(8 301·1–10 808·9)	(4 440·8–7 036·7)	(6 824·6–8 537·4)	(4 201·6–5 773·9)	(15 312·1–23 793·4)
Male	5 090·0	16 571·7	6 803·7	4 981·1	3 719·9	5 072·5	6 461·6	3 262·2	7 709·6	3 298·4	9 185·9
	(4 420·2–5 840·4)	(14 872·2–18 703·3)	(5 888·9–7 916·8)	(4 066·8–6 127·6)	(3 337·8–4 126·7)	(4 472·1–5 684·7)	(5 668·8–7 428·7)	(2 772·9–3 835·4)	(6 831·2–8 735·5)	(2 872·8–3 750·0)	(7 593·7–10 986·6)
Female	11 938·7	16 562·5	14 408·8	12 102·7	5 962·2	8 510·0	11 063·2	6 927·7	7 581·0	5 971·3	23 479·3
	(9 683·4–14 328·7)	(14 678·6–18 904·5)	(11 958·6–17 192·1)	(9 217·0–15 617·0)	(5 030·7–7 033·0)	(7 109·1–9 955·6)	(9 450·4–12 687·2)	(5 319·3–8 788·1)	(6 709·8–8 595·2)	(4 933·9–7 247·5)	(18 232·3–28 949·6)
**90-94 years**											
Total	9 901·8	16 895·1	12 816·7	10 130·8	5 380·5	7 483·6	10 096·4	6 157·3	8 458·7	5 528·0	21 466·8
	(8 464·9–11 438·9)	(15 288·6–18 891·6)	(11 088·7–14 627·8)	(8 220·0–12 558·7)	(4 788·9–6 033·8)	(6 715·7–8 385·1)	(9 011·6–11 278·5)	(5 231·1–7 380·3)	(7 682·8–9 416·1)	(4 818·3–6 253·6)	(17 780·0–25 617·5)
Male	5 213·0	15 774·2	7 404·9	5 603·2	4 171·9	5 627·6	6 936·8	3 375·5	8 357·5	3 711·3	9 877·7
	(4 694·3–5 876·1)	(14 335·4–17 499·2)	(6 500·6–8 371·3)	(4 783·9–6 639·3)	(3 789·3–4 610·3)	(5 083·0–6 240·8)	(6 230·8–7 736·9)	(2 969·7–3 871·2)	(7 528·6–9 416·1)	(3 344·6–4 099·1)	(8 414·0–11 437·7)
Female	12 513·8	16 999·0	14 991·1	12 682·6	6 094·5	9 759·2	11 517·2	8 355·3	8 508·6	6 669·7	24 832·6
	(10 546·7–14 578·2)	(15 322·8–19 066·2)	(12 819·9–17 338·6)	(10 023·7–15 922·8)	(5 248·0–7 027·3)	(8 543·2–11 245·8)	(10 145·7–12 995·0)	(6 937·7–10 176·3)	(7 643·9–9 511·0)	(5 678·7–7 710·9)	(20 489·3–29 834·1)

**95+ years**											
Total	9 551·1	14 159·0	12 728·2	10 171·4	5 480·5	8 157·4	9 887·9	5 478·5	10 301·4	6 248·1	21 086·6
	(7 722·9–11 937·4)	(11 986·2–16 922·7)	(10 343·0–15 504·2)	(7 481·4–14 237·4)	(4 698·2–6 451·8)	(6 988·6–9 578·2)	(8 089·5–11 937·4)	(4 417·0–6 809·3)	(8 926·9–12 038·0)	(5 100·9–7 692·8)	(16 339·0–28 115·7)
Male	5 295·2	12 285·4	7 931·2	6 163·1	4 844·6	6 778·6	7 153·5	3 782·1	9 685·2	4 580·5	10 259·9
	(4 559·9–6 131·0)	(10 225·8–14 662·0)	(6 559·2–9 563·1)	(4 921·3–7 731·0)	(4 210·6–5 545·2)	(5 910·2–7 735·3)	(5 980·6–8 542·7)	(3 107·1–4 460·7)	(8 140·3–11 336·7)	(3 905·0–5 375·3)	(8 355·3–12 671·2)
Female	12 095·9	14 615·5	14 146·0	11 928·7	5 750·2	11 142·4	10 741·5	9 513·6	10 530·9	7 379·5	22 772·1
	(9 594·7–15 478·2)	(12 322·1–17 538·1)	(11 447·0–17 501·8)	(8 507·7–17 090·3)	(4 778·6–6 988·5)	(9 059·1–13 687·2)	(8 682·0–13 230·8)	(7 192·2–12 643·3)	(9 058·8–12 546·7)	(5 818·4–9 248·7)	(17 557·7–30 486·2)

95% UI=95% uncertainty interval.

Table S11 | Changes (in %) in age-standardised DALY rates from 1990 to 2021 by sex and Level 3 injury causes.

	Change (in %) in age-standardised DALY rates from 1990 to 2021										
	**ASEAN**	**Brunei**	**Cambodia**	**Indonesia**	**Laos**	**Malaysia**	**Myanmar**	**Philippines**	**Singapore**	**Thailand**	**Viet Nam**
**Adverse effects of medical treatment**											
Total	-57·6	-17·4	-60·4	-57·4	-66·0	-55·4	-65·6	-37·1	-33·6	-55·5	-56·1
	(-64·5–-48·4)	(-53·2–16·3)	(-72·6–-42·1)	(-65·8–-46·0)	(-77·4–-45·6)	(-67·9–-40·2)	(-75·5–-45·8)	(-50·2–-23·5)	(-39·1–-28·3)	(-64·8–-43·5)	(-67·7–-42·4)
Male	-53·0	-12·3	-54·4	-53·1	-63·6	-53·8	-59·8	-36·3	-24·2	-49·9	-51·1
	(-61·8–-38·0)	(-55·8–38·7)	(-70·5–-29·2)	(-64·7–-36·1)	(-76·9–-33·3)	(-69·1–-29·2)	(-73·1–-24·7)	(-50·7–-19·8)	(-30·4–-18·0)	(-62·3–-31·2)	(-66·3–-30·6)
Female	-62·7	-22·7	-66·5	-62·0	-68·7	-57·6	-70·7	-37·7	-39·9	-61·4	-63·7
	(-70·3–-49·9)	(-56·8–7·6)	(-77·8–-46·6)	(-72·6–-46·6)	(-80·3–-43·6)	(-69·4–-36·0)	(-80·0–-52·2)	(-52·0–-15·9)	(-46·1–-33·9)	(-71·1–-45·2)	(-74·0–-48·5)
**Animal contact**											
Total	-49·3	-35·8	-46·5	-42·0	-55·1	-19·7	-55·3	-48·9	-31·2	-57·8	-46·4
	(-61·3–-24·0)	(-44·6–-25·2)	(-62·5–-9·7)	(-56·5–-5·4)	(-69·9–-18·8)	(-35·1–2·1)	(-71·4–-14·0)	(-57·6–-30·9)	(-34·8–-28·3)	(-67·5–-45·6)	(-60·9–-25·6)
Male	-47·5	-39·5	-48·0	-41·2	-57·4	-19·6	-52·4	-51·3	-30·9	-51·0	-43·0
	(-64·2–-4·6)	(-49·7–-25·6)	(-64·8–5·3)	(-59·3–8·3)	(-73·3–-1·2)	(-43·7–11·6)	(-75·7–42·0)	(-61·7–-27·7)	(-36·1–-27·1)	(-64·6–-31·5)	(-64·8–-6·4)
Female	-52·8	-23·2	-45·0	-43·8	-51·1	-22·2	-58·8	-43·1	-31·4	-67·1	-50·8
	(-61·6–-27·1)	(-30·6–-17·7)	(-61·6–3·1)	(-55·8–-19·5)	(-67·5–2·2)	(-44·6–4·7)	(-72·9–-3·4)	(-52·7–-24·8)	(-34·5–-28·3)	(-76·0–-54·3)	(-64·9–-24·3)
**Conflict and terrorism**											
Total	-36·9	NA	-74·3	-58·0	-98·0	0·5	87·4	-67·1	NA	85·7	-44·6
	(-49·3–-23·9)	NA	(-81·7–-70·2)	(-61·8–-52·0)	(-98·7–-96·3)	(-45·0–144·8)	(16·9–167·5)	(-70·8–-63·4)	NA	(49·7–114·8)	(-50·6–-38·1)
Male	-37·7	NA	-74·7	-58·5	-98·1	-0·1	90·3	-67·6	NA	88·0	-44·9
	(-49·6–-25·5)	NA	(-81·6–-70·8)	(-62·0–-52·9)	(-98·8–-96·4)	(-45·8–144·4)	(20·3–168·0)	(-71·4–-63·9)	NA	(51·2–117·0)	(-50·9–-38·2)
Female	-38·5	NA	-72·7	-59·7	-97·8	-5·3	76·8	-66·2	NA	83·3	-46·4
	(-50·6–-24·6)	NA	(-80·9–-68·4)	(-63·8–-53·3)	(-98·7–-95·9)	(-46·5–129·9)	(7·9–161·9)	(-70·5–-61·8)	NA	(45·8–118·9)	(-53·8–-38·9)
**Drowning**											
Total	-60·6	-52·8	-60·5	-62·8	-68·8	-42·2	-71·5	-50·9	-63·1	-47·1	-57·7
	(-66·7–-53·2)	(-60·1–-42·7)	(-71·2–-46·6)	(-71·3–-52·0)	(-77·7–-55·0)	(-51·5–-30·4)	(-79·7–-59·2)	(-59·1–-41·8)	(-66·0–-59·8)	(-57·6–-34·0)	(-66·5–-45·4)
Male	-59·1	-54·2	-59·1	-60·9	-68·2	-41·8	-71·2	-51·0	-65·6	-36·9	-56·8
	(-65·6–-50·7)	(-61·6–-43·1)	(-70·6–-42·9)	(-70·3–-48·5)	(-78·2–-53·5)	(-52·6–-28·8)	(-79·8–-57·8)	(-59·7–-41·5)	(-68·5–-62·3)	(-50·5–-20·4)	(-66·2–-44·3)
Female	-64·8	-40·8	-65·6	-67·0	-70·7	-47·9	-72·3	-51·1	-44·4	-66·9	-61·3
	(-71·1–-57·1)	(-52·2–-25·5)	(-76·4–-51·9)	(-75·7–-55·9)	(-81·3–-54·1)	(-59·0–-31·8)	(-82·3–-58·9)	(-60·0–-40·1)	(-49·6–-38·8)	(-75·0–-55·8)	(-71·4–-48·1)
**Environmental heat and cold exposure**											
Total	-25·7	-14·1	-27·2	-29·2	-37·0	-30·5	-32·7	-25·4	-8·1	-20·8	-33·2
	(-32·3–-14·9)	(-28·6–3·0)	(-40·2–-9·1)	(-36·1–-19·0)	(-52·4–-13·5)	(-36·3–-24·9)	(-46·1–-12·7)	(-40·1–-7·4)	(-11·3–-4·5)	(-33·4–-7·5)	(-40·7–-26·1)
Male	-22·0	-13·9	-24·5	-27·9	-38·2	-30·5	-28·6	-20·9	-8·1	-14·8	-27·8
	(-31·4–-4·8)	(-31·0–14·0)	(-43·2–11·2)	(-38·4–-11·3)	(-57·1–-1·9)	(-37·0–-23·5)	(-47·6–6·9)	(-40·7–5·1)	(-12·5–-4·2)	(-32·3–6·7)	(-33·1–-21·8)
Female	-30·8	-13·5	-31·0	-31·4	-36·4	-30·7	-36·8	-33·6	-9·2	-28·1	-35·8
	(-34·8–-26·8)	(-28·6–-3·6)	(-40·8–-20·3)	(-34·4–-28·4)	(-49·7–-21·7)	(-37·0–-24·4)	(-48·1–-26·1)	(-40·3–-26·8)	(-14·5–-3·9)	(-35·6–-20·8)	(-44·5–-27·4)
**Exposure to forces of nature**											
Total	-37·3	NA	NA	258·2	1 217·1	6 094·0	654 122·6	-83·2	NA	-2·0	-58·2
	(-48·0–-21·5)	NA	NA	(203·5–332·9)	(1 085·1–1 338·0)	(4 446·8–8 269·6)	(604 023·5–709 453·8)	(-85·0–-80·5)	NA	(-17·7–18·3)	(-64·6–-50·8)
Male	-47·4	NA	NA	229·3	1 272·6	7 348·5	615 207·8	-84·5	NA	-20·1	-63·7

	(-56·3–-36·6)	NA	NA	(183·4–286·6)	(1 078·5–1 491·0)	(5 379·9–10 110·0)	(543 015·1–691 784·6)	(-86·1–-82·8)	NA	(-32·2–-7·3)	(-69·1–-58·0)
Female	-30·4	NA	NA	279·3	1 215·4	5 565·2	662 544·0	-82·2	NA	7·0	-54·7
	(-43·3–-12·0)	NA	NA	(215·3–365·4)	(1 070·5–1 373·5)	(4 069·2–7 561·2)	(607 347·4–722 650·4)	(-84·4–-79·3)	NA	(-11·3–32·2)	(-62·3–-45·7)
**Exposure to mechanical forces**											
Total	-45·3	-24·9	-34·8	-42·5	-44·5	-38·9	-46·8	-30·9	-30·6	-55·1	-47·2
	(-52·2–-38·1)	(-31·0–-18·0)	(-47·0–-18·9)	(-49·0–-34·5)	(-55·9–-29·3)	(-47·2–-32·4)	(-57·3–-31·1)	(-39·1–-22·1)	(-36·7–-25·1)	(-65·6–-43·6)	(-60·6–-29·8)
Male	-45·6	-23·1	-38·2	-40·1	-48·2	-42·6	-46·8	-32·6	-37·1	-51·4	-50·2
	(-55·0–-35·1)	(-30·5–-13·1)	(-50·6–-20·5)	(-51·7–-28·3)	(-59·7–-32·3)	(-52·1–-34·5)	(-58·1–-28·9)	(-42·6–-21·2)	(-43·5–-31·5)	(-65·6–-37·3)	(-65·4–-28·3)
Female	-46·2	-25·6	-34·0	-47·2	-37·7	-32·3	-39·8	-27·5	-11·2	-61·4	-44·8
	(-51·5–-39·8)	(-31·0–-20·1)	(-46·1–-17·0)	(-54·4–-35·4)	(-50·7–-19·7)	(-38·1–-27·9)	(-57·5–-17·9)	(-32·1–-22·5)	(-14·9–-8·4)	(-69·8–-52·2)	(-54·0–-34·6)
**Falls**											
Total	-12·3	-16·4	6·0	-16·9	-8·9	-11·9	-11·8	-12·0	-19·7	-0·6	3·5
	(-21·0–-2·6)	(-23·9–-8·0)	(-14·7–31·9)	(-28·0–-3·7)	(-34·4–24·3)	(-20·8–-4·3)	(-33·7–14·2)	(-19·7–-3·0)	(-24·4–-15·7)	(-16·3–15·6)	(-14·6–22·1)
Male	-9·4	-17·6	-0·3	-11·4	-15·6	-14·6	-10·4	-13·2	-29·6	12·0	-2·7
	(-20·2–1·9)	(-28·0–-5·5)	(-23·2–31·1)	(-26·0–8·2)	(-41·8–16·7)	(-25·8–-5·7)	(-35·2–18·4)	(-23·8–0·1)	(-33·7–-26·2)	(-10·7–38·8)	(-23·0–21·7)
Female	-17·7	-10·8	8·6	-24·1	1·6	-8·5	-11·8	-12·6	-2·8	-22·2	6·0
	(-25·5–-7·3)	(-17·4–-4·5)	(-11·9–36·6)	(-35·5–-10·7)	(-26·1–40·2)	(-16·9–-0·3)	(-32·5–14·9)	(-19·8–-4·8)	(-7·9–1·4)	(-35·4–-1·7)	(-10·5–23·1)
**Fire, heat, and hot substances**											
Total	-47·1	-48·9	-59·6	-46·4	-64·2	-50·1	-60·6	-42·4	-22·3	-33·1	-49·6
	(-53·1–-38·6)	(-56·7–-41·4)	(-68·2–-46·5)	(-55·2–-34·8)	(-72·4–-51·9)	(-56·9–-42·1)	(-69·0–-48·0)	(-48·5–-34·8)	(-31·7–-15·2)	(-49·8–-15·9)	(-59·2–-38·3)
Male	-38·2	-48·5	-54·5	-34·9	-60·5	-49·5	-46·9	-39·4	-23·0	-23·1	-43·4
	(-46·6–-25·4)	(-58·8–-37·8)	(-66·0–-33·5)	(-46·0–-11·0)	(-71·1–-38·8)	(-58·4–-38·4)	(-62·4–-22·6)	(-47·0–-28·0)	(-33·5–-15·1)	(-48·6–6·2)	(-54·0–-30·6)
Female	-55·1	-48·7	-64·9	-55·8	-68·3	-51·6	-67·9	-46·2	-22·2	-44·4	-55·1
	(-62·4–-45·7)	(-58·0–-39·5)	(-74·4–-50·6)	(-65·3–-42·6)	(-76·2–-54·2)	(-58·2–-43·1)	(-75·6–-55·2)	(-53·0–-37·6)	(-30·5–-15·7)	(-55·6–-32·0)	(-66·5–-37·7)
**Foreign body**											
Total	-34·6	-21·3	-42·0	-45·3	-48·7	-30·6	-43·5	-25·2	-50·6	19·9	-36·4
	(-44·9–-11·0)	(-37·3–-0·2)	(-57·9–3·7)	(-58·5–-11·7)	(-63·5–5·0)	(-43·9–-11·8)	(-59·6–-2·3)	(-36·2–-3·8)	(-56·2–-44·7)	(-13·5–60·0)	(-53·9–-10·5)
Male	-33·2	-19·2	-37·7	-52·8	-48·3	-29·9	-41·6	-27·1	-53·7	48·1	-26·7
	(-43·7–-8·5)	(-37·9–6·7)	(-56·5–6·6)	(-63·4–-24·9)	(-65·4–7·1)	(-44·0–-10·3)	(-58·5–11·4)	(-40·1–-2·9)	(-59·3–-47·6)	(-11·5–101·0)	(-44·3–-1·8)
Female	-35·8	-22·6	-48·4	-36·1	-49·6	-32·8	-45·5	-21·6	-45·5	-13·9	-40·9
	(-49·5–-5·8)	(-45·7–6·6)	(-65·5–5·3)	(-55·2–13·1)	(-69·3–10·5)	(-50·4–-8·3)	(-64·0–-2·8)	(-36·9–4·2)	(-51·8–-38·8)	(-38·8–22·9)	(-63·1–-7·9)
**Interpersonal violence**											
Total	-40·4	-43·1	-35·0	-21·0	-50·3	-18·0	-58·7	-44·6	-63·0	-47·8	-25·9
	(-47·3–-33·3)	(-51·1–-33·4)	(-51·0–-12·5)	(-30·5–-9·7)	(-63·5–-31·4)	(-31·1–-5·1)	(-67·4–-47·8)	(-53·5–-34·7)	(-65·9–-59·8)	(-65·3–-29·9)	(-45·0–-0·9)
Male	-42·0	-48·2	-36·1	-28·9	-52·2	-18·0	-59·2	-43·6	-68·4	-47·8	-29·5
	(-49·8–-33·7)	(-57·3–-37·1)	(-53·4–-11·6)	(-40·1–-14·0)	(-65·5–-33·7)	(-35·3–-2·4)	(-69·2–-46·3)	(-54·0–-32·4)	(-71·0–-65·4)	(-66·8–-27·5)	(-51·7–-0·2)
Female	-38·2	-30·3	-41·7	-6·3	-49·4	-25·1	-56·8	-54·6	-54·8	-45·3	-25·1
	(-44·0–-31·0)	(-39·6–-17·4)	(-58·5–-12·3)	(-16·7–8·8)	(-66·8–-14·8)	(-35·3–-12·9)	(-67·0–-43·9)	(-62·3–-46·0)	(-58·6–-51·0)	(-62·7–-24·8)	(-37·4–-10·7)
**Other transport injuries**											
Total	-38·4	-67·9	-31·7	-41·8	-47·6	-9·7	-61·3	-20·2	-38·0	-39·8	-20·3
	(-51·6–-14·3)	(-74·4–-59·1)	(-51·5–-2·6)	(-61·7–-2·6)	(-66·2–-19·0)	(-40·2–28·9)	(-73·0–-45·3)	(-38·9–1·0)	(-45·0–-31·3)	(-57·1–-11·8)	(-46·0–29·7)
Male	-36·1	-65·9	-32·1	-40·0	-48·0	-11·5	-57·8	-14·6	-44·3	-36·9	-20·0
	(-50·8–-7·3)	(-74·2–-55·2)	(-53·9–0·3)	(-62·0–6·8)	(-68·1–-11·9)	(-43·2–31·1)	(-72·5–-37·9)	(-38·7–13·6)	(-51·4–-36·6)	(-56·4–-2·7)	(-47·9–38·3)

Female	-51·6	-71·8	-42·9	-51·2	-52·1	-18·2	-70·9	-43·4	-25·6	-53·9	-40·9
	(-62·8–-33·1)	(-77·9–-61·9)	(-61·6–-12·5)	(-70·1–-20·7)	(-70·1–-16·7)	(-46·8–25·9)	(-78·7–-60·1)	(-54·2–-27·0)	(-30·8–-21·5)	(-68·0–-34·6)	(-57·1–-14·6)
**Other unintentional injuries**											
Total	-54·2	-42·5	-51·9	-47·7	-61·6	-54·2	-64·5	-43·2	-51·2	-52·4	-56·9
	(-60·2–-44·4)	(-52·1–-32·8)	(-64·5–-31·3)	(-59·1–-34·4)	(-72·2–-42·5)	(-62·3–-44·5)	(-73·7–-50·6)	(-52·9–-29·1)	(-58·3–-44·9)	(-62·7–-38·5)	(-68·4–-41·7)
Male	-55·3	-47·4	-52·9	-51·0	-63·5	-57·8	-61·2	-44·2	-63·4	-50·0	-60·1
	(-62·5–-43·8)	(-57·2–-34·2)	(-67·1–-28·3)	(-63·3–-34·9)	(-73·7–-43·8)	(-66·1–-45·4)	(-72·4–-43·2)	(-55·5–-25·2)	(-69·8–-57·6)	(-62·1–-33·1)	(-71·6–-44·0)
Female	-53·2	-27·6	-57·3	-40·1	-58·5	-45·4	-70·4	-41·8	-21·2	-59·1	-49·9
	(-60·7–-44·6)	(-32·7–-23·8)	(-67·2–-44·4)	(-49·0–-29·7)	(-71·5–-39·3)	(-55·4–-35·9)	(-78·2–-59·5)	(-49·2–-32·0)	(-25·7–-17·9)	(-66·9–-49·5)	(-62·0–-37·4)
**Poisonings**											
Total	-41·5	-47·1	-41·5	-25·2	-54·1	-48·7	-55·7	-36·9	-45·1	-24·3	-43·2
	(-53·4–-21·6)	(-55·5–-35·7)	(-57·6–-14·2)	(-44·6–7·1)	(-69·0–-26·0)	(-64·2–-30·7)	(-69·1–-32·1)	(-46·2–-22·3)	(-53·5–-36·9)	(-51·5–10·1)	(-65·5–-5·5)
Male	-40·0	-46·6	-41·5	-25·7	-56·5	-47·9	-53·3	-38·7	-54·9	-21·1	-45·9
	(-54·3–-14·7)	(-57·0–-29·7)	(-59·2–-11·7)	(-47·0–10·2)	(-71·0–-28·5)	(-65·7–-25·0)	(-69·1–-19·5)	(-50·3–-20·3)	(-63·8–-46·3)	(-54·4–21·6)	(-68·7–-4·9)
Female	-49·4	-47·8	-47·2	-30·2	-50·5	-54·2	-58·5	-33·2	-31·6	-31·0	-47·3
	(-58·7–-36·5)	(-57·7–-36·4)	(-64·6–-15·2)	(-42·1–-8·5)	(-69·6–-15·8)	(-68·1–-37·3)	(-71·4–-37·2)	(-43·4–-17·1)	(-40·5–-20·9)	(-52·3–1·3)	(-62·4–-23·6)
**Police conflict and executions**											
Total	1 447·7	-86·5	62·1	16·7	0·7	2 044·3	13 740·6	272·3	-70·6	619·4	22·7
	(824·9–6 316·6)	(-91·6–-41·7)	(-12·9–694·6)	(-11·2–157·3)	(-35·5–223·8)	(1 367·3–3 611·6)	(6 283·8–123 290·6)	(129·5–1 093·6)	(-79·8–-57·6)	(371·1–2 683·9)	(-27·6–313·1)
Male	909·4	-87·5	28·9	9·5	-10·7	1 410·6	9 045·3	179·8	-70·9	413·2	6·0
	(514·3–4 041·9)	(-92·2–-40·7)	(-29·8–473·4)	(-16·9–124·6)	(-42·2–162·3)	(935·2–2 335·0)	(4 105·1–90 610·8)	(77·9–741·5)	(-80·0–-57·8)	(238·1–1 775·7)	(-37·0–210·2)
Female	41 987·2	-26·9	1 539·9	333·8	526·6	96 821·2	261 929·6	6 290·4	-79·0	25 589·2	1 254·0
	(21 482·5–128 727·8)	(-55·1–26·4)	(661·5–4 759·6)	(148·3–935·9)	(183·9–1 995·3)	(49 534·3–238 525·4)	(111 866·6–748 144·7)	(3 244·5–39 242·4)	(-86·9–-65·3)	(10 555·6–258 317·5)	(511·1–3 929·2)
**Road injuries**											
Total	-39·6	-66·9	-31·3	-46·9	-43·3	-23·8	-53·9	-17·7	-78·1	-22·2	-25·2
	(-46·4–-32·1)	(-70·8–-62·0)	(-48·2–-9·3)	(-56·5–-34·0)	(-58·9–-23·3)	(-30·2–-15·7)	(-64·6–-38·3)	(-31·1–-2·2)	(-79·9–-76·3)	(-37·5–-3·2)	(-42·3–-4·2)
Male	-37·0	-68·0	-28·7	-44·9	-42·8	-25·1	-47·8	-12·3	-79·2	-19·3	-22·9
	(-44·6–-28·5)	(-72·3–-62·8)	(-47·1–-5·2)	(-56·5–-30·0)	(-60·8–-18·7)	(-32·9–-16·2)	(-61·1–-28·8)	(-29·5–7·2)	(-80·9–-77·6)	(-35·6–1·2)	(-42·2–0·8)
Female	-49·6	-63·3	-44·6	-54·5	-47·9	-26·9	-64·5	-35·8	-73·9	-30·9	-38·6
	(-56·1–-41·3)	(-69·0–-51·4)	(-59·3–-21·4)	(-64·1–-41·5)	(-64·1–-22·8)	(-41·0–-14·4)	(-73·8–-52·9)	(-47·6–-22·9)	(-76·6–-71·1)	(-51·2–-9·9)	(-54·8–-17·6)
**Self-harm**											
Total	-26·7	-27·3	-30·1	-26·6	-48·1	-26·7	-39·4	-14·3	-51·5	-8·9	-24·2
	(-36·5–-13·1)	(-43·3–-4·8)	(-50·6–8·0)	(-41·7–-2·2)	(-64·6–-14·7)	(-37·2–-13·8)	(-57·7–-6·4)	(-27·9–2·4)	(-55·4–-47·9)	(-31·9–16·9)	(-45·6–11·3)
Male	-20·4	-22·9	-24·1	-21·1	-48·3	-21·5	-32·4	-13·8	-47·2	5·2	-17·0
	(-32·2–-5·8)	(-40·4–0·9)	(-47·1–15·4)	(-40·6–7·8)	(-65·9–-14·8)	(-33·4–-5·8)	(-53·8–6·1)	(-30·3–5·5)	(-51·6–-42·7)	(-23·7–39·1)	(-42·6–24·6)
Female	-42·8	-39·9	-44·2	-38·0	-50·5	-45·5	-55·9	-19·1	-58·4	-43·5	-39·1
	(-51·9–-18·4)	(-55·6–-11·6)	(-63·6–8·9)	(-54·4–0·9)	(-70·0–7·0)	(-58·5–-25·1)	(-71·2–-5·2)	(-35·9–3·8)	(-61·6–-55·2)	(-61·2–-17·7)	(-57·7–0·3)

95% UI=95% uncertainty interval. NA=not available.

Authors' affiliations

Division of Family Medicine (S C C van der Lubbe PhD), Department of Medicine (L Chong MSc, B Chong MBBS, Prof Y Lim PhD), Yong Loo Lin School of Medicine (L Goh PhD, N W Chew MD, Prof N Venketasubramanian MSc, M Ng PhD), Cardiovascular Metabolic Translational Research Program (M Dalakoti MPH), Saw Swee Hock School of Public Health (S Ramazanu PhD, Prof S Yi PhD), National University of Singapore, Singapore, Singapore; Office of Senior Advisor (L Chong MSc), National University Health System, Singapore, Singapore; Institute for Health Metrics and Evaluation (Prof S I Hay FMedSci, C Bisignano MPH, X Dai PhD, Prof C J L Murray DPhil, K L Ong PhD, M Ng PhD), Department of Health Metrics Sciences, School of Medicine (Prof S I Hay FMedSci, X Dai PhD, Prof C J L Murray DPhil), University of Washington, Seattle, WA, USA; Docere Research, Seattle, WA, USA (S L James MD); Department of Oral Pathology and Microbiology (S Acharya MDS), JSS Academy of Higher Education and Research, Mysuru, India; Department of Public Health (Q Adnani PhD), Padjadjaran University, Bandung, Indonesia; Department of Assistance Medical Sciences (N Ahmed PhD), University of Tabuk, Tabuk, Saudi Arabia; Department of Medical Microbiology and Parasitology (N Ahmed PhD), School of Health Sciences (S Shaharudin PhD), Universiti Sains Malaysia, Kota Bharu, Malaysia; Faculty of Medicine and Public Health (B Aji DrPH), Jenderal Soedirman University, Purwokerto, Indonesia; Department of Medicine (J U Almazan PhD), Nazarbayev University, Astana, Kazakhstan; Department of Health Policy and Administration (C T Antonio MD, E A Faraon MD, Prof F B Garcia PhD), Department of Epidemiology and Biostatistics (A C Bermudez MD), Department of Environmental and Occupational Health (C M Estrada PhD, P R Hernandez MD), Department of Neurosciences (Prof R G Jamora PhD), University of the Philippines Manila, Manila, Philippines; Department of Applied Social Sciences (C T Antonio MD), Hong Kong Polytechnic University, Hong Kong, China; Department of Surgery (S Anwar PhD), Department of Pharmacology (I Fitriana PhD), Department of Medical Surgical Nursing (A L Wicaksana MS), Gadjah Mada University, Yogyakarta, Indonesia; School of Traditional Chinese Medicine (M Aslam PhD, Y Kim PhD), Xiamen University Malaysia, Sepang, Malaysia; International Medical School (A A Baig PhD), Management and Science University, Alam, Malaysia; Department of Epidemiology (A C Bermudez MD), Brown University, Providence, RI, USA; Department of Clinical Pharmacy (A N Bitar PhD), Universiti Sultan Zainal Abidin, Besut, Malaysia; College of Public Health, Medical, and Veterinary Sciences (M Cenderadewi MPHTM), James Cook University, Townsville, QLD, Australia; Department of Public Health (M Cenderadewi MPHTM), University of Mataram, Mataram, Indonesia; Faculty of Humanities and Health Sciences (H Chen MSc), Curtin University, Miri, Malaysia; Department of Public Health and Primary Care (M Dalakoti MPH), University of Cambridge, Cambridge, UK; School of Health Sciences (H A Edinur PhD), University of Science Malaysia, Kubang Kerian, Malaysia; Centre for Public Health, Equity and Human Flourishing (N K Fauk PhD), Torrens University Australia, Adelaide, SA, Australia; Institute of Resource Governance and Social Change, Kupang, Indonesia (N K Fauk PhD); Graduate Institute of Injury Prevention and Control (N Fridayani MSc), School of Nursing (M Kurniasari PhD, A L Wicaksana MS), Graduate Institute of Biomedical Informatics (D N A Ningrum PhD), Taipei Medical University, Taipei, Taiwan; Department of Epidemiology Population Biostatistics and Health Promotion (A Hargono PhD), Department of Advanced Nursing (E M M Has PhD), Universitas Airlangga (Airlangga University), Surabaya, Indonesia; School of Nursing and Midwivery (E M M Has PhD), La Trobe University, Bundoora, VIC, Australia; Department of Clinical Pharmacy & Pharmacy Practice (Prof N Ismail PhD), Asian Institute of Medicine, Science and Technology, Bedong, Malaysia; Malaysian Academy of Pharmacy, Puchong, Malaysia (Prof N Ismail PhD); Department of Research and Academic

Affairs (V Jaiswal MD), Larkin Community Hospital, South Miami, FL, USA; Department of Medicine (V Jaiswal MD), AMA School of Medicine, Makati, Philippines; Institute for Neurosciences (Prof R G Jamora PhD), St. Luke's Medical Center, Bonifacio Global City, Philippines; Rothschild Foundation Hospital (Prof J B Jonas MD), Institut Français de Myopie, Paris, France; Singapore Eye Research Institute (Prof J B Jonas MD), Singapore Eye Research Institute, Singapore, Singapore; Faculty of Dentistry (Prof K K Kanmodi DDS, S Selvaraj PhD), University of Puthisastra, Phnom Penh, Cambodia; Office of the Executive Director (Prof K K Kanmodi DDS), Cephas Health Research Initiative Inc, Ibadan, Nigeria; Department of Public Health and Health Policy (I Khaing MPH), Hiroshima University, Hiroshima, Japan; School of Applied Science (C Kua PhD), Republic Polytechnic, Singapore, Singapore; Faculty of Medicine and Health Science (M Kurniasari PhD), Universitas Kristen Satya Wacana, Salatiga, Indonesia; National Research and Innovation Agency (BRIN), Jakarta, Indonesia (A Kusnali MA); Department of Public Health and Epidemiology (D Kusuma DSc), Khalifa University of Science and Technology, Abu Dhabi, United Arab Emirates; Faculty of Public Health (D Kusuma DSc), University of Indonesia, Depok, Indonesia; Institute of Health Policy and Development Studies (Prof H Lam PhD), National Institutes of Health, Manila, Philippines; School of Pharmacy (Prof S W H Lee PhD), Jeffrey Cheah School of Medicine and Health Sciences (Prof R R Marzo MD), Monash University, Subang Jaya, Malaysia; School of Pharmacy (Prof S W H Lee PhD), Taylor's University Lakeside Campus, Subang Jaya, Malaysia; Centre for Public Health and Wellbeing (Z Ma PhD), University of the West of England, Bristol, UK; Faculty of Humanities and Health Sciences (Prof R R Marzo MD), Curtin University, Sarawak, Malaysia; School of Medical Sciences (Prof K Musa PhD), Science University of Malaysia, Kubang Kerian, Malaysia; Faculty of Pharmacy (Prof F Nainu PhD), Hasanuddin University, Makassar, Indonesia; Public Health Department (D N A Ningrum PhD), Universitas Negeri Semarang, Kota Semarang, Indonesia; Department of Public Health (S Ong FAMS), Ministry of Health, Bandar Seri Begawan, Brunei; Institute of Health Sciences (S Ong FAMS), Universiti Brunei Darussalam, Bandar Seri Begawan, Brunei; Center for Research and Innovation (V F Pepito MSc), Ateneo De Manila University, Pasig City, Philippines; Center of Excellence in Genomics and Precision Dentistry (T Porntaveetus PhD), Chulalongkorn University, Bangkok, Thailand; Health Sciences Department (D R A Pribadi MSc), Muhammadiyah University of Surakarta, Sukoharjo, Indonesia; School of Nursing & Health Sciences (S Ramazanu PhD), Hong Kong Metropolitan University, Hong Kong, China; Faculty of Medicine (B Roy PhD), Quest International University Perak, Ipoh, Malaysia; Institute of Epidemiology and Preventive Medicine (Y L Samodra PhD), National Taiwan University, Taipei, Taiwan; Benang Merah Research Center (BMRC), Minahasa Utara, Indonesia (Y L Samodra PhD); Dr. D. Y. Patil Dental College & Hospital (S Selvaraj PhD), Dr. D. Y. Patil Vidyapeeth, Pune (Deemed to be University), Pune, India; Department of Internal Medicine (R Sinto MD), University of Indonesia, Jakarta Pusat, Indonesia; Department of Internal Medicine (R Sinto MD), Dr. Cipto Mangunkusumo National Hospital, Jakarta Pusat, Indonesia; Faculty of Public Health (Prof S Solikhah DrPH), Universitas Ahmad Dahlan, Yogyakarta, Indonesia; Department of Public Health and Community Medicine (Prof C T Sreeramareddy MD), International Medical University, Kuala Lumpur, Malaysia; Medical and Diagnostic Research Centre (Prof C T Sreeramareddy MD), University of Hail, Hail, Saudi Arabia; Department of Medical Sciences (Prof V Subramaniyan PhD), Sunway University, Subang Jaya, Malaysia; Praboromarajchanok Institute (T Sukaew PhD), Ministry of Public Health, Nonthaburi, Thailand; National Research and Innovation Agency, Jakarta, Indonesia (I U Tarigan PhD); Faculty of Public Health (J H V Ticoalu MPH), Universitas Sam Ratulangi (Sam Ratulangi University), Manado, Indonesia; Raffles Neuroscience Centre (Prof N Venketasubramanian MSc), Raffles Hospital, Singapore, Singapore; Department of Physical Therapy (T Wiangkham PhD), Naresuan University,

Phitsanulok, Thailand; KHANA Center for Population Health Research, Phnom Penh, Cambodia (Prof S Yi PhD); Department of Health Policy and Management (Prof Dr. M Z Younis PhD), Jackson State University, Jackson, MS, USA; School of Business & Economics (Prof M Z Younis PhD), Universiti Putra Malaysia (University of Putra Malaysia), Kuala Lumpur, Malaysia

Authors' contributions

Providing data or critical feedback on data sources

Swetha Acharya, Qorinah Estiningtyas Sakilah Adnani, Budi Aji, Joseph Uy Almazan, Sumadi Lukman Anwar, Muhammad Shahzad Aslam, Atif Amin Baig, Muthia Cenderadewi, Nicholas WS Chew, Bryan Chong, Xiaochen Dai, Crystal Amiel M Estrada, Eka Mishbahatul Marah Has, Nahlah Elkudssiah Ismail, Vikash Jaiswal, Spencer L James, Jost B Jonas, Yun Jin Kim, Chong-Han Kua, Maria Dyah Kurniasari, Asep Kusnali, Hilton Lam, Shaun Wen Huey Lee, Zheng Feei Ma, Roy Rillera Marzo, Christopher J L Murray, Kamarul Imran Musa, Kanyin Liane Ong, Sok King Ong, Thantrira Porntaveetus, Sheena Ramazanu, Siddharthan Selvaraj, Solikhah Solikhah, Chandrashekhar T Sreeramareddy, Vetriselvan Subramaniyan, Thitiporn Sukaew, Narayanaswamy Venketasubramanian, Taweewat Wiangkham, Siyan Yi, and Mustafa Z Younis.

Developing methods or computational machinery

Xiaochen Dai, Simon I Hay, Spencer L James, Kanyin Liane Ong, and Christopher J L Murray.

Providing critical feedback on methods or results

Swetha Acharya, Qorinah Estiningtyas Sakilah Adnani, Naveed Ahmed, Budi Aji, Joseph Uy Almazan, Sumadi Lukman Anwar, Muhammad Shahzad Aslam, Atif Amin Baig, Amiel Nazer C Bermudez, Muthia Cenderadewi, Hana Chen, Nicholas WS Chew, Bryan Chong, Xiaochen Dai, Mayank Dalakoti, Hisham Atan Edinur, Crystal Amiel M Estrada, Emerito Jose A Aquino Faraon, Ida Fitriana, Ni Kadek Yuni Fridayani, Fernando Barroga Garcia, Lay Hoon Goh, Arief Hargono, Eka Mishbahatul Marah Has, Simon I Hay, Paul Michael Rafa Hernandez, Nahlah Elkudssiah Ismail, Vikash Jaiswal, Spencer L James, Roland Dominic G Jamora, Jost B Jonas, Kehinde Kazeem Kanmodi, Inn Kynn Khaing, Yun Jin Kim, Chong-Han Kua, Maria Dyah Kurniasari, Asep Kusnali, Dian Kusuma, Hilton Lam, Shaun Wen Huey Lee, Zheng Feei Ma, Roy Rillera Marzo, Christopher J L Murray, Kamarul Imran Musa, Firzan Nainu, Dina Nur Anggraini Ningrum, Kanyin Liane Ong, Thantrira Porntaveetus, Dimas Ria Angga Pribadi, Yoseph Leonardo Samodra, Siddharthan Selvaraj, Shazlin Shaharudin, Solikhah Solikhah, Chandrashekhar T Sreeramareddy, Vetriselvan Subramaniyan, Thitiporn Sukaew, Ingan Ukur Tarigan, Jansje Henny Vera Ticoalu, Stephanie C. C. van der Lubbe, Narayanaswamy Venketasubramanian, Taweewat Wiangkham, Anggi Lukman Wicaksana, Siyan Yi, and Mustafa Z Younis.

Drafting the work or revising it critically for important intellectual content

Swetha Acharya, Qorinah Estiningtyas Sakilah Adnani, Naveed Ahmed, Carl Abelardo T Antonio, Muhammad Shahzad Aslam, Atif Amin Baig, Catherine Bisignano, Ahmad Naoras Naoras Bitar, Muthia Cenderadewi, Hana Chen, Nicholas WS Chew, Bryan Chong, Lin Siew Chong, Mayank Dalakoti, Emerito Jose A Aquino Faraon, Nelsensius Klau Fauk, Ida Fitriana, Ni Kadek Yuni Fridayani, Lay Hoon Goh, Eka Mishbahatul Marah Has, Simon I Hay, Paul Michael Rafa Hernandez, Nahlah Elkudssiah Ismail, Spencer L James, Jost B Jonas, Kehinde Kazeem Kanmodi, Yun Jin Kim, Chong-Han Kua, Maria Dyah Kurniasari, Asep Kusnali, Dian Kusuma, Hilton Lam, Shaun Wen Huey Lee, Yee Wei Lim, Zheng Feei Ma, Roy Rillera Marzo, Christopher J

L Murray, Kamarul Imran Musa, Marie Ng, Kanyin Liane Ong, Veincent Christian Filipino Pepito, Thantrira Porntaveetus, Bedanta Roy, Siddharthan Selvaraj, Shazlin Shaharudin, Robert Sinto, Chandrashekhar T Sreeramareddy, Thitiporn Sukaew, Stephanie C. C. van der Lubbe, Narayanaswamy Venketasubramanian, Taweewat Wiangkham, and Anggi Lukman Wicaksana.

Managing the estimation or publications process

Simon I Hay, Christopher J L Murray, and Marie Ng.
